# Antiproliferative Isoprenoid Derivatives from the Red Sea Alcyonacean *Xenia umbellata*

**DOI:** 10.3390/molecules26051311

**Published:** 2021-03-01

**Authors:** Hanan I. Althagbi, Fitri Budiyanto, Ahmed Abdel-Lateff, Khalid O. Al-Footy, Nahed O. Bawakid, Mohamed A. Ghandourah, Mohammad Y. Alfaifi, Serag Eldin I. Elbehairi, Walied M. Alarif

**Affiliations:** 1 Department of Chemistry, Faculty of Science, University of Jeddah, P.O. Box 13151, Jeddah 21493, Saudi Arabia; halthagbi@uj.edu.sa; 2 Department of Chemistry, Faculty of Science, King Abdulaziz University, P.O. Box 80203, Jeddah 21589, Saudi Arabia; kalfooti@kau.edu.sa (K.O.A.-F.); nbawaked@kau.edu.sa (N.O.B.); 3 Department of Marine Chemistry, Faculty of Marine Sciences, King Abdulaziz University, P.O. Box 80207, Jeddah 21589, Saudi Arabia; fitri.budiyanto@gmail.com (F.B.); mghandourah@kau.edu.sa (M.A.G.); 4 Department of Natural Products and Alternative Medicine, Faculty of Pharmacy, King Abdulaziz University, P.O. Box 80260, Jeddah 21589, Saudi Arabia; ahmedabdellateff@gmail.com; 5 Department of Pharmacognosy, Faculty of Pharmacy, Minia University, Minia 61519, Egypt; 6 Department of Biology, Faculty of Science, King Khalid University, Abha 9004, Saudi Arabia; alfaifi@kku.edu.sa (M.Y.A.); serag@kku.edu.sa (S.E.I.E.); 7 Cell Culture Laboratory, Egyptian Organization for Biological Products and Vaccines, VACSERA Holding Company, Giza 22311, Egypt

**Keywords:** Red Sea, Alcyonacea, *Xenia*, steroids, terpenes, xenicane, cytotoxicity, apoptosis

## Abstract

From the soft coral *Xenia umbellata,* seven isoprenoid derivatives were isolated, including a new xenicane diterpene, xeniolide O (**5**) and a new gorgostane derivative gorgst-3β,5α,6β,11α,20(*S*)-pentol-3-monoacetate (**7**), along with three known sesquiterpenes (**1–3**), a known diterpene (**4**), and a known steroid (**6**). The extensive analyses of the NMR, IR, and MS spectral data led to determination of their chemical structures. Compounds **1–7** displayed a cytotoxic effect against breast adenocarcinoma (MCF-7), hepatocellular carcinoma (HepG2), and cervix adenocarcinoma (HeLa), with IC_50_ values ranging between 1.5 ± 0.1–23.2 ± 1.5; 1.8 ± 0.1–30.6 ± 1.1 and 0.9 ± 0.05–12.8 ± 0.5 μg/mL, respectively. Compound **3** showed potent cytotoxic effects against MCF-7, HepG2, and HeLa with IC_50_ values = 2.4 ± 0.20, 3.1 ± 0.10 and 0.9 ± 0.05 μg/mL, respectively. Compounds **2**, **5**, and **7** displayed cytotoxic effect against Hela cells with IC_50_ values = 12.8 ± 0.50, 6.7 ± 1.00 and 11.5 ± 2.20 μg/mL, respectively. Two DNA binding dyes, acridine orange (AO) and ethidium bromide (EtBr) were used for the detection of viable, apoptotic, and necrotic cells. The early apoptotic cell death was observed in all types of treated cells. The late apoptotic cells were highly present in HepG2 cells. Compounds **5** and **7** induced a high percentage of necrosis towards HepG2 and HeLa cells. The late apoptosis was recorded as a high rate after treatment with **7** on all cancer cells.

## 1. Introduction

Cancer is an unusual growth of cells and could occur in different organs. It represents a major world health problem and also increases the global mortality rate. Additionally, it is recognized as the second leading cause of death after heart diseases [1,2]. In 2018, 9.6 million people died from cancers [3]. In men, the most common cancers were diagnosed in lung, prostate, colorectal, stomach, and liver, whereas cancers of breast, colorectal, lung, cervical, and thyroid were recorded as the most common types of cancer in women [4]. Cancer is a disease which causes a serious social burden, additionally it continues to grow globally, exerting marvelous effects on governments and individuals. It causes negative effects on the health systems of countries, and has different effects on individuals, including physical, emotional, and financial strain. Many health systems in underdeveloped countries are not prepared to manage this burden; thus, cancer patients suffer from the absence of quality diagnosis and treatment. In countries where health systems are strong, the survival rates are improved [1,2,3,4]. 

Since 1951, the initial isolation of marine-derived sesquiterpenoid was reported by Takaoka and Ando [5]. An ever-increasing role in searching for novel bioactive secondary metabolites is assumed by marine natural products (MNPs) [6]. MNPs are characterized by their diversity of molecular weight, greater numbers of hydrophobic chains, and higher number of halogens, sulfur, and nitrogen atoms. Whereas, they have lower average number of oxygen atoms, and fewer aromatic rings [7].

The terrestrial natural products (TNPs) play a vital role in drug discovery, particularly cancer. This has become apparent through the production of molecular conformity of bioactive metabolites. An example of recently bioactive sesquiterpenes, trilobolide and thapsigargin belong to the guaianolide type and are closely related to each other. Thapsigargin displayed potent inhibition of sarco/endoplasmic reticulum calcium ATPase (SERCA), which has a role in the maintenance of Ca^2+^ ion homeostasis. Additionally, thapsigargin was entered in a clinical application. Moreover, trilobolide showed potent immunostimulatory activity, and thus, an application for a patent has been submitted. Series of trilobolide-steroids conjugates were evaluated for inhibition of sarco/endoplasmic reticulum Ca^2+−^ATPase (SERCA) and showed variable effects. [8,9,10,11]. 

As aforementioned, MNPs possess different and significant biological activities, including cytotoxic activity [12]. Moreover, MNPs play an important ecological role amongst marine organisms, especially invertebrates, which compete intensely for nutrients, light, and space [13]. In this context, invertebrates, such as the soft coral *Xenia* sp., are recognized as a source of secondary marine natural products such as terpenoids, including diterpenes and sesquiterpenes, capnellenes, and steroids [14]. The diversity of marine-derived sterols was predicted by Bergmann in 1962, who extensively reviewed sterols’ structures and distribution [15]. The position of unsaturation bonds and the poly hydroxylation were the characteristic features of marine steroids compared to terrestrial steroids. The uncommon structures of marine-derived sterols were reviewed by Schmitz; gorgosterol is an example of these uncommon structures [16]. After isolation of the rare gorgostane [17], which demonstrates unprecedented side chain of eleven carbon atoms rather than the frequently identified of 8 to 10 carbons [18], many gorgostane-type steroids have been isolated from different hosts, as 3β-,5α-,6β-,11α-,20β-pentahydroxygorgosterol, which was isolated from the *X. umbellata* soft coral [19]. 

In the current work, the soft coral *Xenia umbellata*, afforded a new xenicane diterpene, xeniolide O (**5**), and a new gorgostane steroid, gorgst-3β,5α,6β,11α,20(*S*)-pentol-3-monoacetate (**7**), together with five known isoprenoids; aromadendrene (**1**), palustrol (**2**), viridiflorol (**3**), xeniolide I (**4**), and 23,24-dimethylcholest-16-ene-3β,5α,6β,20(*R*)-tertrol 3-monoacetate (**6**) [20].

## 2. Results

### 2.1. Chemistry

**Figure 1:**
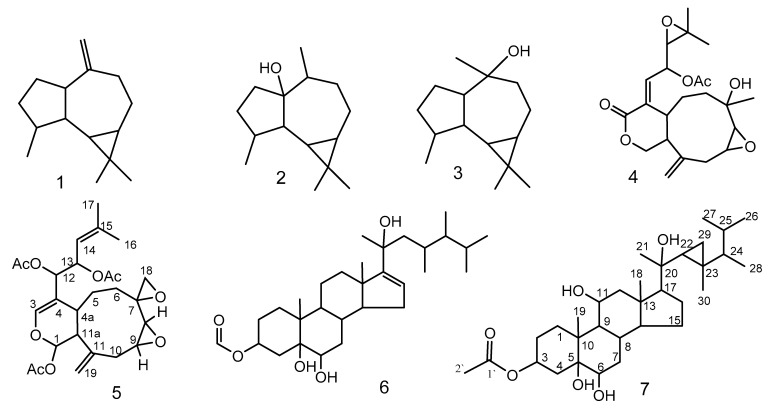
Compounds **1**–**7** isolated from *Xenia umbellata.*

The dried powder of the soft coral *Xenia umbellata* was extracted three times using equal volumes of MeOH and CH_2_Cl_2_, until exhaustion, and then concentrated under reduced pressure to yield an oily material. The concentrated organic extract was purified employing different chromatographic techniques to afford two new compounds, **5** and **7,** (Figure 1 and Table 1 and Table 2), along with five known compounds: **1**–**4** and **6**. 

Xeniolide O (**5**) was isolated as pale oily material (2.1 mg, 0.0014%); [α${]}_{D}^{22}$+ 37.0 (c 0.02, CHCl_3_); IR ʋ_max_ (film) cm^−1^: 2980, 2936, 2851, 1734, 1665, 1462, 1374, 1205 and 1152; HRESIMS *m*/*z* = 490.2197 [M]^+^ (Calculated *m*/*z*= 490.2203 for C_26_H_34_O_9_); ^1^H NMR (CDCl_3_, 850 MHz) and ^13^C NMR (CDCl_3_, 213 MHz) (Table 1 and Figure 1).

Gorgst-3β,5α,6β,11α,20(*S*)-pentol-3-monoacetate (**7**) was isolated as an oily material (0.8 mg, 0.00054%); [α${]}_{D}^{22}$54.0 (c 0.01, CHCl_3_); IR ʋ_max_ (neat) cm^−1^: 3426, 2925, 2850, 2193, 2016, 1714, 1460, 1379, 1266, 1029; ^1^H NMR (CDCl_3_, 850 MHz) and ^13^C NMR (CDCl_3_, 213 MHz) (Table 2); HRESIMS *m*/*z* = 534.3914 [M]^+^ (Calculated *m*/*z* = 534.3920 for C_32_H_54_O_6_).

### 2.2. Biological Activities

#### 2.2.1. Anti-Proliferative Activity

In vitro cytotoxicity of the isolated compounds (**1**–**7**) was evaluated, against MCF-7, HepG2, and HeLa, by using the sulphorhodamine B (SRB) assay in a concentration range of 0.01 to 1000 μg/mL. The results are shown in Table 3 and Figure S1a,b. For three decades, the sulforhodamine B (SRB) assay has remained one of the most widely used methods for antiproliferative evaluation. The assay was trusted based on the ability of SRB to bind to protein components of the cells that have been fixed to tissue- culture plates by trichloroacetic acid (TCA). SRB is a bright pink aminoxanthene dye with two sulfonic groups that bind to basic amino-acid residues under mildly acidic conditions and dissociate under basic conditions. As the binding of SRB is stoichiometric, the amount of dye extracted from stained cells is directly proportional to the cell mass. It is a sensitive method that can detect densities as low as 1000–2000 cells per well. The dose-response curves of the SRB assay created by GraphPad prism software follow the familiar symmetrical sigmoidal shape. This model fits a dose response curve to determine the IC_50_ of the drugs (the concentration that gives a response half-way between the baseline and maximum). The data represent mean ± SD (n = 3) [21].

#### 2.2.2. Apoptotic Effect

**Figure 2:**
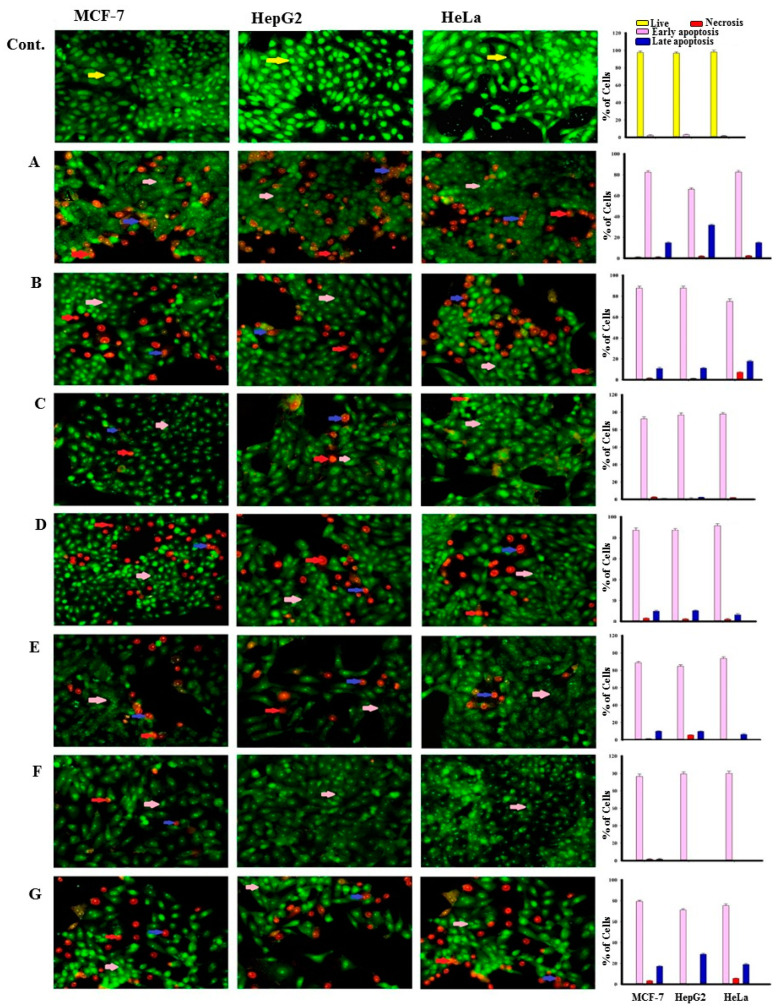
Morphological and nuclear changes using acridine orange (AO) and ethidium bromide (EB) staining, that were evaluated by the effect of compounds treatment on apoptosis of MCF-7, HepG2, and HeLa human tumor cells after 48 h treatment, induced various nuclear changes such as chromatin fragmentation and condensation and nuclei condensation at 200×. Yellow arrows indicate live cells, pink arrows indicate early apoptotic, red arrows indicate necrotic and blue arrows indicate late apoptotic cells. Cells were treated with IC_50_ of compounds **1** (**B**), **2** (**C**), **3** (**D**), **4** (**E**), **5** (**F**), 6 (**E**), and **7** (**G**) 48 h. The control (**A**) was similarly processed. The (**A**–**G**) were stained with acridine orange-ethidium bromide. (**Top**): The control (**A**) was processed in the same manner as the compounds **1**–**7** for 48 h.

After staining with AO/EtBr, the cells appeared in form of four colors as follows: living cells (normal green nucleus), early apoptotic (bright green nucleus with fragmented chromatin), late apoptotic (orange-stained nuclei with chromatin condensation or fragmentation), and necrotic cells (uniformly orange-stained cell nuclei). In AO/EtBr dual staining, the cells were uniformly stained green with normal, round, intact nuclei and cytoplasm that indicates the viability of the cell control (Figure 2). 

## 3. Discussion

### 3.1. Chemistry

The soft coral genus *Xenia* has been recognized as an important source of terpenoidal derivatives. It produced around 200 terpenoid derivatives including diterpenoids and steroids, which are biosynthesized by four and six isoprene units, respectively. 

Genus *Xenia* produces a characteristic type of diterpenes (Xenicane). This family has a variety of functional groups and structural modifications as acetylation, epoxidation, hydroxylation, methylation, and oxygenation, giving them additional carbon and oxygen atoms in their molecular formula. Xenicane diterpenes have a cyclononane skeleton and can be classified into several types, including xenicins [22], xeniolides [23], and xeniaphyllanes [24]. Subsequently, more groups were categorized, such as floridicins [25], xeniaethers [26], and azamilides [27]. The majority of these compounds displayed anticancer activities. There were few steroids reported from this genus. The current results showed two steroids; one of them is new and luckily showed antiproliferative activity. This finding is supported by the previously published four new steroids, xeniasterols A–D from Japanese *Xenia* sp. [28].

**Figure 3:**
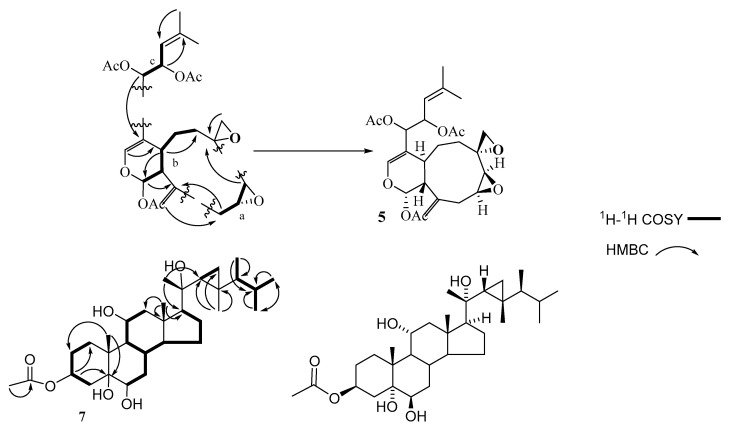
Selected COSY and HMBC correlations of **5** and **7**.

Compound **5** was isolated as optically active yellowish oily material. The molecular formula of C_26_H_34_O_9_, was established based on HREIMS and one- and two-dimensional NMR spectroscopy. In the IR spectrum, the presence of acetate function and carbon-carbon double bond were indicated by the absorption bands at 1734 and 1655 cm^−1^, respectively. ^13^C and DEPT NMR spectra categorized the carbon atoms into five methyl (δ_C_ 18.6, 3 × 21.2, and 25.9 ppm), five methylene (26.2, 27.4, 33.6, 50.7, and 114.0 ppm), nine methine (140.2, 119.3, 91.2, 74.4, 70.1, 57.7, 56.2, 50.7, and 29.6 ppm), and seven quaternary carbons (170.0 *×* 2, 169.5, 141.9, 140.5, 140.2, 110.8, and 53.5) (Supplementary Materials Figure S2). ^1^H-NMR evidenced the presence of two olefinic protons resonating at δ_H_ 6.40 and 5.11 ppm, two vinyl methyl functions at δ_H_ 1.73 and 1.71, two exo-cyclic methylene protons at 5.07 and 5.06, and three acetates (2.09/21.2 and 169.5; and two 2.01/21.2 and 170.0 ppm). ^1^H and ^13^C-NMR and HSQC experiments displayed signals assigned to five sp^3^ oxygenated methines, an oxygenated methylene (δ_H_/ δ_C_ 2.85 and 2.60/ 50.7), an oxygenated quaternary carbon (δ_C_ 53.5), and an sp^2^ oxygenated methine at 6.40/140.2. Interestingly, the chemical structure contained an anomeric function, absorbing at 6.33/91.2 ppm. The molecular formula of compound **5** displayed ten unsaturations, of which three carbonyl esters, two trisubstituted carbon-carbon double bonds, and a vinyl function were gleaned from the NMR spectroscopic data. The remaining four sites of unsaturation imply a tetracyclic structure of compound **5**. The data gleaned from the ^1^H-^1^H COSY spectrum indicated the presence of three proton sequences: (a) The proton resonating at δ_H_ 3.22 (H-8) is correlated with that at 3.05 (H-9), which in turn is correlated to the methylene protons (H-10) resonating at 2.73 and 2.65 (Figure 3), (b) the second sequence is extended from the anomeric proton which is resonating at δ_H_ 6.33 (H-1) to the methylene protons H-6 (summarizes as CH(1)-CH (11a)-CH (4a)-CH_2_ (5)-CH_2_ (6)), and (c) the third fragment linked the methine proton H-12 to H-13 and H-13 to H-14 (Table 1). The HMBC spectrum established the presence of the following moieties: a nine-membered ring, which is a common finding in the alcyonaceans of the genus *Xenia*, a dihydropyran ring fused to the nine-membered carbon skeleton deduced from the correlation of H-1 (the anomeric proton) with C-11a, 11, 4a and C-3 (over oxygen atom) and the correlation of H-4a with C-4, C-3, and C-12, a two-methines containing oxirane and a methylene containing oxirane rings (Figure 3 and Table 1), the exocyclic double bond was located at C-11 based on the correlation of H-19 with C-11, C-10, and C-11a, and finally the forked-tail side chain was deduced to be a derivative of 2-methyl-2-pentene. The gross structure of **5** was deduced as shown in Figure 1. The stereochemistry of compound **5** was gleaned from the coupling constant (*J*), from the NOESY spectrum, and by comparison with the published data [29]. The trans-fusion of the dihydropyran ring with the nine-membered carbon skeleton was deduced from the absence of cross-peak between H-4a and H-11a. The small coupling constant exerted by H-1 (*J* = 2.5 Hz) and the broad singlet multiplicity of the H-11a signal implies equatorial orientations of H-1 and H-11a. The cross-peaks observed between H-1, H-18, and H-11a evidence their co-facial β-orientation [29]. Compound **5** is a member of a famous diterpenoid class with a xenicane carbon skeleton, xenicanes are major metabolites of the soft corals of the genus *Xenia* [19,22,28,29]. The name xeniolide O was assigned to compound **5**. 

Compound **7** was isolated as an oily material. The molecular formula of C_32_H_54_O_6_ was established based on HREIMS, IR, 1D, and 2D-dimensional NMR spectroscopy. In the IR spectrum, the presence of hydroxyl group stretching, carbonyl group, methylene scissoring bending, gem-dimethyl, C-O-H stretching, and cyclopropyl ring, were indicated by the absorption bands at 3426, 1714, 1460, 1379, 1266, and 1029 cm^-1^, respectively. 

^13^C- and DEPT NMR spectra categorized the carbon atoms into 5 quaternary, 8 methylene, 11 methine, and 8 methyl carbons (Table 2). HSQC spectrum accounted for all protons but three, which were assigned to three hydroxyl functions. ^1^H- and ^13^C- NMR displayed the presence of three oxygenated methines, an oxygenated quaternary carbon, and a downfield sp^3^ methine proton signal resonating at δ_H_ 5.13 ppm. Moreover, the spectra displayed the presence signals attributed to five quaternary methyls and three tertiary methyls (acetate function (δ_H_ 2.04; δ_C_ 170.8 and 21.4)).

In the conventional one-dimensional ^1^H-NMR spectrum at the downfield region, in addition to the two-dimensional correlation-spectroscopy COSY (Figure 3), a dddd (doublet of doublets of doublets of doublets) at 5.13 ppm of H_1_-3 resulted from the two di-axial 11.1 and 11.1 Hz and two axial-equatorial 5.1 and 5.1 Hz couplings (Figure S3). This deduced the axial position of this proton (H_1_-3) among two methylene groups. The chemical shifts values of H_1_-3 at 5.13 ppm indicating the through space effect of the carbonyl group at H_1_-3 that caused additional shifting to the downfield region, whereas a ddd (doublet of doublets of doublets) of H_1_-11 at 3.88 ppm caused by the two di-axial 10.2 and 10.2 Hz and an axial-equatorial 5.1 Hz couplings suggest the axial position of H_1_-11 between a methine and a methylene groups. The equatorial position of H_1_-6 at 3.52 ppm showed a broad signal which is suggested to be caused by the vicinal couplings of H_2_-7 based on COSY. 

In the up-field region of the ^1^H-NMR spectrum, four characteristic signals of gorgostane side chain have been observed: a doubled triplet signal due to methine proton resonating at 0.15 ppm (H_1_-22) with coupling constant values of 8.5 and 6.0 Hz, and a doubled quartet signal due to CH at 0.23 ppm (H_1_-24) with coupling constant values of 13.6 and 6.8 Hz, along with signal due to methylene protons resonating at 0.46 ppm appeared as a doublet of doublets with coupling constant values of 8.5 and 4.3 Hz, and at -0.13 appeared as a doublet of doublets with coupling constant values of 6.0 and 4.3 Hz [19]. The large coupling values in the side chain were reported due to the steric substitution effect [30]. Small isolated geminal coupling values of 4.3 and 4.3 Hz of H_2_-29 at −0.13 and 0.45 ppm are related to the unusual angle of the C-C-C bond in the cyclopropyl ring that decreased from 180° to be 60° due to the strain, and separated the two protons at C-29 [31], whereas 8.5 and 6.0 Hz of these two chemical shifts, respectively, represented the vicinal coupling of 2H-29 to H-22. 

Through-bond correlations of HMBC support the suggested positions of the quaternary carbons and the methyl groups, especially at the side chain, as the correlations of H_3_-21 with (C-17, C-20 and C-22), of H_3_-30 with (C-29 and C-22), of H_3_-28 with (C-23, C-24, and C-25), and of H_3_-26 with (C-24, C-25, and C-27), (Table 2). Therefore, the side chain is identical to that of gorgostane with one single difference, which is the hydroxylation of C-20.

Biogenetically, the two angular methyls Me-18 and Me-19 and the side chain at C-17 are all β-oriented [32]. Therefore, the α5 and α14 configuration and the four trans-fused rings are determined by the NOESY cross-peaks, that showed the through-space correlations of the βH_3_-19 with βH_2_-4a, of αH_2_-1a with αH_1_-3, and of αH_1_-14 with αH_1_-1. Meanwhile, there were no such interactions between the βH_3_-19 and H_2_-1a or αH_1_-17 and βH_2_-12a. 

In the side chain, through-space correlations of NOESY were observed of βH_3_-19 with H_3_-21, of H_3_-21 with H_2_-12a, H1-22, H_2_-29b, and H_3_-28, and of H_2_-29b with H_3_-30. Therefore, the orientation of these protons is beta. The configuration at the chiral centers in this chain as the 20*S*, 22*R*, 23*R*, and 24*S* was determined based on these correlations (Table 2). Therefore, compound **7** can be identified as gorgst-3β,5α,6β,11α,20(*S*)-pentol-3-monoacetate (Figure 3).

### 3.2. Biological Activities

The current manuscript is interested in the evaluation of the antiproliferative effect of the isolated metabolites (**1**–**7**) against three types of cancer cells; two of them are female (MCF-7 and HeLa), and the third is a common cancer in both genders (HepG2). The sulphorhodamine B (SRB) assay was selected because of its sensitivity. It estimates the cell density by measuring the cellular protein content. 

Compounds **1**–**7** displayed a cytotoxic effect against MCF-7 with IC_50_ values in the range of 1.5 ± 0.1 and 23.2 ± 1.5 μg/mL. They showed a cytotoxic effect against HepG2 with IC_50_ values in the range of 1.8 ± 0.1 to 30.6 ± 1.1 μg/mL. Also, they displayed cytotoxicity against HeLa with IC_50_ values in the range of 0.9 ± 0.05 to 12.8 ± 0.5 μg/mL. 

Compounds **1**, **3**, and **6** showed potent cytotoxic effects against MCF-7, HepG2, and HeLa with IC_50_ values in the range of 0.9 ± 0.05 to 3.1 ± 0.1, and most of them are more potent than the doxorubicin. 

Compounds **4**, **5**, and **7** displayed a modest antiproliferative activity on MCF-7, HepG2, and HeLa cells with IC_50_ values in the range of 18.2 ± 1to 30.6 ± 1.1, while the same compounds have significant activity towards HeLa cells with IC_50_ values 7.8 ± 0.5, 6.7 ± 1and 11.5 ± 2.2 μg/mL. Compound **2** has a promising toxicity on MCF-7 and HeLa cells with IC_50s_ 7.5 ± 0.4 and 12.8 ± 0.5 2 μg/mL, respectively, whereas the same compound has a moderate effect on HepG2 cells with IC_50_ value 21.8 ± 0.2 μg/mL. 

Conclusively, the isolated sesquiterpenes (**1**–**3**) displayed a more potent cytotoxic effect than the isolated steroids (**6** and **7**), which in turn displayed more potent cytotoxic activities than the diterpenes (**4** and **5**).

Compounds **1**–**3**, in general, displayed more potent effects than other compounds. It was reported that the sesquiterpenes are well known for their cytotoxicity. Although they have the same skeleton, compounds **1** and **3** displayed more or less similar effects, whereas compound **2** showed less activity but was still potent. The difference between **2** and **3** is the position of the hydroxyl group, which could play a role in the activity. This finding agrees with the literature. The sesquiterpenes are a large group of secondary metabolites, which consist of three isoprene building units. These metabolites were isolated from different natural sources including terrestrial and marine. They are characteristically associated with plant defense mechanisms. Over the last two decades, these substances were recognized for their bio-effects, including hyperglycemia, hyperlipidemia, cardiovascular complications, neural disorders, diabetes, and cancer. It was reported that they have antiproliferative effects against the breast, colon, bladder, pancreatic, prostate, cervix, brain, liver, blood, ovarium, bone, endometrium, oral, lung, eye, stomach, and kidneys. Our finding coincides with the previous reports on these classes of natural products [33]. 

Although compounds **6** and **7** are steroidal derivatives, compound **6** displayed a more potent effect than **7.** The differences between the two structures are the side chain and the presence of the hydroxyl group in position 11 in **6**, which do not exist in **7**, and the presence of a double bond (C_16_ = C_17_). These may decrease the activity of compound **7**. This finding is in a good agreement with the cytotoxic effect of the steroidal derivatives [34].

Highly early apoptotic cell death was observed in all types of treated tumor cells. Regularly stained green with normal, round, intact nuclei and cytoplasm indicates the viability of the normal cell (control). Whereas a high rate of cell death was observed in early apoptosis with compound **6**, the percentage was low after treatment with compound **4** against cancer cells. In addition, compound **5** induces a high percentage of necrosis pathway towards cells, compound **7** induces necrosis in HeLa cells, while compound **6** does not cause the necrosis death of both cancer cells (HepG2 and HeLa) after treatment (Figure 3). Late apoptosis was shown at a high rate after treatment with compound **7** on all cancer cells and compound **1** induces more late apoptosis in HepG2 than in other cancer cells.

## 4. Material and Methods

### 4.1. General 

The NMR deuterated solvent used in this study was chloroform-d, 99.8 atom % D. Silica Gel Sorbent (70-230 Mesh, Grade 60; 63 to 200 µm) was used in the open column chromatography. In the analysis of TLC, Aluminum SIL G plates with fluorescent indicator UV254 (0.25 mm) (20 × 20 cm) were used. KRUSS P 8000 polarimeter, KRUSS OPTRONIC (Hamburg, Germany) was used to determine the optical rotations. NMR spectra were recorded for 1D and 2D on Bruker 850 MHz 1H-NMR, 212 MHz ^13^C-NMR, and DEPT spectrometer at the Department of Chemistry, Faculty of Science, King Abdulaziz University Jeddah, Saudi Arabia. 

### 4.2. Animal Material

Soft coral Lamarck *Xenia umbellata* (Order Alcyonacea, Family Alcyoniidae) was collected at a depth of 15–20 m by scuba diving, from Jeddah, the Red Sea coast, Saudi Arabia (21°29′31″ N 39°11′24″ E) in August 2019. This species was identified by Mohsen El-Sherbiny, Marine Biology Department, Faculty of Marine Sciences, King Abdulaziz University (KAU) Jeddah, Saudi Arabia.

### 4.3. Extraction and Isolation

Approximately 150.0 g of the dried *X. umbellata* was extracted three times using equal amounts of MeOH and CH_2_Cl_2_ at ambient temperature, followed by the filtration and the evaporation of the combined extracts by rotary evaporator to yield 21.4 g of oily material. The degradation was minimized by storing the residue at low temperatures (4–5 °C).

The oily residue (21.4 g) was homogenized with an amount of silica gel then chromatographed using an open column (100 × 3.2 cm) over 500 g of silica gel (70-230 Mesh, Grade 60). The initial eluent was *n*-hexane, then the polarity increased gradually using the CH_2_Cl_2_. The volume of collected fractions was 50 mL for each. The TLC technique, along with both UV light and spray reagents, was used to trace the fractionation. Further purification to isolate a single compound was achieved by preparative TLC (PTLC). The fraction eluted with *n*-hexane (14.1 mg) was re-purified by PTLC. The violet band (with spray reagent) at R_f_ = 0.90 yielded a pale-yellow oil (**1**, 4.1 mg). The fraction eluted by *n*-hexane- CH_2_Cl_2_ (80:20) was collected and subjected to PTLC using the solvent system *n*-hexane-Ethyl acetate (80:20). Two brown bands with spray reagents were obtained; the highest at R_f_ 0.70 gave a colorless oil (**2**, 2.2 mg) and the lowest R_f_ 0.40 gave a colorless oil (**3**, 1.0 mg). The fraction, which eluted with *n*-hexane–CH_2_Cl_2_ (65:35) was collected and re-purified by PTLC using *n*-hexane-EtOAc (70:30) to yield two bands: a dark blue band by the spray reagent with an R_f_ value of 0.7 (**5**, 2.1 mg) and a brown band with an R_f_ value of 0.35 (**6**, 0.7 mg). The fraction eluted by *n*-hexane-CH_2_Cl_2_ (60:40) afforded compound (**7**, 0.8 mg), which was re-purified by PTLC using n-hexane-EtOAc (7:3) and showed a brown band by the spray reagent with an R_f_ value of 0.31. The fraction eluted by *n*-hexane-CH_2_Cl_2_ (40:60) afforded compound **4**, which was re-purified by PTLC using *n*-hexane-EtOAc (50:50) and showed a blue band by the spray reagent with an R_f_ value of 0.15 (**4** and 3.0 mg). 

### 4.4. Biological Activities 

#### 4.4.1. Antiproliferative Activity

##### Cell Culture

MCF-7, HepG2, and HeLa cells were obtained from the American type culture collection (ATCC). Cells were maintained in RPMI-1640 supplemented with (100 μg/mL); penicillin (100 units/mL) and heat-inactivated fetal bovine serum (10% *v*/*v*) in a humidified, 5% (*v*/*v*) CO_2_ atmosphere at 37° [35].

##### Sulphorhodamine B Assay (SRB)

The cytotoxicity of the newly isolated compounds was evaluated against MCF-7, HepG2, and HeLa cells using a Sulphorhodamine B assay (SRB). Ninety percent of confluency growing cells were trypsinized and cultured in a 96 well tissue culture plate (3000 cells/well) for 24 h before treatment with the newly isolated compounds. Cells were exposed to the six different concentrations of each compound (0.01, 0.1, 1, 10, and 1000 µg); untreated cells (control) were added. The cells were incubated with the concentrations for 72 h and subsequently fixed with TCA (10% *w*/*v*) for 1 h at 4 °C. After several washes, cells were stained with 0.4% (*w*/*v*) SRB solution for 10 min in a dark place. Excess stain was washed with 1% (*v*/*v*) glacial acetic acid. After drying overnight, the SRB-stained cells were dissolved with Tris-HCl and the color intensity was measured in a microplate reader at 540 nm. The relation between the viability percentage of each tumor cell line and compound concentrations were analyzed to get the IC_50_ (dose of the drug which reduces survival to 50%) using SigmaPlot 12.0 software [35].

##### Acridine Orange/Ethidium Bromide Staining for Detection of Apoptosis

DNA binding dyes Acridine orange (AO) and Ethidium bromide (EtBr) have been used for the morphological detection of viable, apoptotic, and necrotic cells. AO is taken up by both non-viable and viable cells that emit green fluorescence when intercalated into DNA. EtBr is taken up only by nonviable cells, whereas it is excluded by viable cells and emits red fluorescence by intercalation into DNA. Cells were seeded on a cover slide inside a six-well plate. Cells were incubated in a CO_2_ incubator with 37 °C temperature and 5% CO_2_ for 24 h then treated with IC_50s_ concentration of compounds **1**–**7** and incubated for 48 h. Cells were washed with cold PBS 1x three times. Cells were stained with a mixture of Acridine Orange 100μg/mL /Ethidium Bromide (AO/EB) 100 μg/mL in PBS 1x with 10% FBS on each well and then incubated for 5 min in RT. The cover slides with cultured stained cells were transferred immediately to new slides and the cells were ready to be visualized by the blue filter of the fluorescence microscope [36]. 

### 4.5. Statistical Analysis 

Data are presented as mean SD unless otherwise indicated. Statistical significance was acceptable to a level of *p* < 0.05. All statistical analyses were performed using GraphPad InStat software, version 3.05 (GraphPad Software, La Jolla, CA, USA). Graphs were plotted using GraphPad Prism software, version 6.00 (GraphPad Software, La Jolla, CA, USA).

## 5. Conclusions

Seven isoprenoids were isolated from the alcyonacean *Xenia umbellata,* including a new gorgostane derivative gorgst-3β,5α,6β,11α,20(*S*)-pentol-3-monoacetate, a new xenicane diterpene, xeniolide O, along with three known sesquiterpenes, a known isodinosterol derivative, and a known diterpenoid. The two steroidal derivatives displayed higher potent cytotoxic activities than the two diterpene derivatives. Compounds **1**–**7** displayed a cytotoxic effect against MCF-7, HepG2, and HeLa with IC_50_ values < 23.2 ± 1.5, 30.6 ± 1.1, and 12.8 ± 0.5 μg/mL, respectively. Compound 3 showed potent cytotoxic effects against MCF-7, HepG2, and HeLa with IC_50_ values < 3.1 ± 0.10 μg/mL, respectively. Compounds 2, 5, and 7 displayed a cytotoxicity effect against Hela cells with IC_50_ values < 12.8 ± 0.50 μg/mL, respectively. The late apoptotic cells are highly present in HepG2 cells. The two steroids induced a high percentage of necrosis towards HepG2 and HeLa cells. The late apoptosis was recorded as a high rate after treatment with the new steroid on all cancer cells. The results are highly interesting; thus, a deep pharmacological mechanism is required.

## Figures and Tables

**Table 1:** ^1^H and ^13^C NMR spectral data of compound **5**^a^.

Carbon No.	δ_C_^b^	δ_H_ (*J* in Hz) ^c^
1	91.2 (CH)	6.33 (d, 2.5)
3	140.2 (CH)	6.40 (s)
4	110.8 (C)	-
4a	29.6 (CH)	2.92 (m)
5	27.4 (CH_2_)	1.92 (m) 1.72 (m)
6	26.2 (CH_2_)	1.60 (m) 1.45 (m)
7	53.5 (C)	-
8	56.2 (CH)	3.22 (d, 4.3)
9	57.7 (CH)	3.05 (ddd, 11.0, 7.7, 4.3)
10	33.6 (CH_2_)	2.73 (dd, 14.5, 4.3) 2.65 (ddd, 14.5, 11.0, 7.7)
11	141.9 (C)	-
11a	50.7 (CH)	2.87 (brs)
12	74.4 (CH)	5.33 (d, 6.0)
13	74.1 (CH)	5.77 (dd, 9.5, 6.0)
14	119.3 (CH)	5.11 (dt, 9.5, 6.0)
15	140.5 (C)	-
16	25.9 (CH_3_)	1.71 (brs)
17	18.6 (CH_3_)	1.73 (brs)
18	50.7 (CH_2_)	2.85 (d, 6.0) 2.60 (d, 6.0)
19	114.0 (CH_2_)	5.07 (brs) 5.06 (brs)
Ac-1	169.5, 21.2	2.09 (s)
Ac-12	170.0, 21.2	2.01 (s)
Ac-13	170.0, 21.2	2.01 (s)

^a^ All assignments are based on both 1D and 2D experiments (HMBC, HSQC, COSY). ^b^ Implied multiplicities were made by DEPT (C = s, CH = d, CH_2_ = t). ^c^ J in Hz.

**Table 2:** ^1^H and ^13^C NMR spectral data of compound **7**^a^.

C No.	δ_C_^b^		δ_H_ (*J* in Hz) ^c^	C No.	δ_C_^b^		δ_H_ (*J* in Hz) ^c^
1	26.9 (CH_2_)	H_2_-1a	1.85 (m)	16	28.2 (CH_2_)	H_2_-16a	2.05 (m)
		H_2_-1b	1.66 (m)			H_2_-16b	1.32 (m)
2	34.0 (CH_2_)	H_2_-2a	1.83 (m)	17	57.8 (CH)	H_1_-17	1.29 (m)
		H_2_-2b	2.12 (m)	18	13.0 (CH_3_)	H_3_-18	0.67 (s)
3	70.8 (CH)	H_1_-3	5.13 (dddd, 11.1, 11.1, 5.1, 5.1)	19	16.9 (CH_3_)	H_3_-19	1.31 (s)
4	37.5 (CH_2_)	H_2_-4a	2.18 (m)	20	76.3 (C)	-	-
		H_2_-4b	1.60 (m)	21	21.1 (CH_3_)	H_3_-21	1.01 (s)
5	76.8 (C)	-		22	31.9 (CH)	H_1_-22	0.15 (td, 8.5, 6.0)
6	76.2 (CH)	H_1_-6	3.52 (brt, 2.5)	23	25.8 (C)	-	-
7	34.5 (CH_2_)	H_2_-7a	1.77 (m)	24	50.7 (CH)	H_1_-24	0.23 (qd, 13.6, 6.8)
		H_2_-7b	1.56 (m)	25	32.0 (CH)	H_1_-25	1.56 (m)
8	29.0 (CH)	H_1_-8	1.75 (m)	26	22.17 (CH_3_)	H_3_-26	0.95 (d, 6.8)
9	52.7 (CH)	H_1_-9	1.38 (m)	27	21.5 (CH_3_)	H_3_-27	0.85 (d, 6.8)
10	39.9 (C)	-		28	15.5 (CH_3_)	H_3_-28	0.93 (d, 6.8)
11	68.6 (CH)	H_1_-11	3.88 (ddd, 10.2, 10.2, 5.1)	29	21.3 (CH_2_)	H_2_-29a	0.46 (dd, 8.5, 4.3)
12	51.9 (CH_2_)	H_2_-12a	2.35 (dd, 11.1, 5.1)			H_2_-29b	−0.13 (dd, 6.0, 4.3)
		H_2_-12b	1.19 (m)	30	14.2 (CH_3_)	H_3_-30	0.89 (s)
13	43.6 (C)	-		1′	170.8 (C)	-	-
14	54.8 (CH)	H_1_-14	1.19 (m)	2′	21.4 (CH_3_)	H_3_-2′	2.03 (s)
15	24.4 (CH_2_)	H_2_-15a	1.59 (m)				
		H_2_-15b	1.05 (m)				

^a^ All assignments were based on both 1D and 2D experiments (HMBC, HSQC, COSY). ^b^ Implied multiplicities were made by DEPT (C=s, CH=d, CH_2_=t). ^c^ J in Hz.

**Table 3:** Summary of cytotoxic effects (IC_50_, μg/mL) in three cancer cell lines of the isolated compounds (**1**–**7**). SRB assay was used and the cells were treated with different concentrations of different isolated materials for 72 h. Values in the table represent the means of IC_50_ from 3 independent experiments, with SE ranging from 19% to 33% of the average values. The tested cell lines of human origin were: MCF-7 (breast adenocarcinoma), HepG2 (hepatocellular carcinoma), and HeLa (cervix adenocarcinoma).

Compound Number	IC_50_ (μg/mL)		
	MCF-7	HepG2	Hela
**1**	1.7 ± 0.20	2.3 ± 0.14	1.1 ± 0.05
**2**	7.5 ± 0.40	21.8 ± 0.20	12.8 ± 0.50
**3**	2.4 ± 0.20	3.1 ± 0.10	0.9 ± 0.05
**4**	18.2 ± 1.00	30.6 ± 1.10	7.8 ± 0.50
**5**	23.2 ± 1.50	21.2 ± 1.70	6.7± 1.00
**6**	1.5 ± 0.10	1.8 ± 0.10	1.2 ± 0.20
**7**	19.1 ± 0.50	18 ± 0.60	11.5 ± 2.20
Doxorubicin	1.05 ± 0.02	0.95 ± 0.01	1.65 ± 0.15

## Data Availability

All data are available up on request from the authors.

## Supplementary Materials

The following are available online, Figure S1a: The concentration response curves of the compounds **1**–**7** against MCF-7, HepG2 and Hela human cells, Figure S1b: Apoptosis effects of compounds **1**–**7** on MCF-7, HepG2 and HeLa, Figure S2a–z: NMR spectra of compound 5, Figure S3a–z: NMR spectra of compound **7**.

## Sample Availability

Samples of the compounds were consumed during bioassays.

## Abbreviations

^1^H-NMR	Proton Nuclear Magnetic Resonance
^13^C-NMR	Carbon-13 Nuclear Magnetic Resonance
1D and 2D NMR COSY	One and two-dimensional Nuclear Magnetic Resonance ¹H-¹H Correlation Spectroscopy
DEPT	Heteronuclear Multiple Bond Correlation
HMBC	Distortionless Enhancement by polarization transefer
HSQC	Heteronuclear Single Quantum Coherence
brt	Broad triplet
d	Doublet
δ	Chemical shift
*J*	Nuclear spin-spin coupling constant
dd	Doublet of doublet
HeLa	Cervix adenocarcinoma
HepG2	Hepatocellular carcinoma
MCF-7	Human breast cancer
IC_50_	Half maximal inhibitory concentration
SD	Standard Deviation
CDCl_3_	Deuterated chloroform
CH_2_Cl_2_	Dichloromethane
MeOH	Methanol
TLC	Thin Layer Chromatography
PTLC	Preparative Thin Layer Chromatography
