# Supplementary material for: Antiproliferative Isoprenoid Derivatives from the Red Sea Alcyonacean Xenia umbellata
Source: Molecules. 2021 Mar 1;26(5):1311. doi: 10.3390/molecules26051311 (PMC7957567; doi:10.3390/molecules26051311)
Supplement: Supplementary file 1 [file molecules-26-01311-s001.pdf]

# SUPPLEMENTARY MATERIAL

# Antiproliferative isoprenoid derivatives from the Red Sea Alcyonacean *Xenia umbellata*

**Hanan I. Althagbi,[1,2] Fetri Budiyanto[3], Ahmed Abdel-Lateff,[4, 5] Khalid O. Al-Footy,[2] Nahed O. Bawakid[2], Mohamed A. Ghandourah[3], Mohammad Y. Alfaifi,[6] Serag Eldin I. Elbehairi,[6] and [7] Walied M. Alarif [3,*]**

[1] Department of Chemistry, Faculty of Science, University of Jeddah, P.O. Box 13151, Jeddah 21493, Saudi Arabia; halthagbi@uj.edu.sa.

[2.] Department of Chemistry, Faculty of Science, King Abdulaziz University, P.O. Box 80203, Jeddah 21589, Saudi Arabia; kalfooti@kau.edu.sa (K.O.A.), nbawaked@kau.edu.sa (N.O.B.)

[3] Department of Marine Chemistry, Faculty of Marine Sciences, King Abdulaziz University, P.O. Box 80207, Jeddah 21589, Saudi Arabia; welaref@kau.edu.sa (W.M.A.); fitri.budiyanto@gmail.com (F.B.); mghandourah@kau.edu.sa (M.A.G.).

[4.] Department of Natural Products and Alternative Medicine, Faculty of Pharmacy, King Abdulaziz University, P.O. Box 80260, Jeddah 21589, Saudi Arabia; ahmedabdellateff@gmail.com.

[5.] Department of Pharmacognosy, Faculty of Pharmacy, Minia University, Minia 61519, Egypt.

[6] Department of Biology, Faculty of Science, King Khalid University, Abha 9004, Saudi Arabia; alfaifi@kku.edu.sa (M.Y.A.); serag@kku.edu.sa (S.E.I.E.).

[7] Cell Culture Laboratory, Egyptian Organization for Biological Products and Vaccines, VACSERA Holding Company, Giza 22311, Egypt.

# Antiproliferative isoprenoid derivatives from the Red Sea Alcyonacean *Xenia umbellata*

**Abstract:** From the soft coral *Xenia umbellata*, seven isoprenoids were isolated, including a new xenicane diterpene, xeniolide O (**5**) and a new gorgostane derivative gorgst-3β,5α,6β,11α,20(*S*)-pentol-3-monoacetate (**7**), along with three known sesquiterpenes (**1**-**3**), a known diterpene (**4**) and a known steroid (**6**). The extensive analyses of the NMR, IR, and MS spectral data led to the determination of the chemical structures. Compounds **1**-**7** displayed cytotoxic effect against Breast adenocarcinoma (MCF-7), Hepatocellular carcinoma (HepG2) and cervix adenocarcinoma (Hela) with $IC_{50}$ values ranging between 1.5 ± 0.1-23.2 ± 1.5; 1.8 ± 0.1-30.6 ± 1.1 and 0.9 ± 0.05-12.8 ± 0.5 µg/mL, respectively. Compound **3** showed potent cytotoxic effects against MCF-7, HepG2 and Hela with $IC_{50}$ values = 2.4 ± 0.20, 3.1 ± 0.10 and 0.9± 0.05 µg/mL, respectively. Compounds **2**, **5**, and **7** displayed cytotoxicity against Hela cells with $IC_{50}$ values = 12.8 ± 0.50, 6.7± 1.00 and 11.5 ± 2.20 µg/mL, respectively. Two DNA binding dyes, Acridine orange (AO) and Ethidium bromide (EtBr) have been used for the detection of viable, apoptotic, and necrotic cells. The early apoptotic cell death was observed in all types of treated tumor cells. The late apoptotic cells are highly present in HepG2 cells. Compounds **5** and **7** induced a high percentage of necrosis towards HepG2 and HeLa cells. The late apoptosis was recorded as a high rate after treatment with **7** on all cancer cells.

**Keywords:** Red Sea; Alcyonacea; Xenia; Steroids; Diterpene; Xenican; Cytotoxicity; Apoptosis

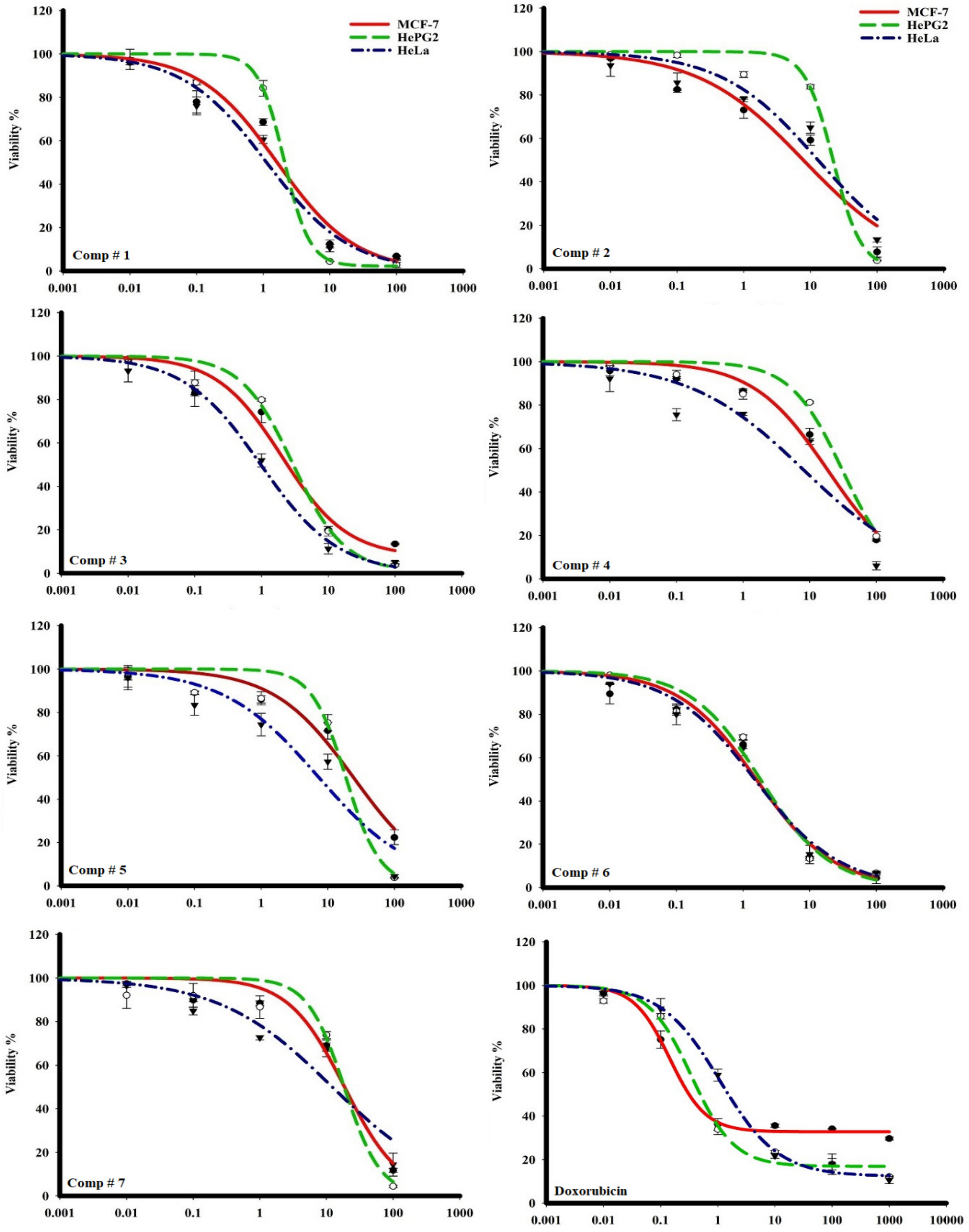


**Figure S1a.** The concentration response curves of the compounds **1**-**7** against MCF-7, HepG2 and Hela human cells.

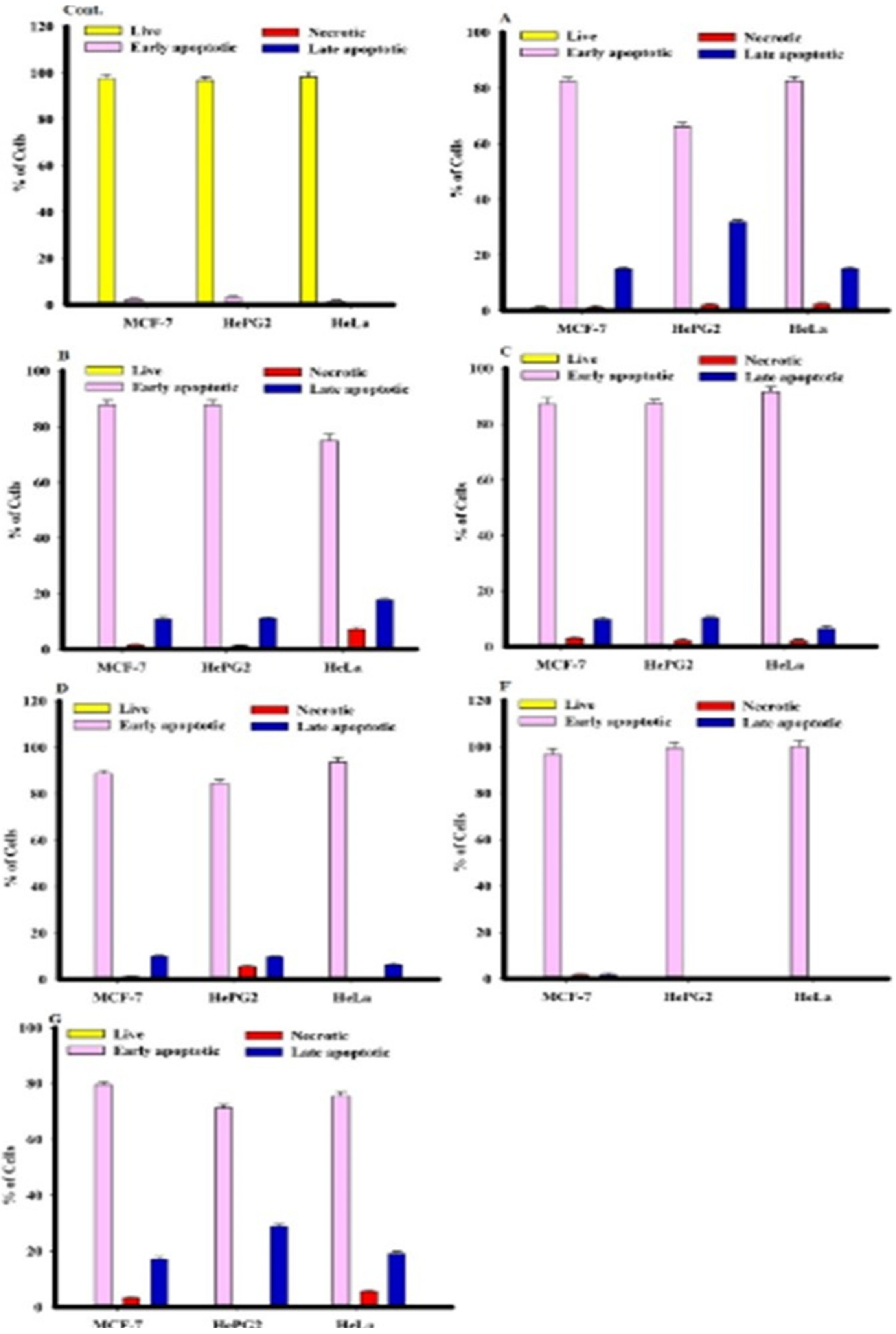


**Figure S1b. Apoptosis effects of compounds 1-7 on MCF-7, HepG2 and HeLa**

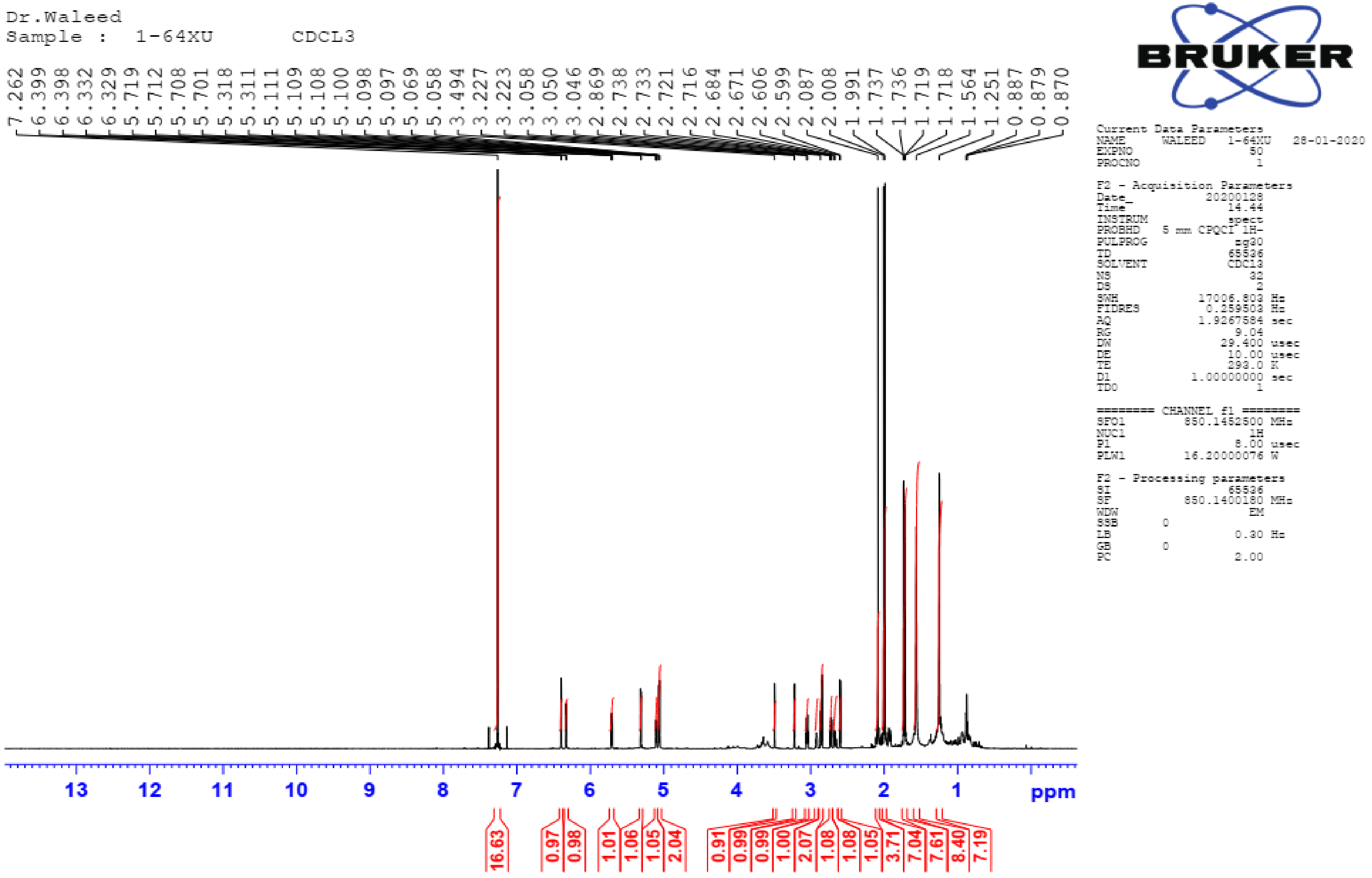


**Figure S2a.** $^{1}H$ NMR spectrum of compound **5**

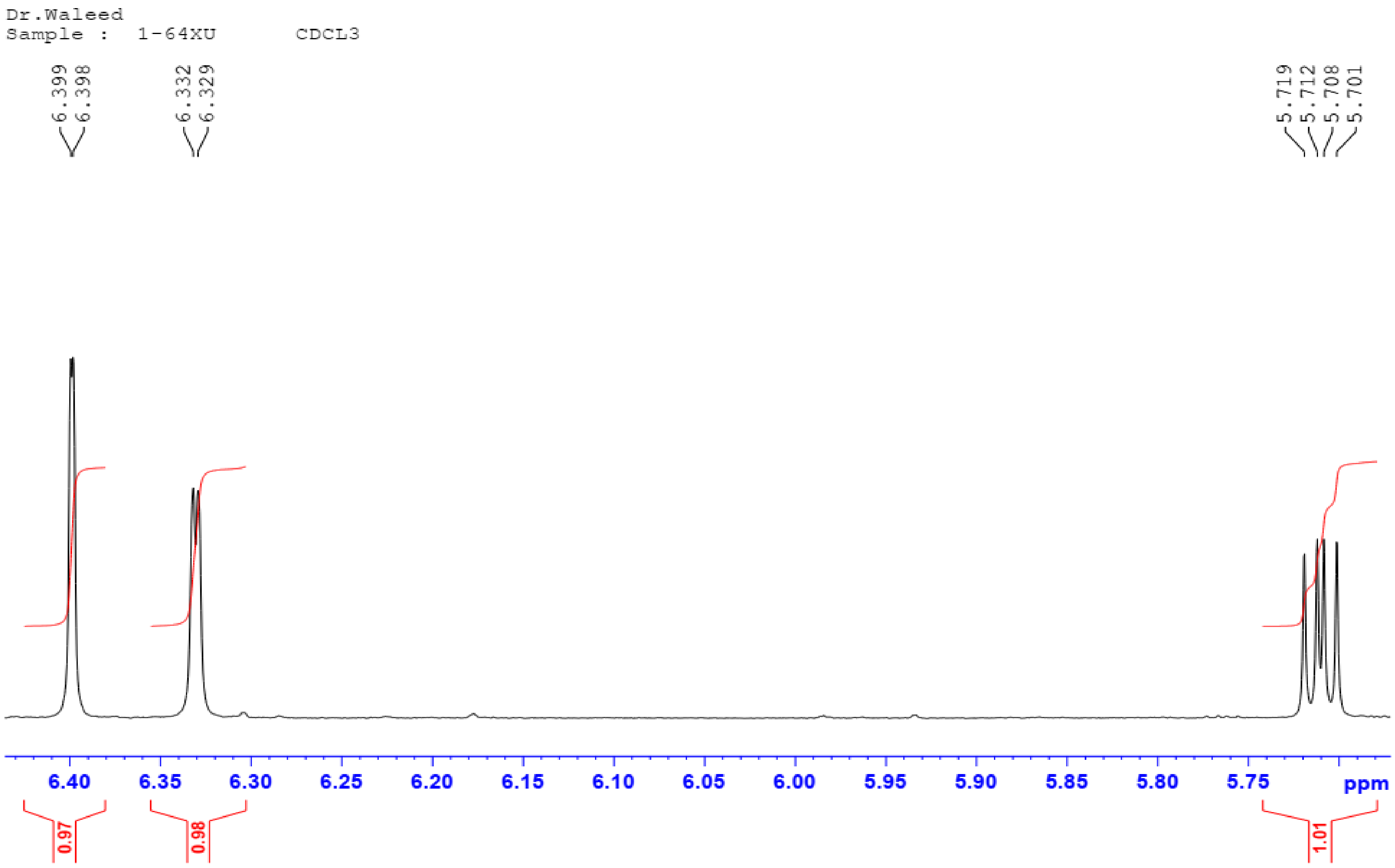


**Figure S2b.** $^{1}$H NMR spectrum of compound **5**

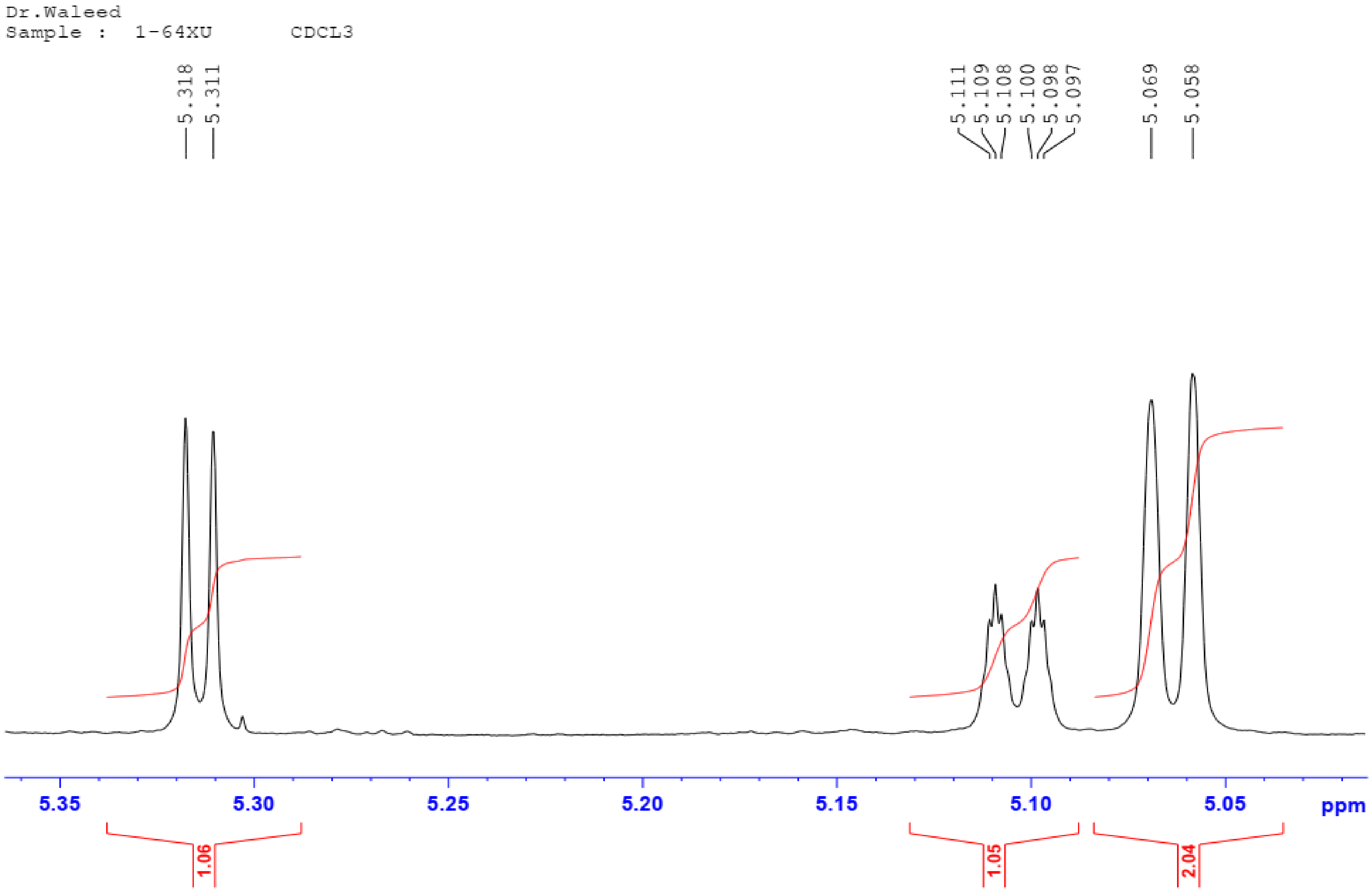


**Figure S2c.** $^{1}$H NMR spectrum of compound **5**

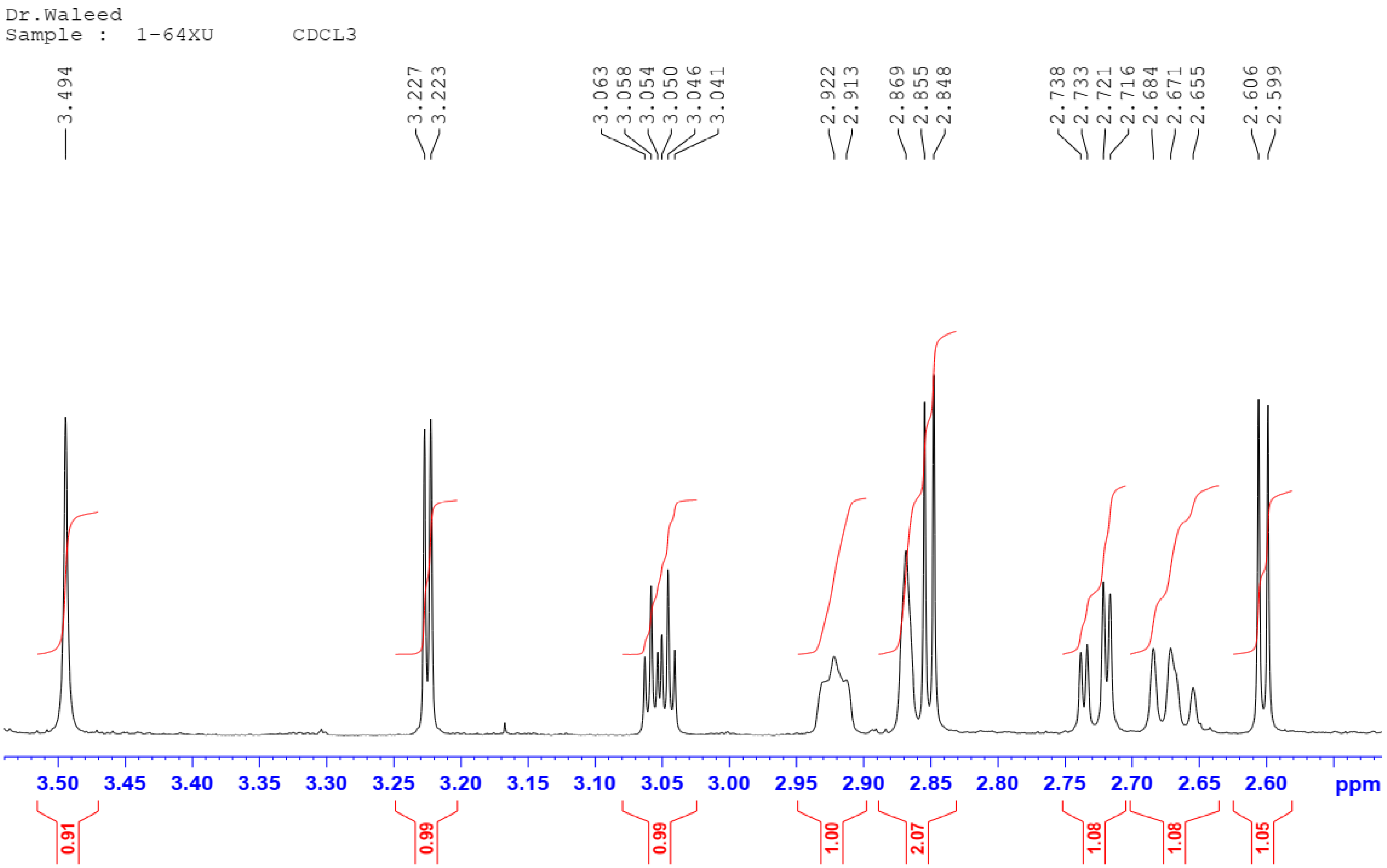


**Figure S2d.** $^{1}$H NMR spectrum of compound **5**

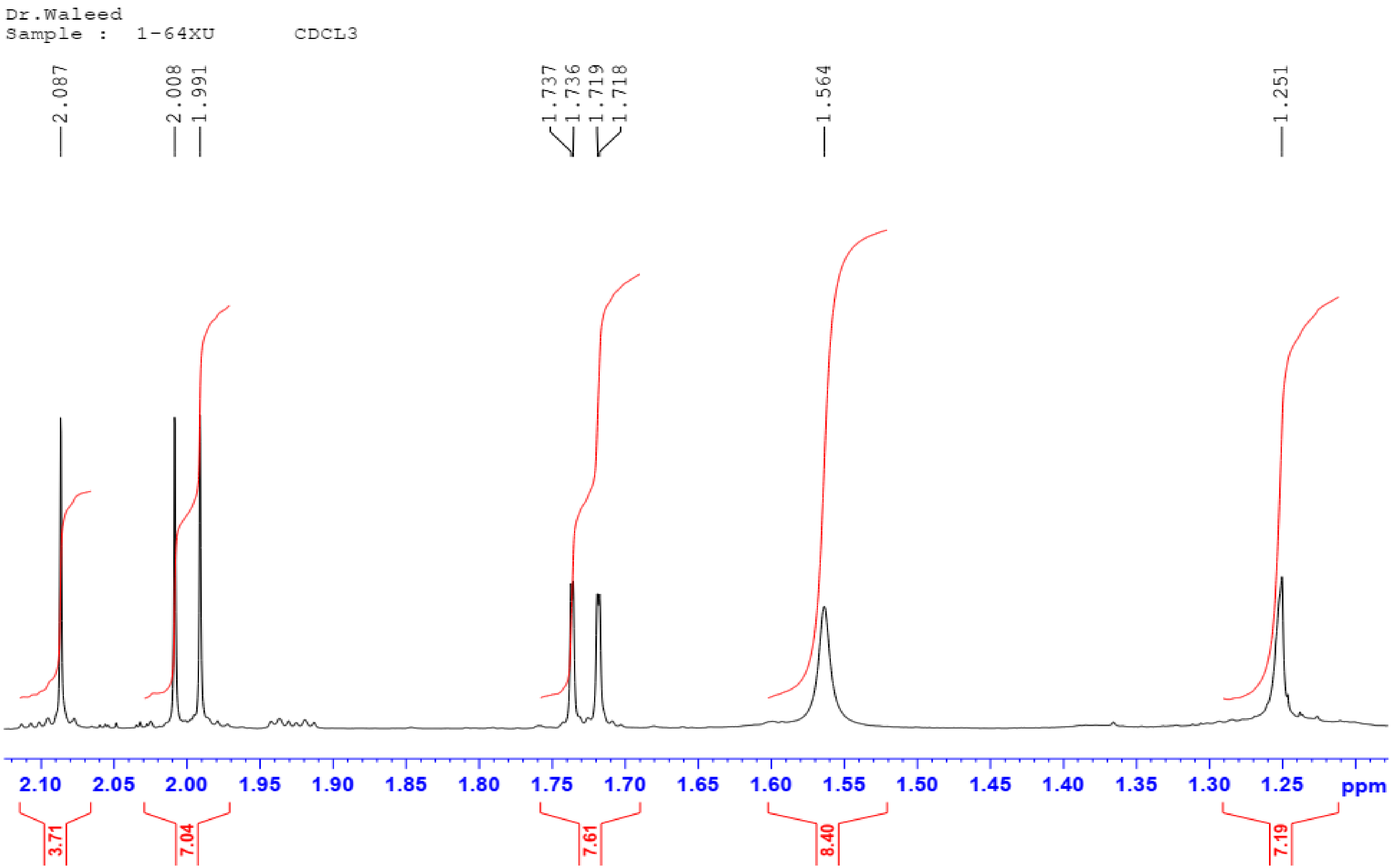


**Figure S2e.** $^{1}H$ NMR spectrum of compound **5**

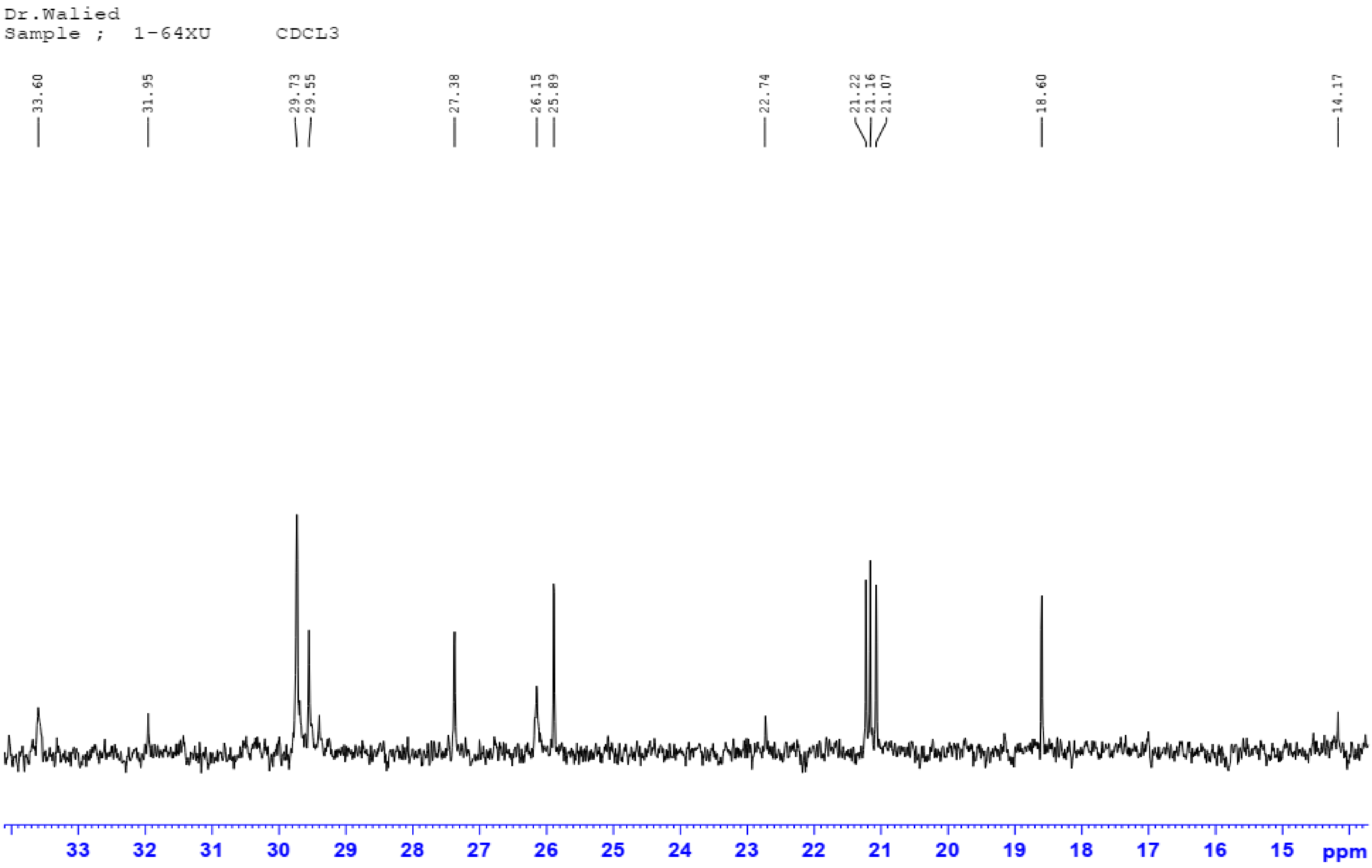


**Figure S2f.** $^{13}C$ NMR spectrum of compound **5**

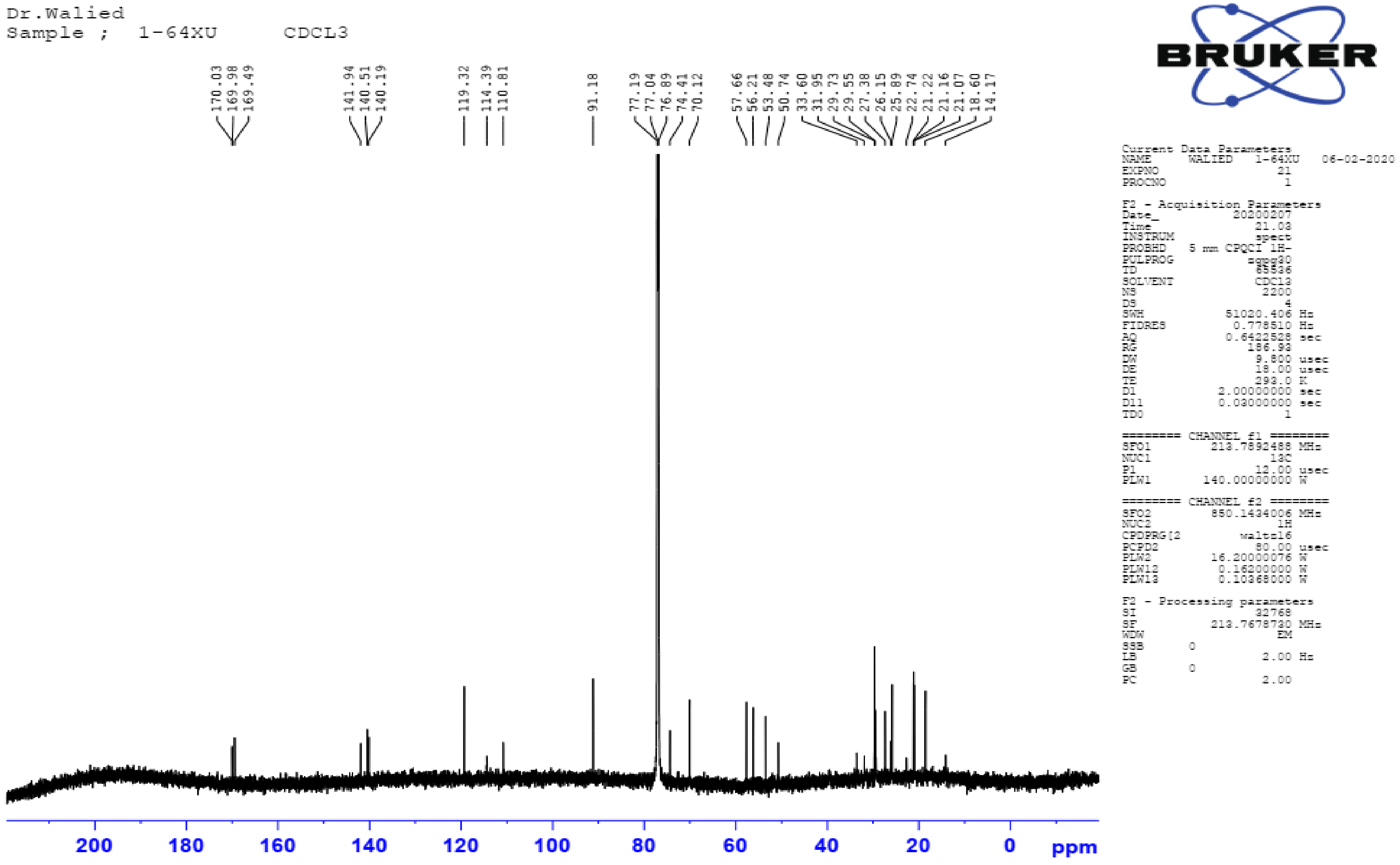


**Figure S2g.** $^{13}C$ NMR spectrum of compound **5**

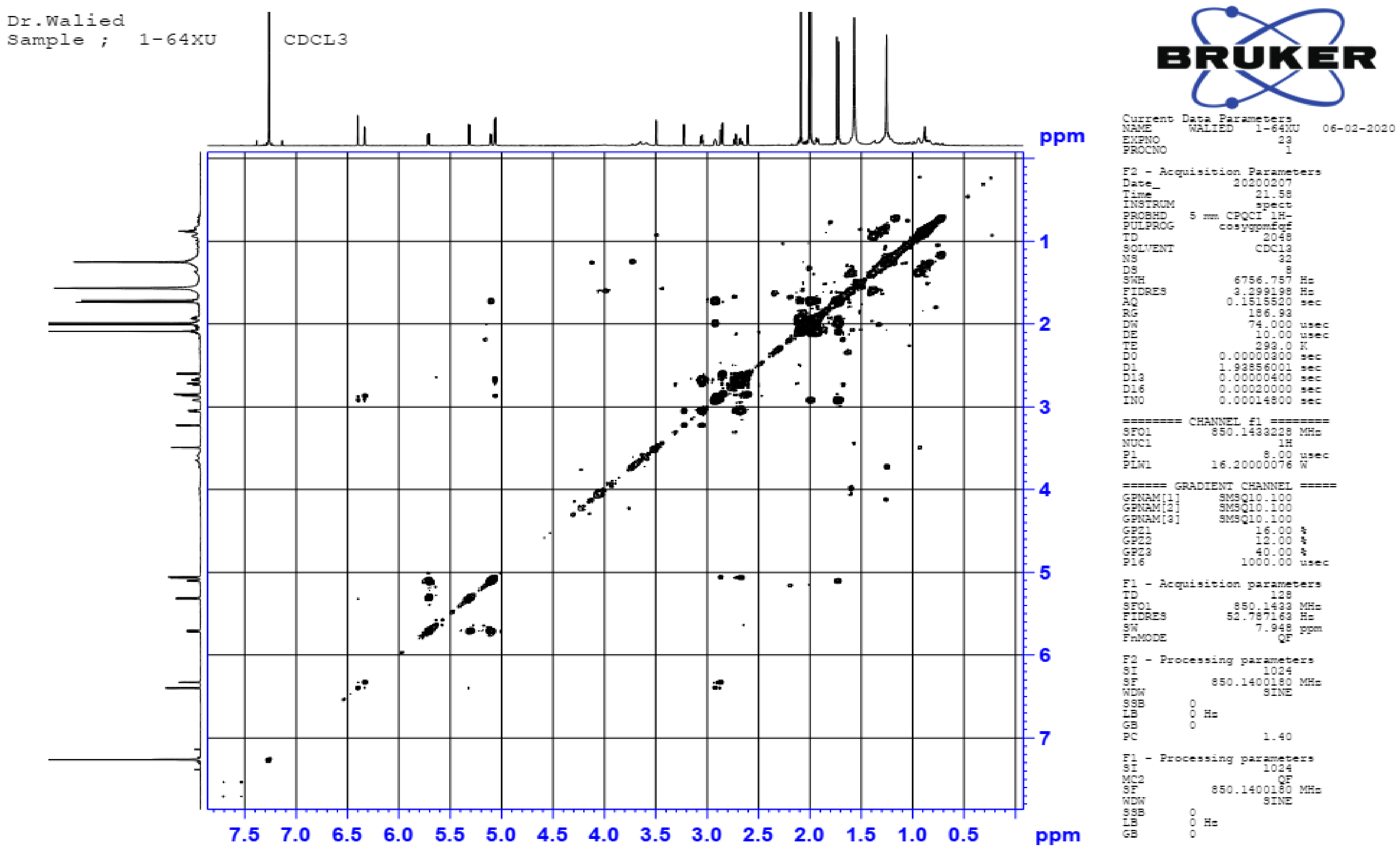


**Figure S2h.** $^{1}H$-$^{1}H$ COSY NMR spectrum of compound **5**

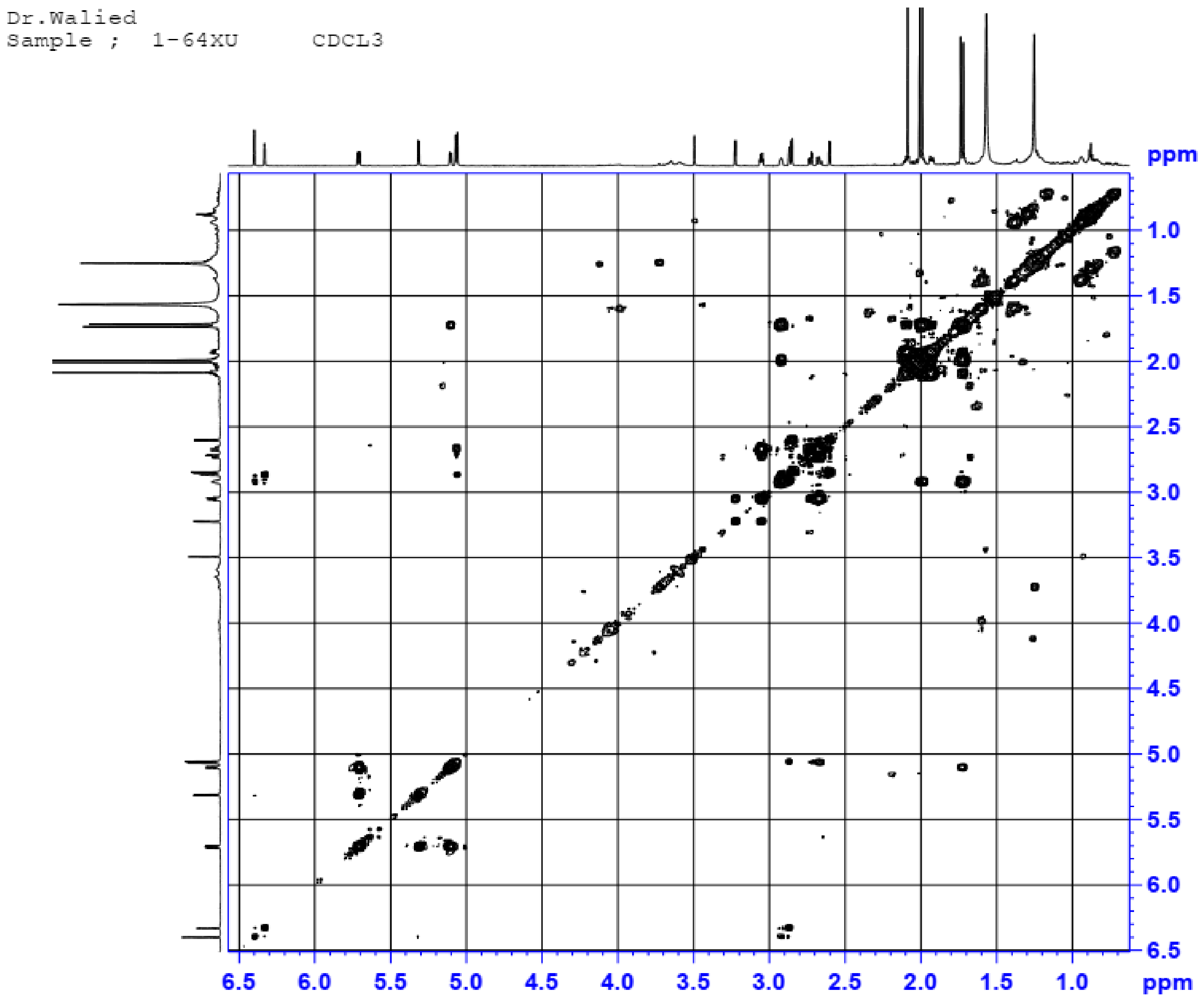


**Figure S2i.** $^{1}H$-$^{1}H$ COSY NMR spectrum of compound **5**

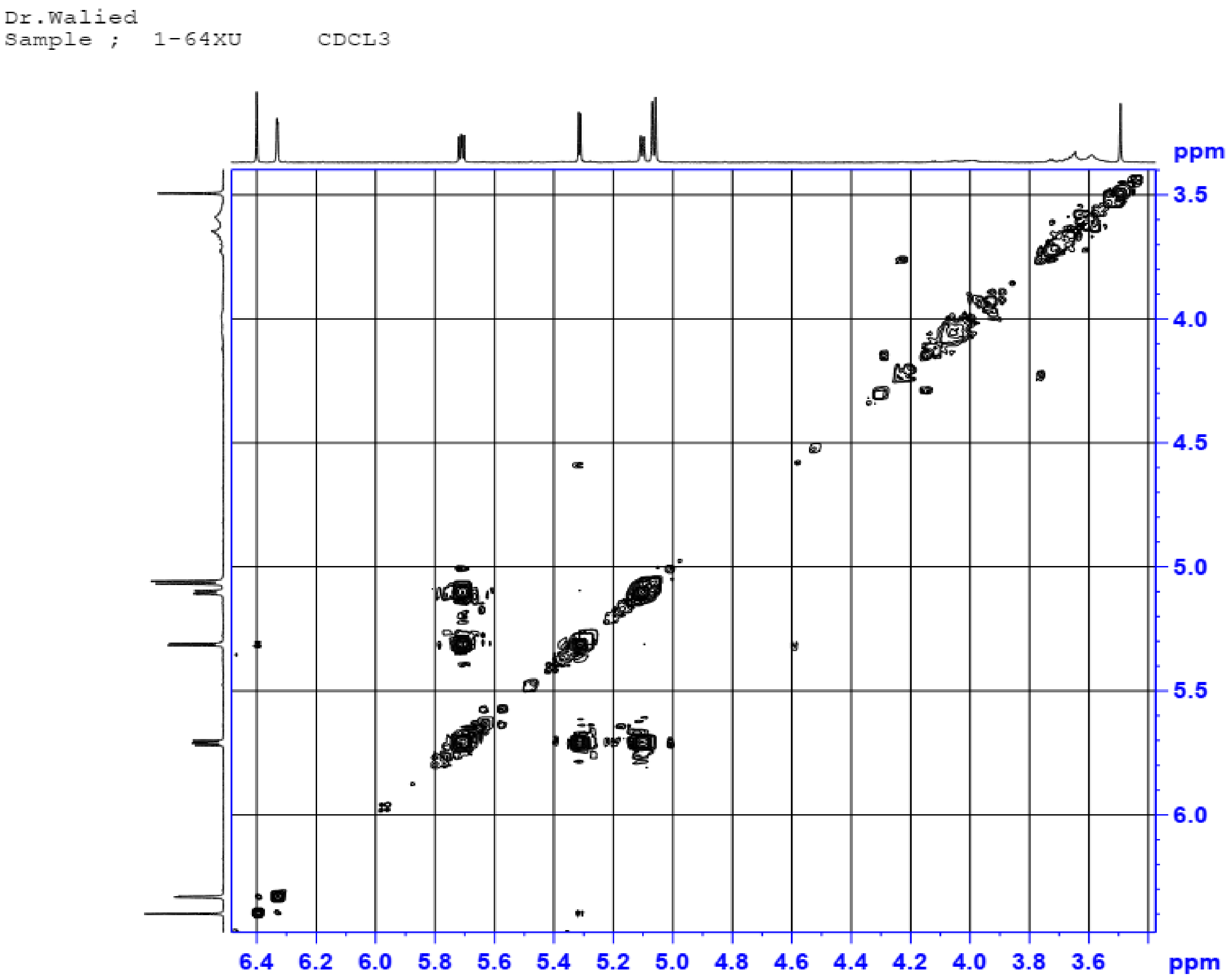


**Figure S2j.** $^{1}H$-$^{1}H$ COSY NMR spectrum of compound **5**

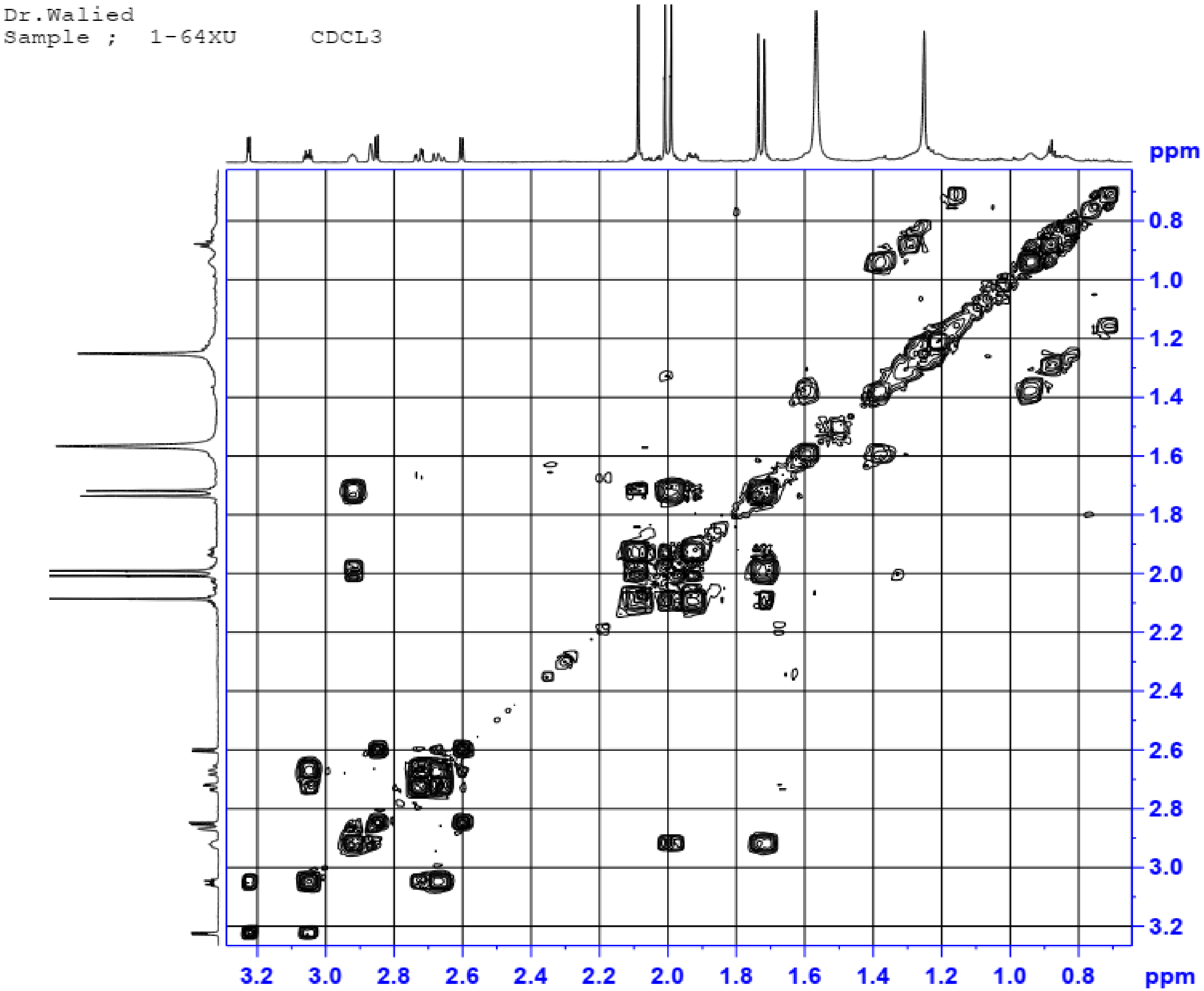


**Figure S2k.** $^1H$-$^1H$ COSY spectrum NMR of compound **5**

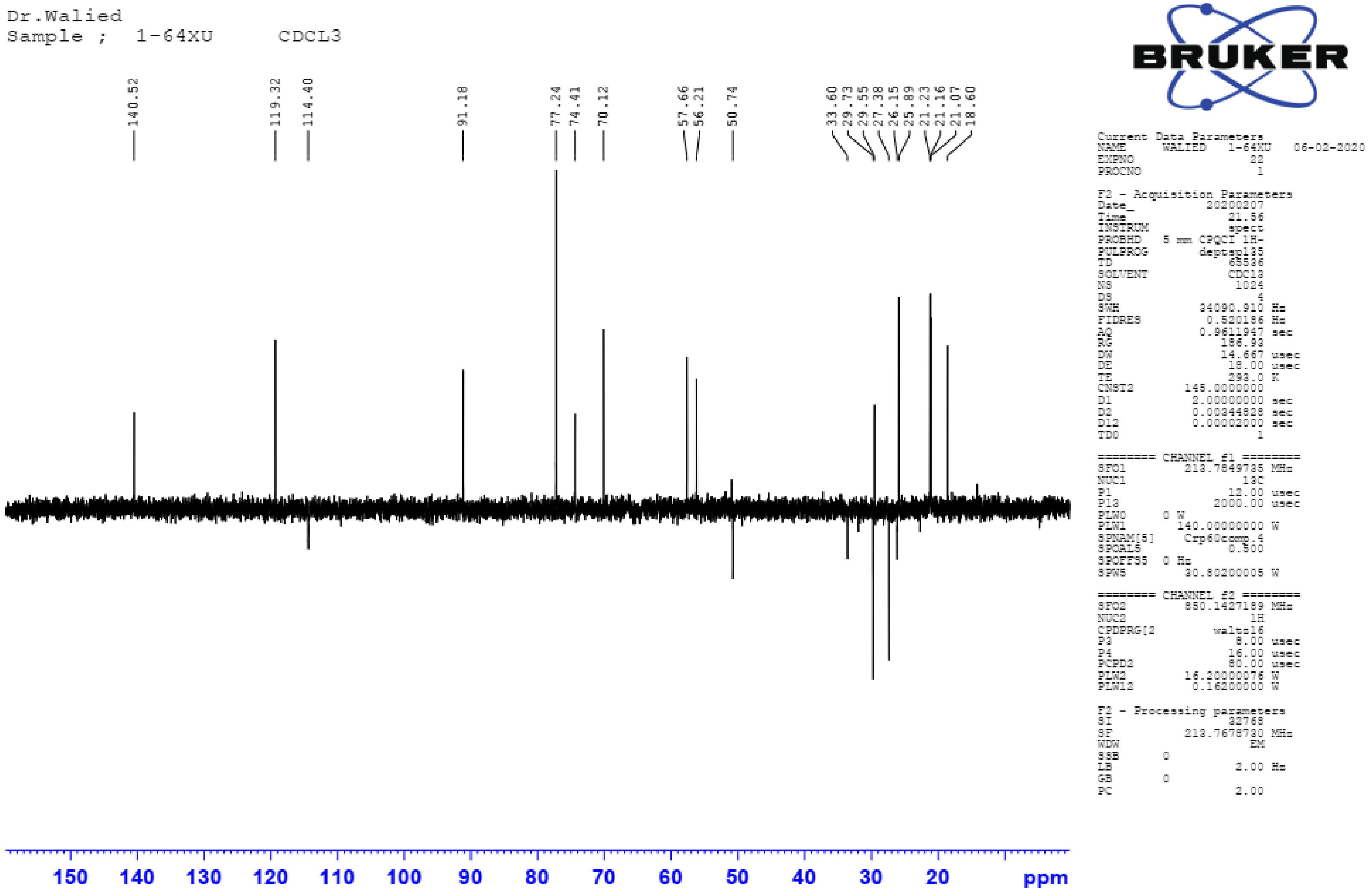


**Figure S21.** $^{13}C$ DEPT NMR spectrum of compound **5**

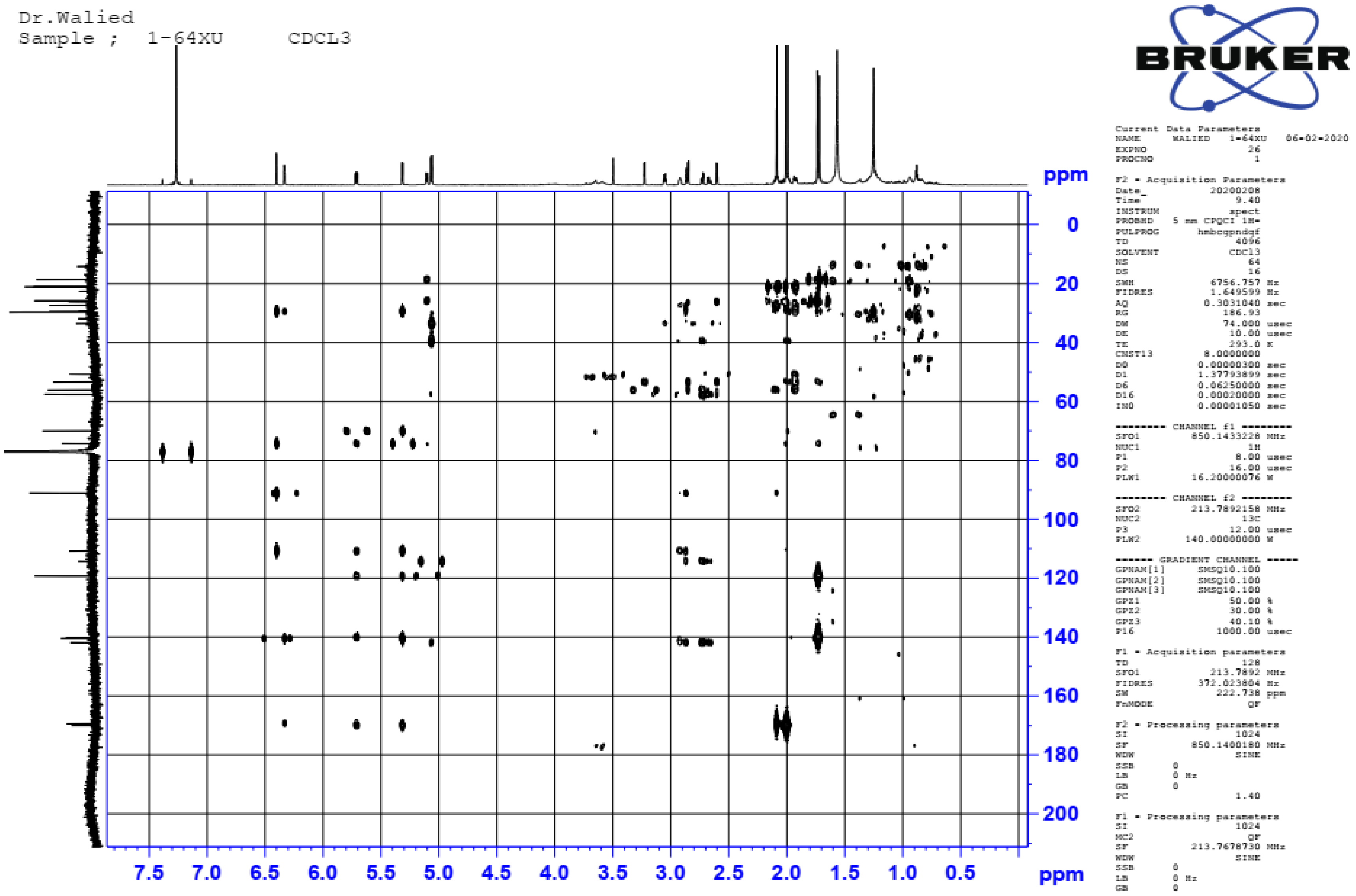


**Figure S2m.** $^{1}H$-$^{13}C$ HMBC NMR spectrum of compound **5**

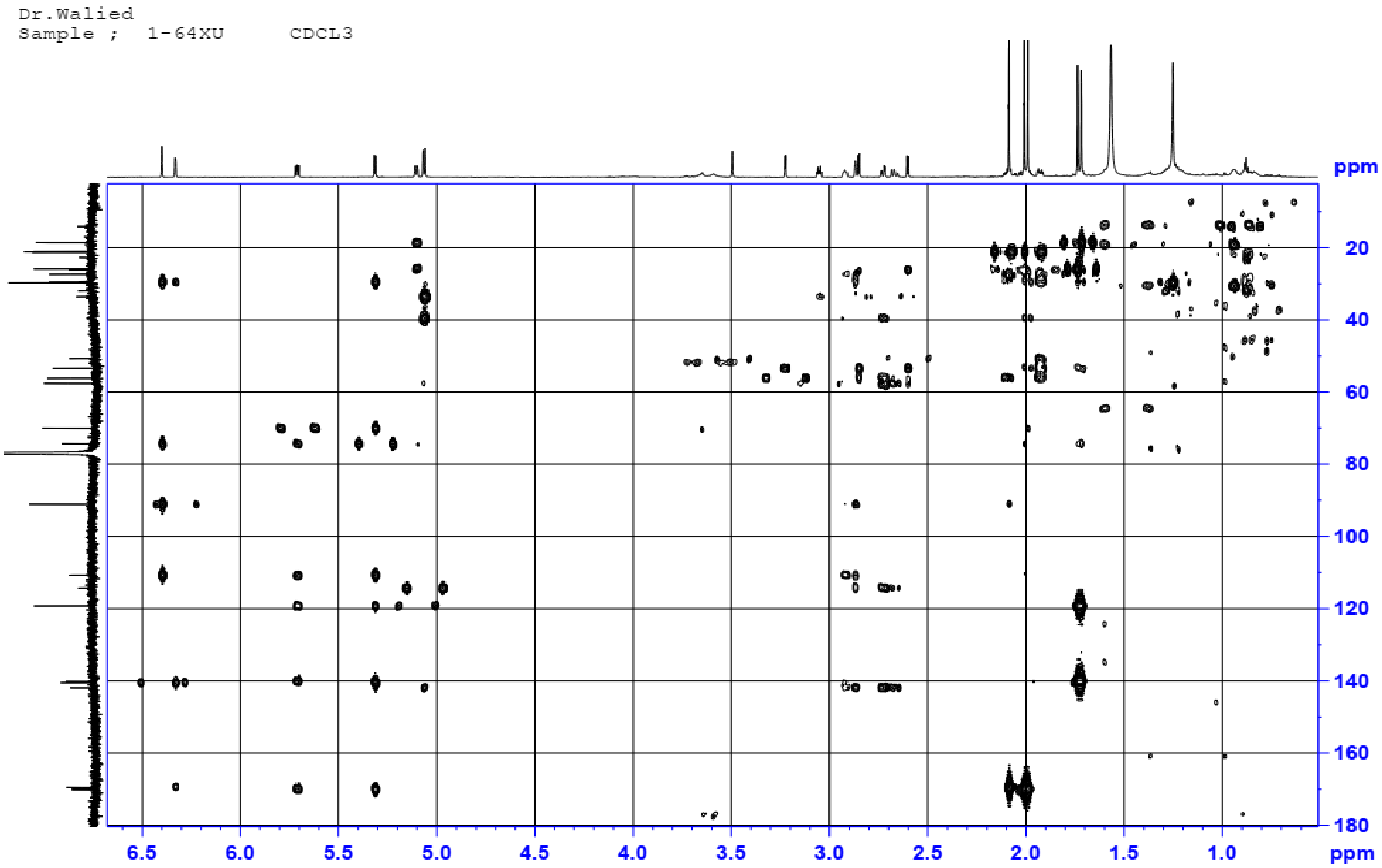


**Figure S2n.** $^{1}H$-$^{13}C$ HMBC NMR spectrum of compound **5**

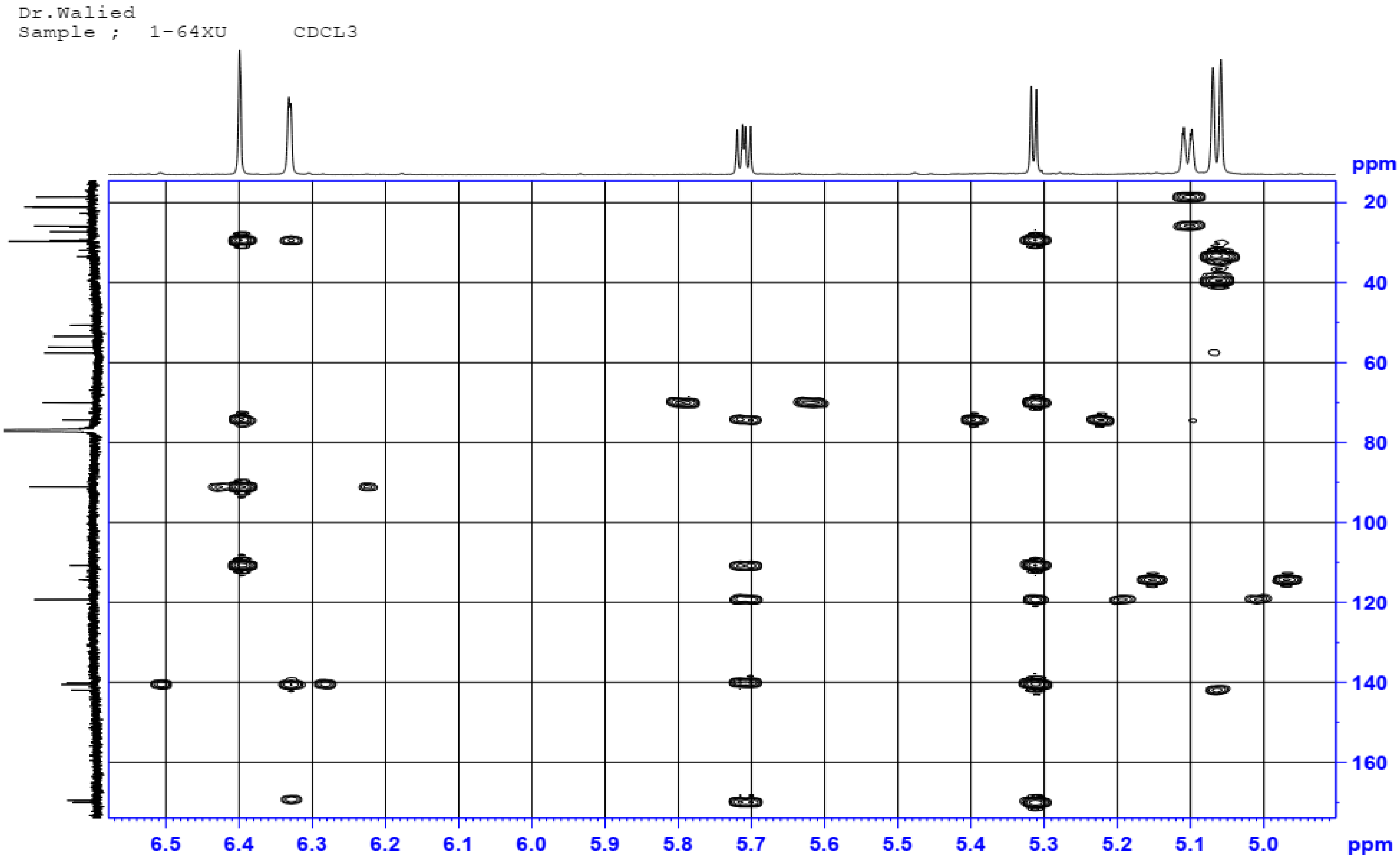


**Figure S2o.** $^{1}H$-$^{13}C$ HMBC NMR spectrum of compound **5**

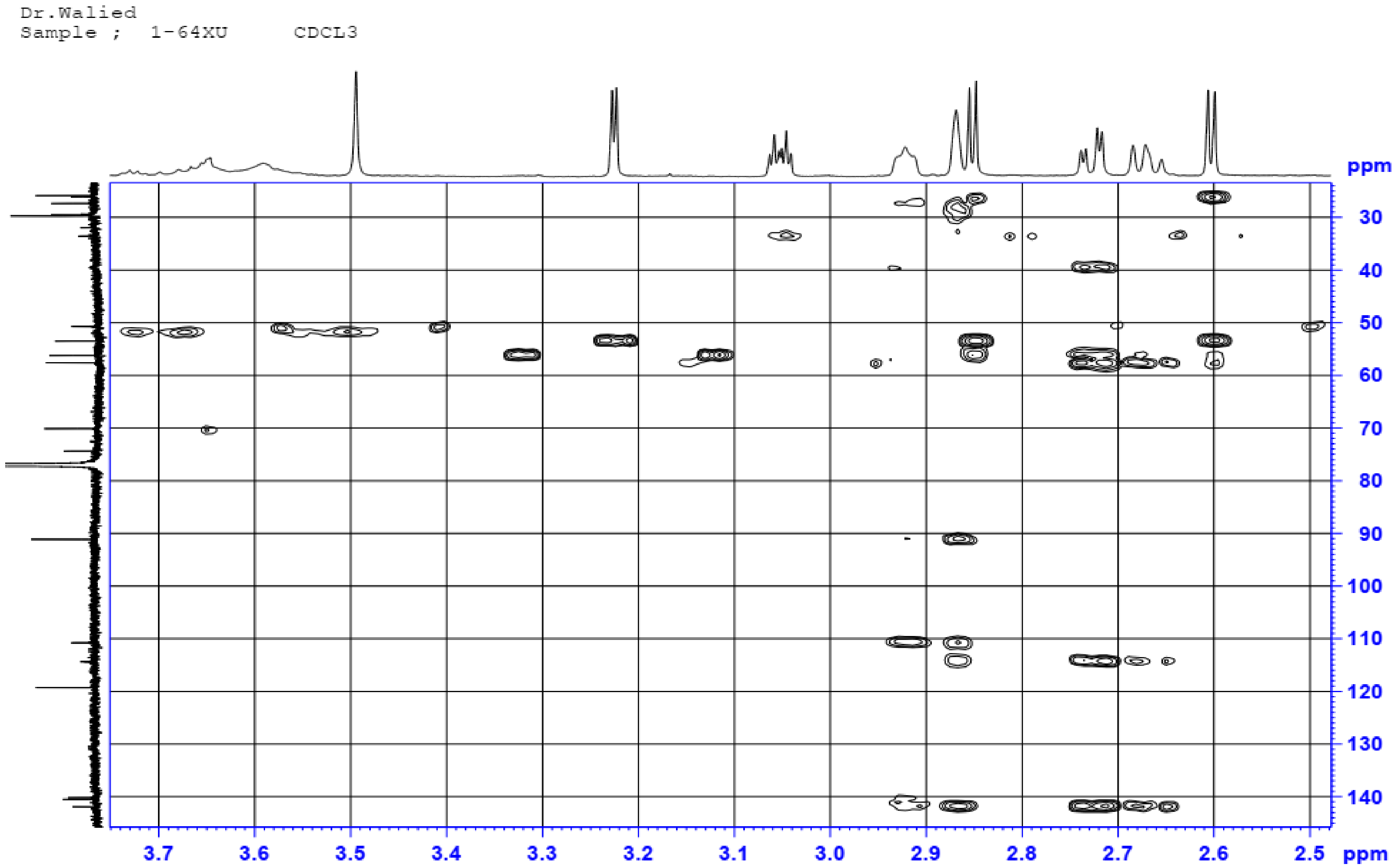


**Figure S2p.** $^{1}H$-$^{13}C$ HMBC NMR spectrum of compound **5**

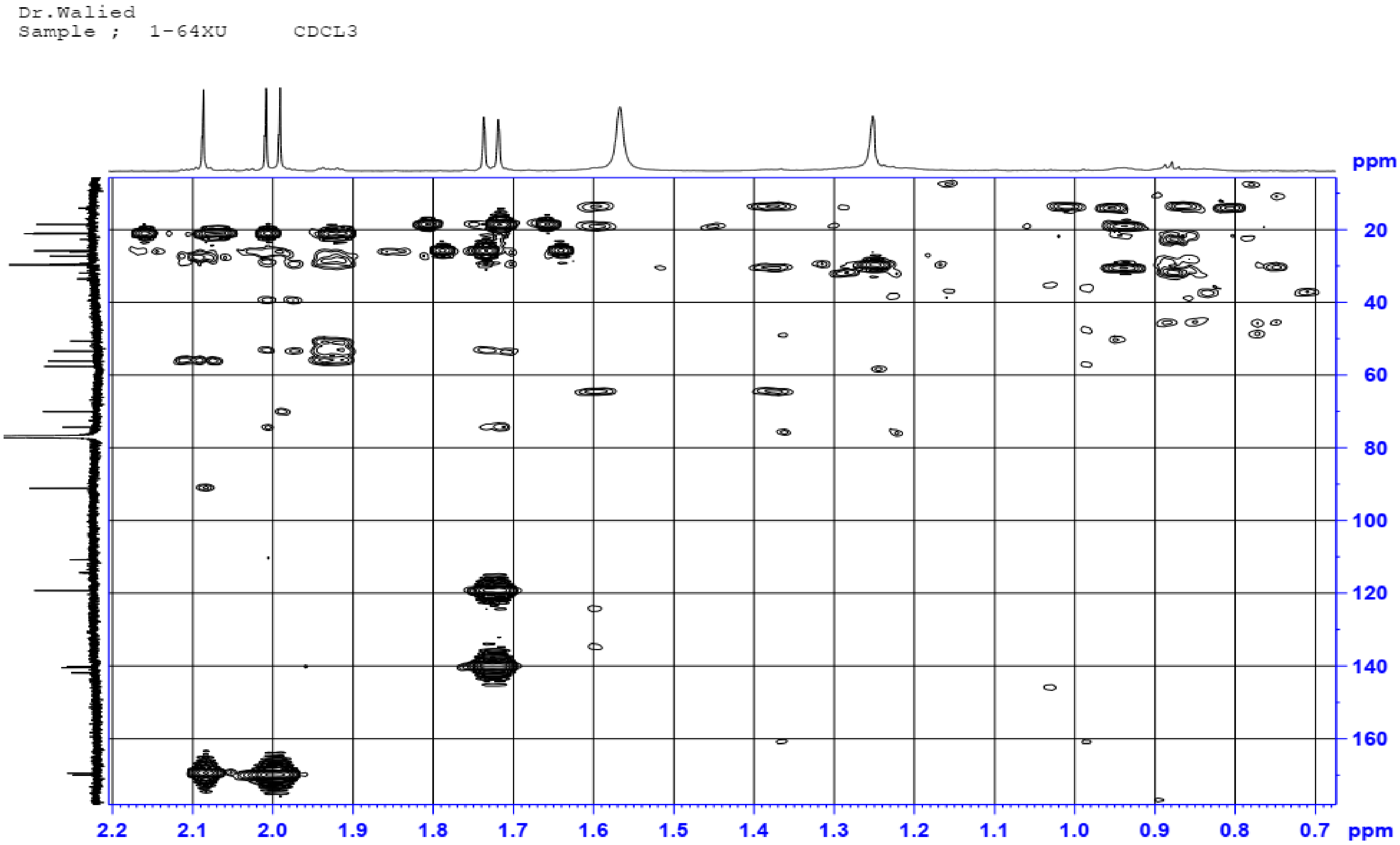


**Figure S2q.** $^{1}H$-$^{13}C$ HMBC NMR spectrum of compound **5**

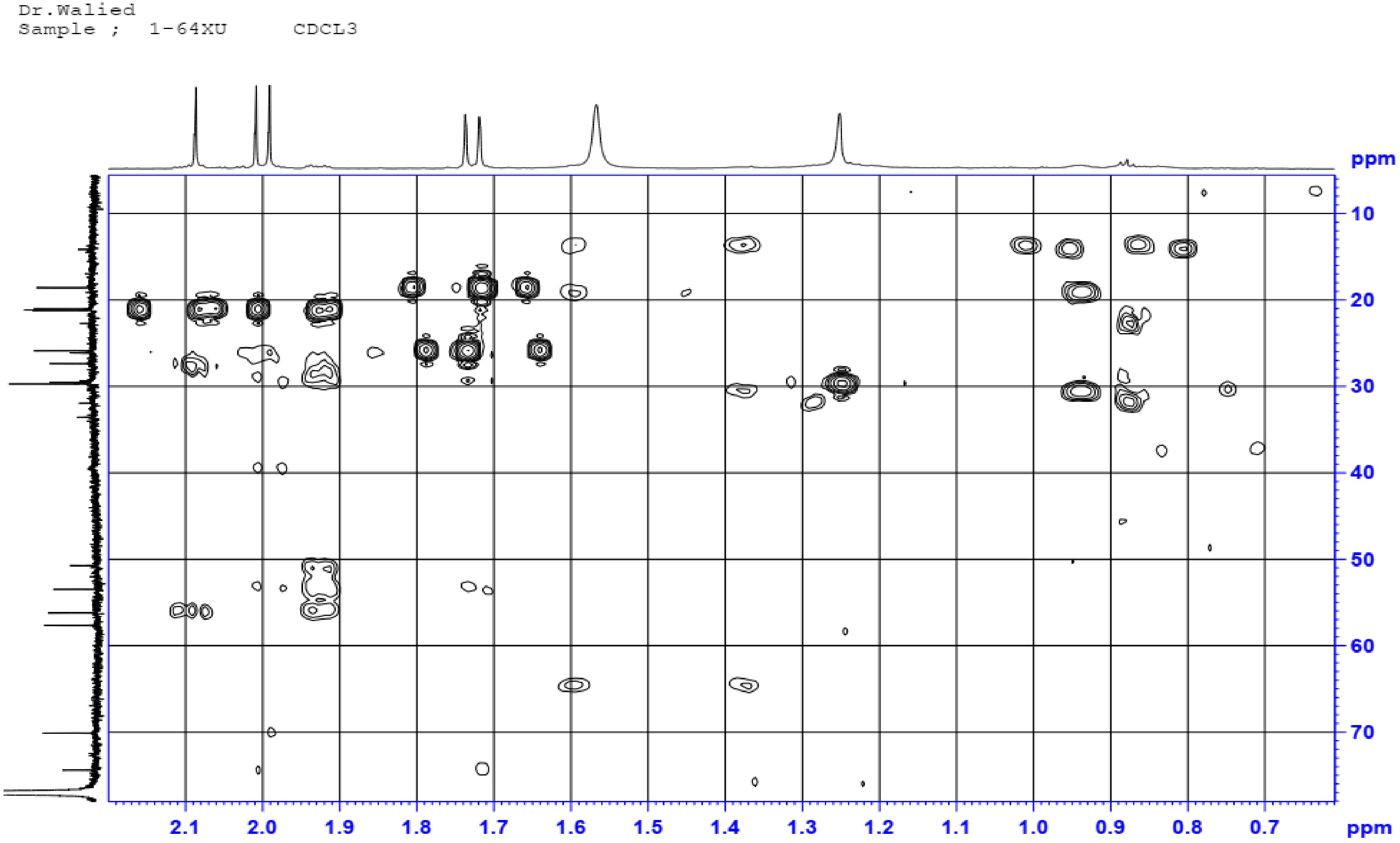


**Figure S2r.** $^{1}H$-$^{13}C$ HMBC NMR spectrum of compound **5**

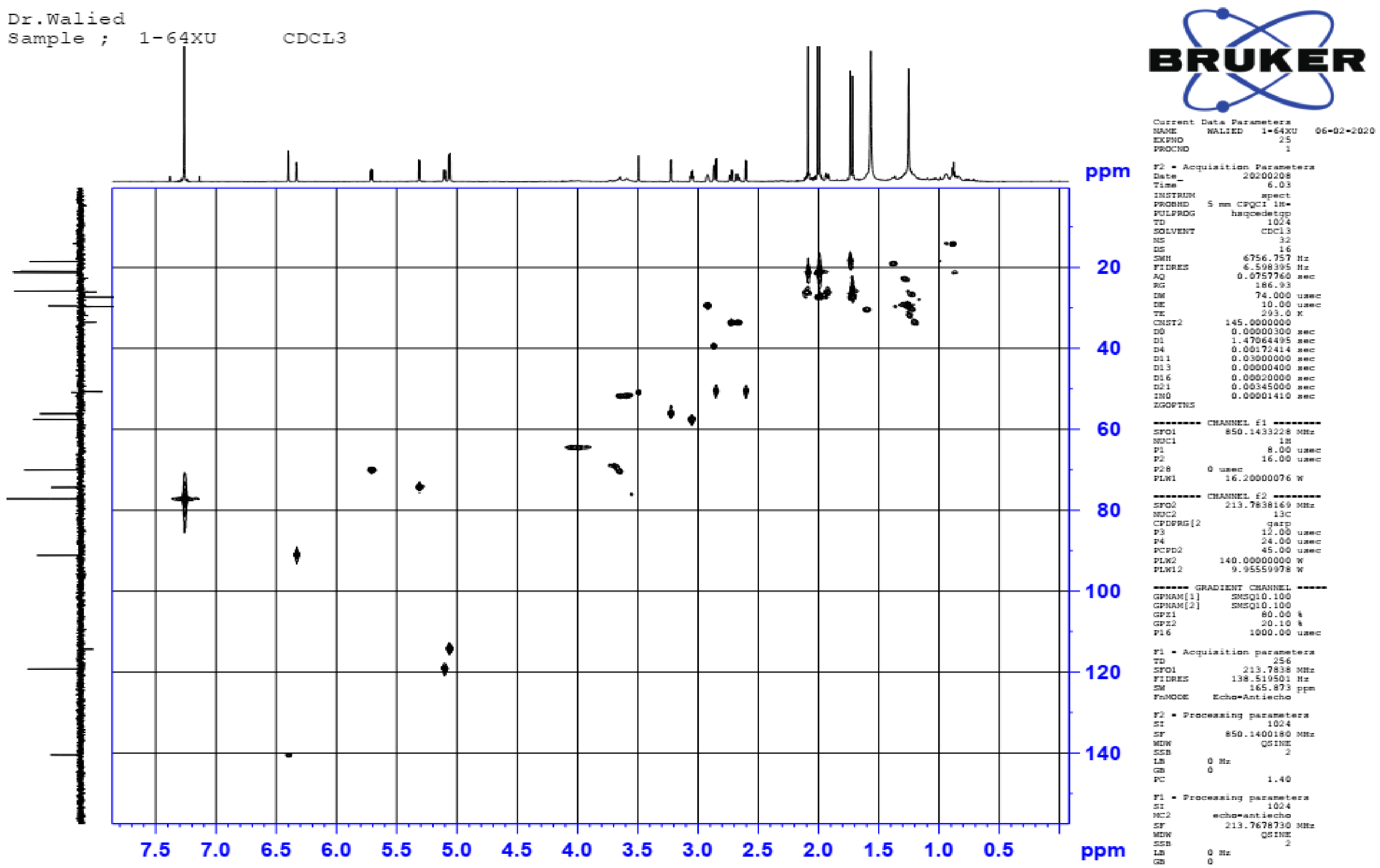


**Figure S2s.** $^{1}$H-$^{13}$C HSQC NMR spectrum of compound **5**

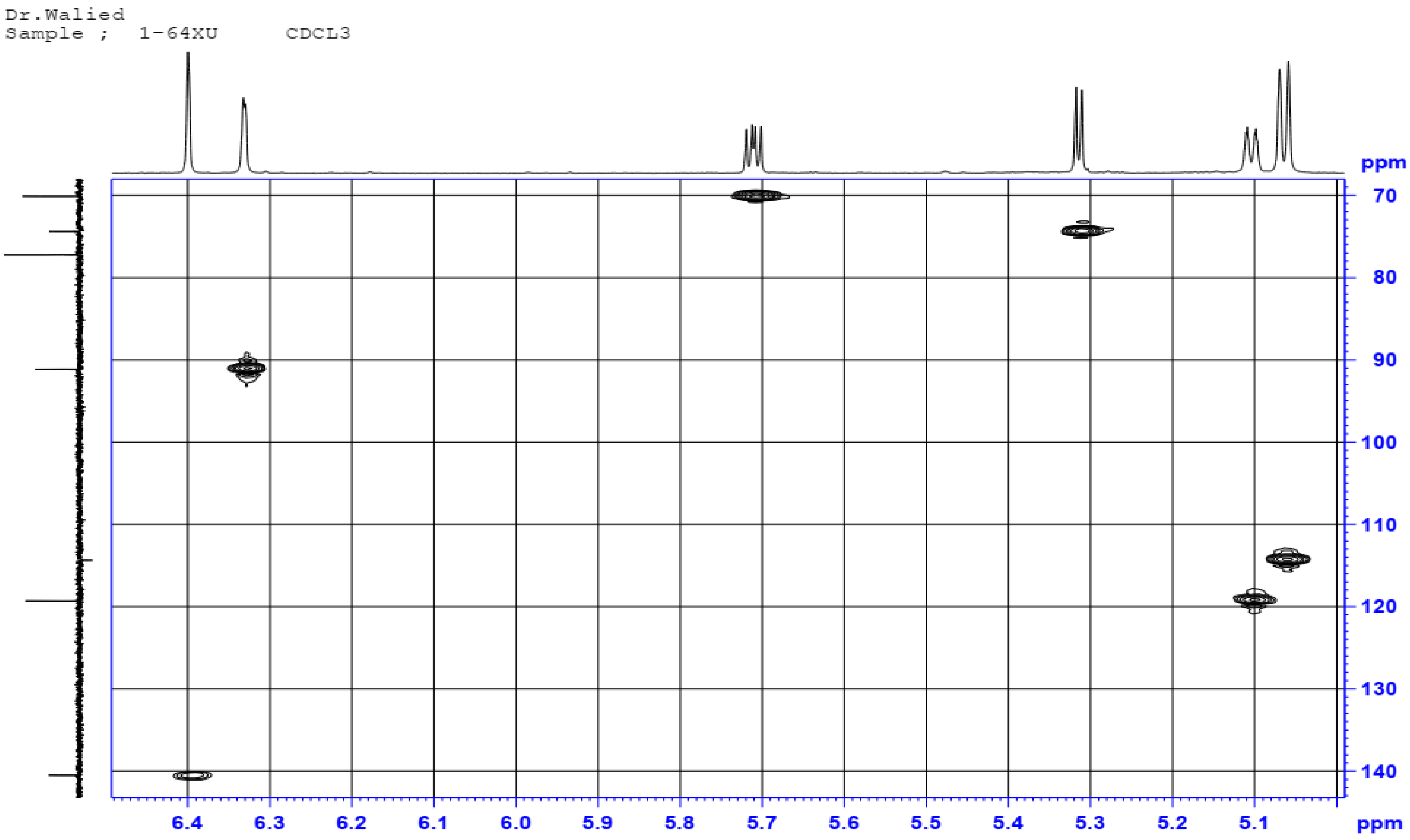


**Figure S2t.** $^{1}H$-$^{13}C$ HSQC NMR spectrum of compound **5**

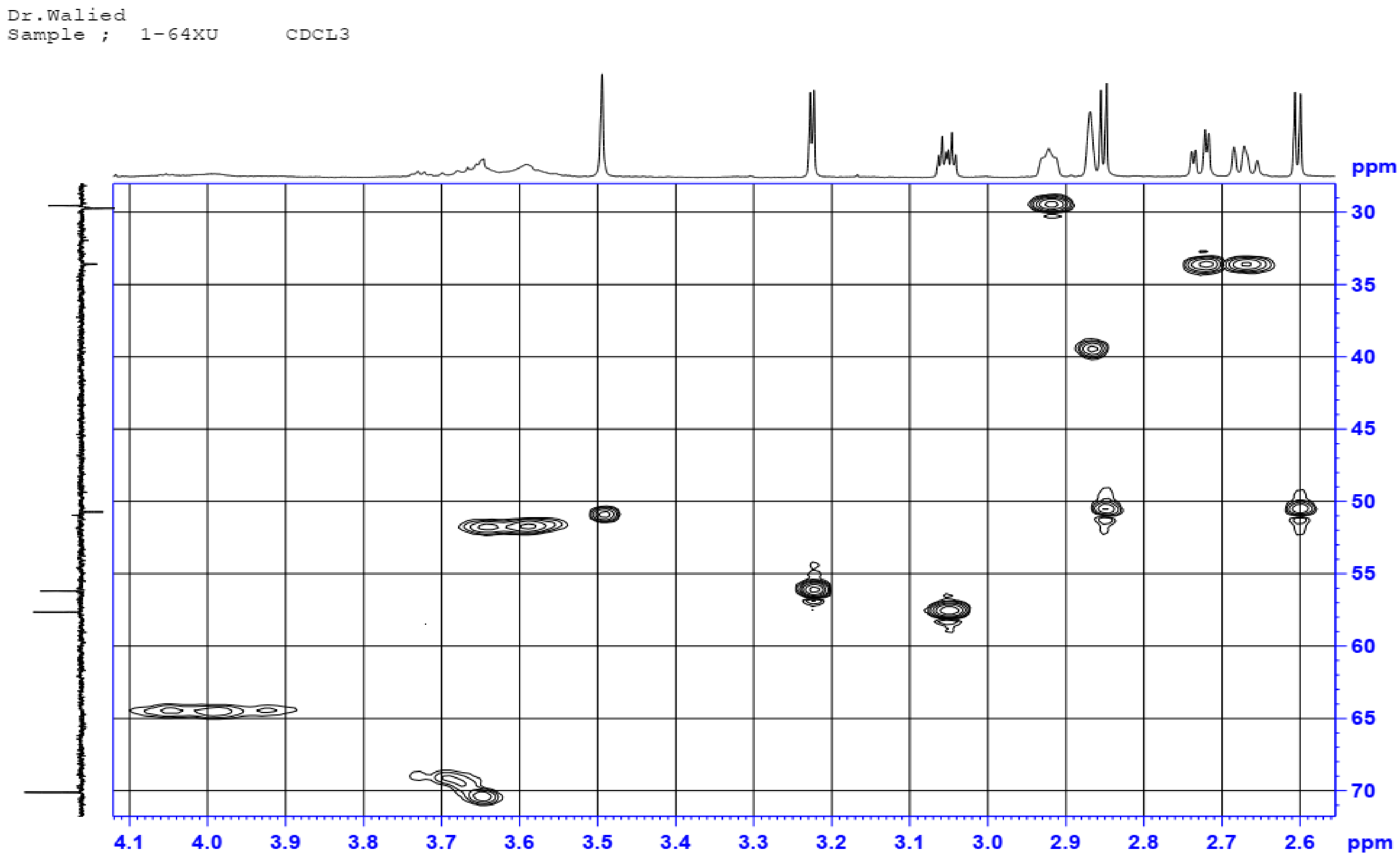


**Figure S2u.** $^{1}H$-$^{13}C$ HSQC NMR spectrum of compound **5**

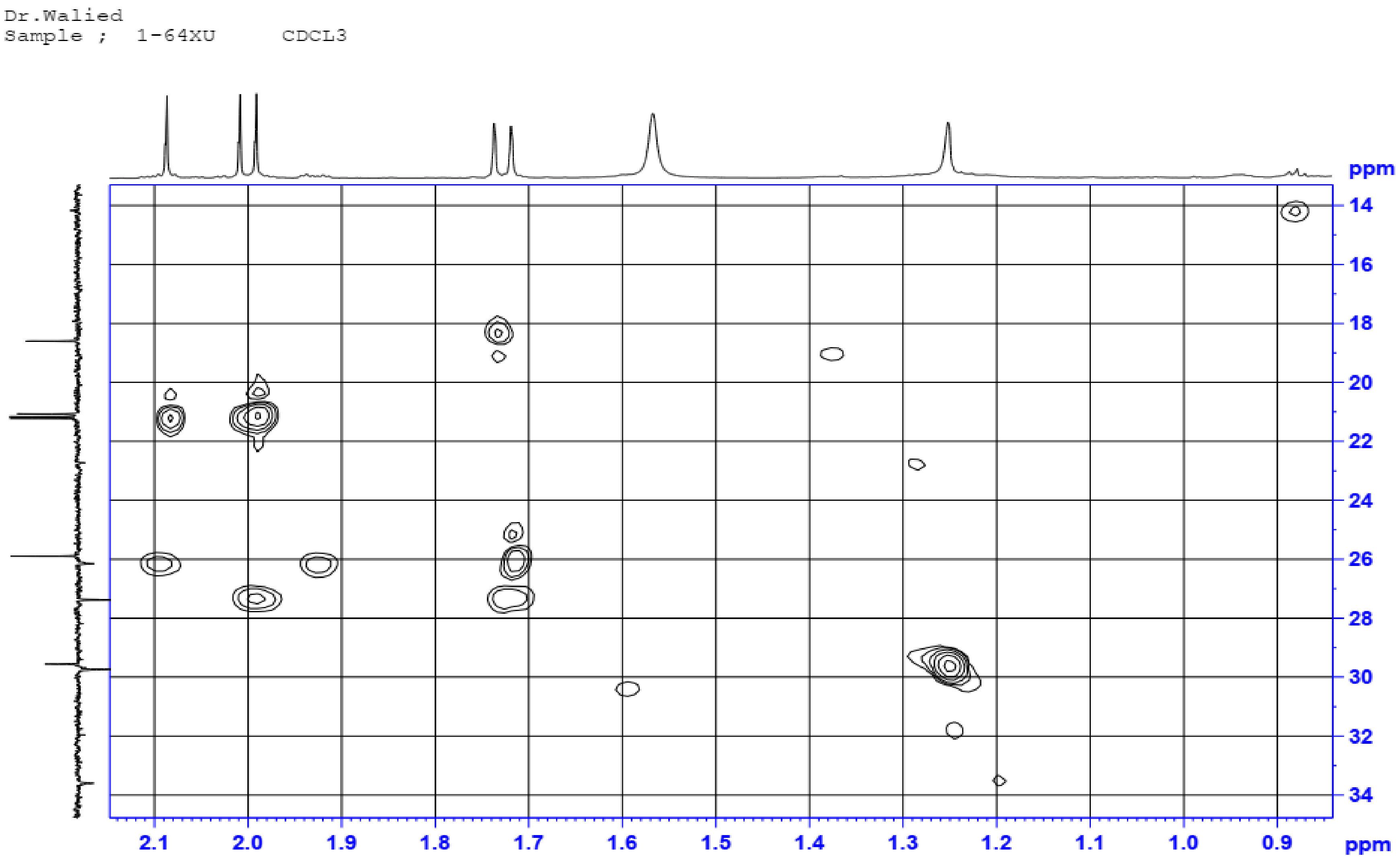


**Figure S2v.** $^1$H-$^{13}$C HSQC NMR spectrum of compound **5**

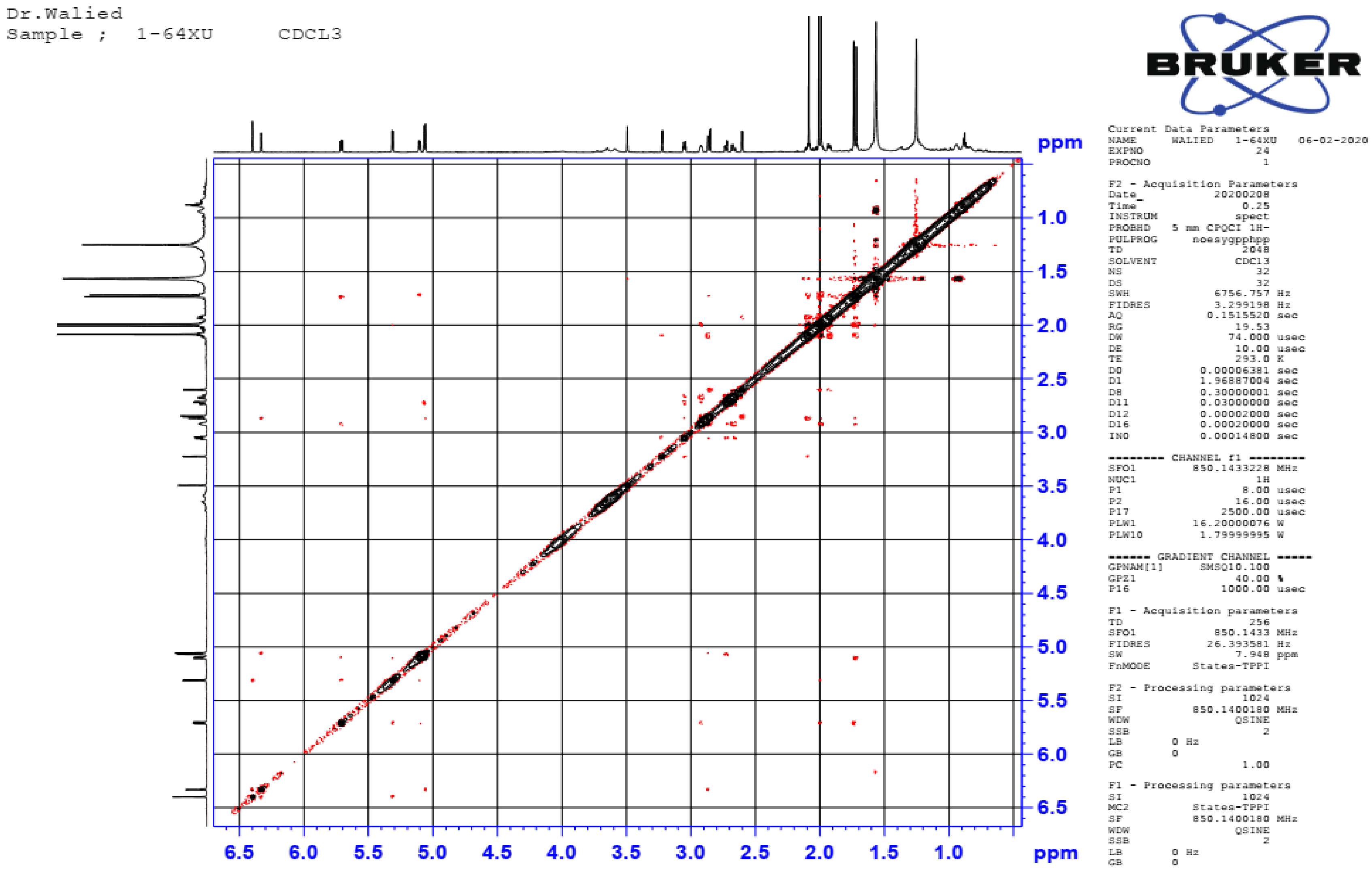


**Figure S2w.** $^{1}$H-$^{1}$H NOESY NMR spectrum of compound **5**

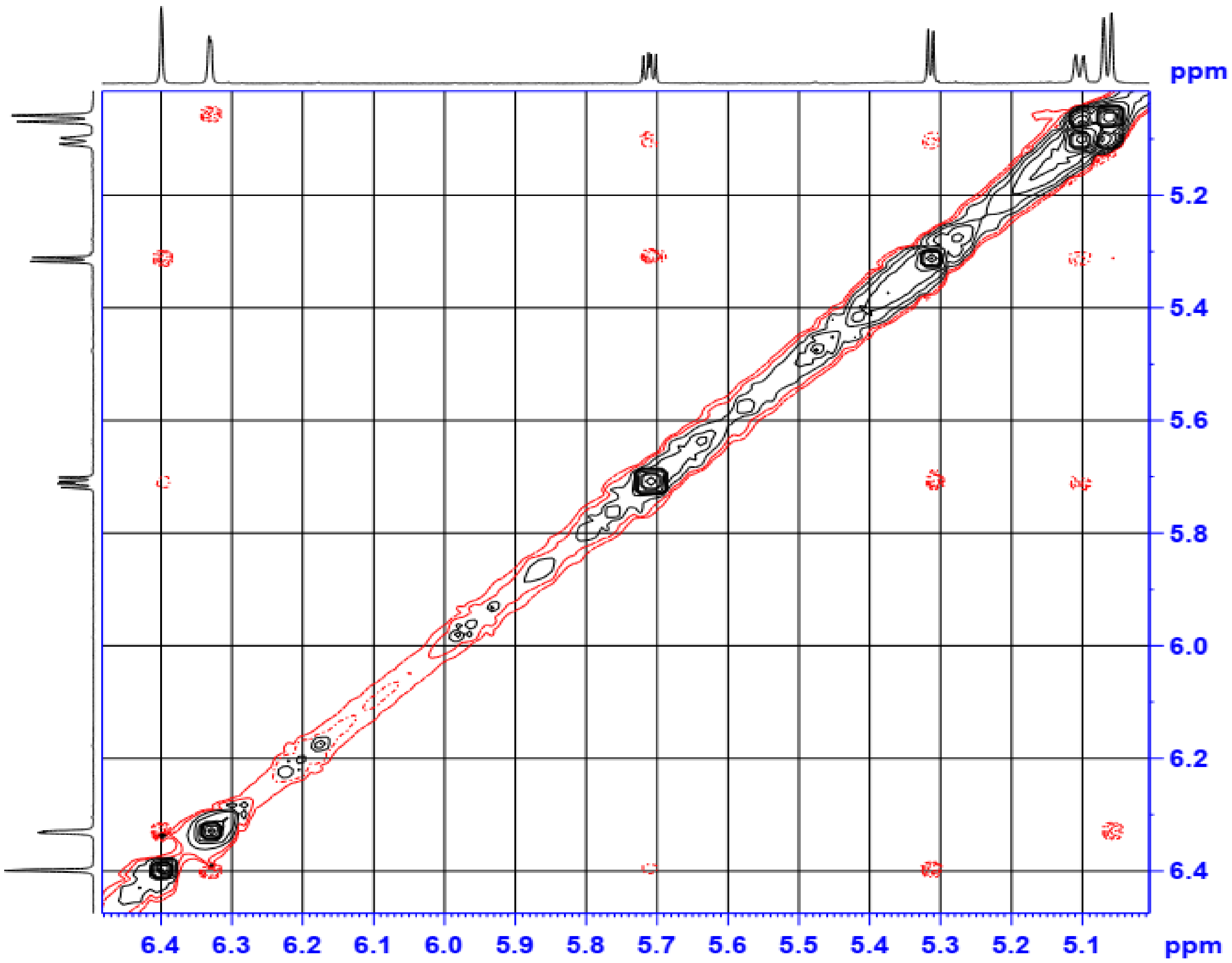


**Figure S2x.** $^{1}$H-$^{1}$H NOESY NMR spectrum of compound **5**

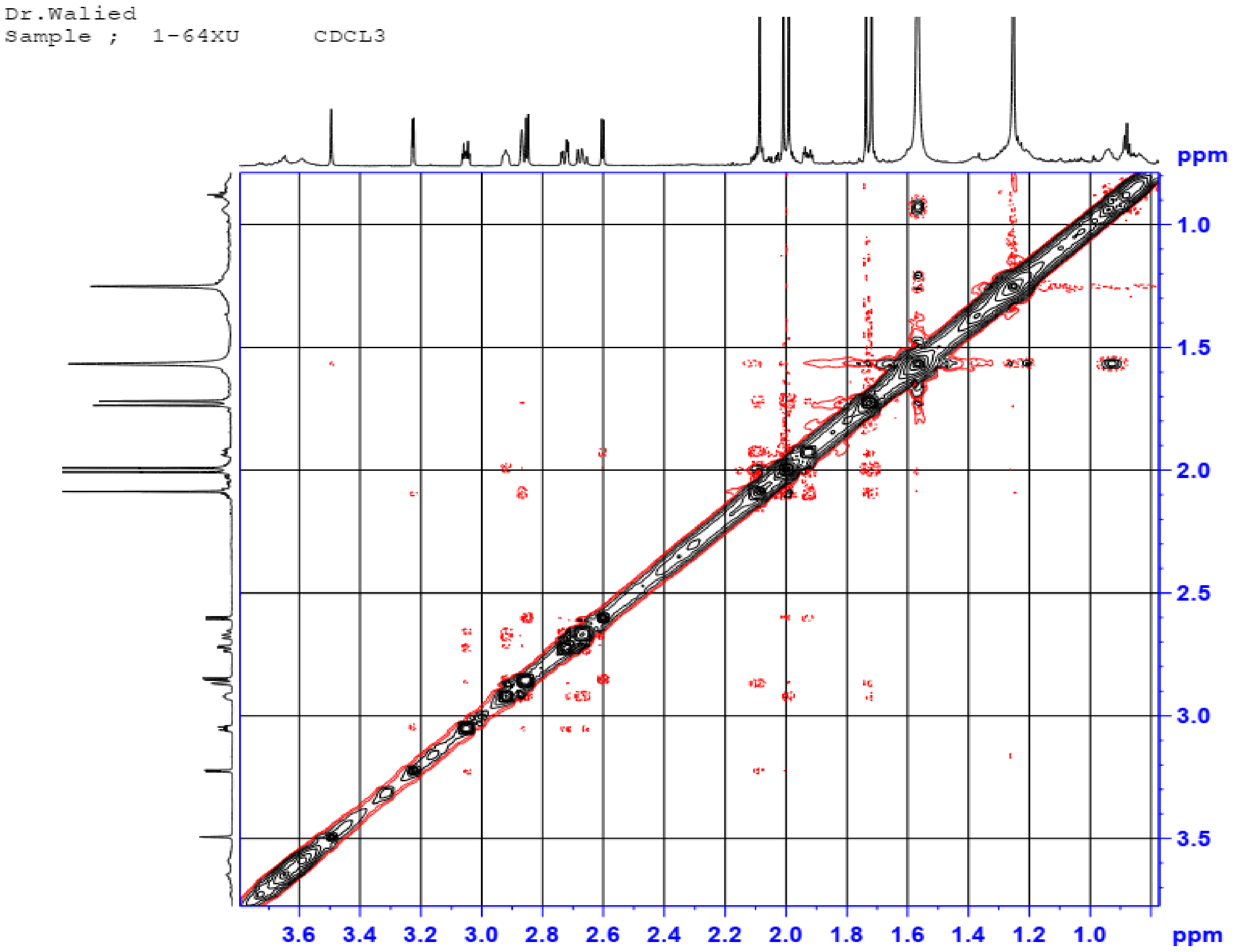


**Figure S2y.** $^{1}H$-$^{1}H$ NOESY NMR spectrum of compound **5**

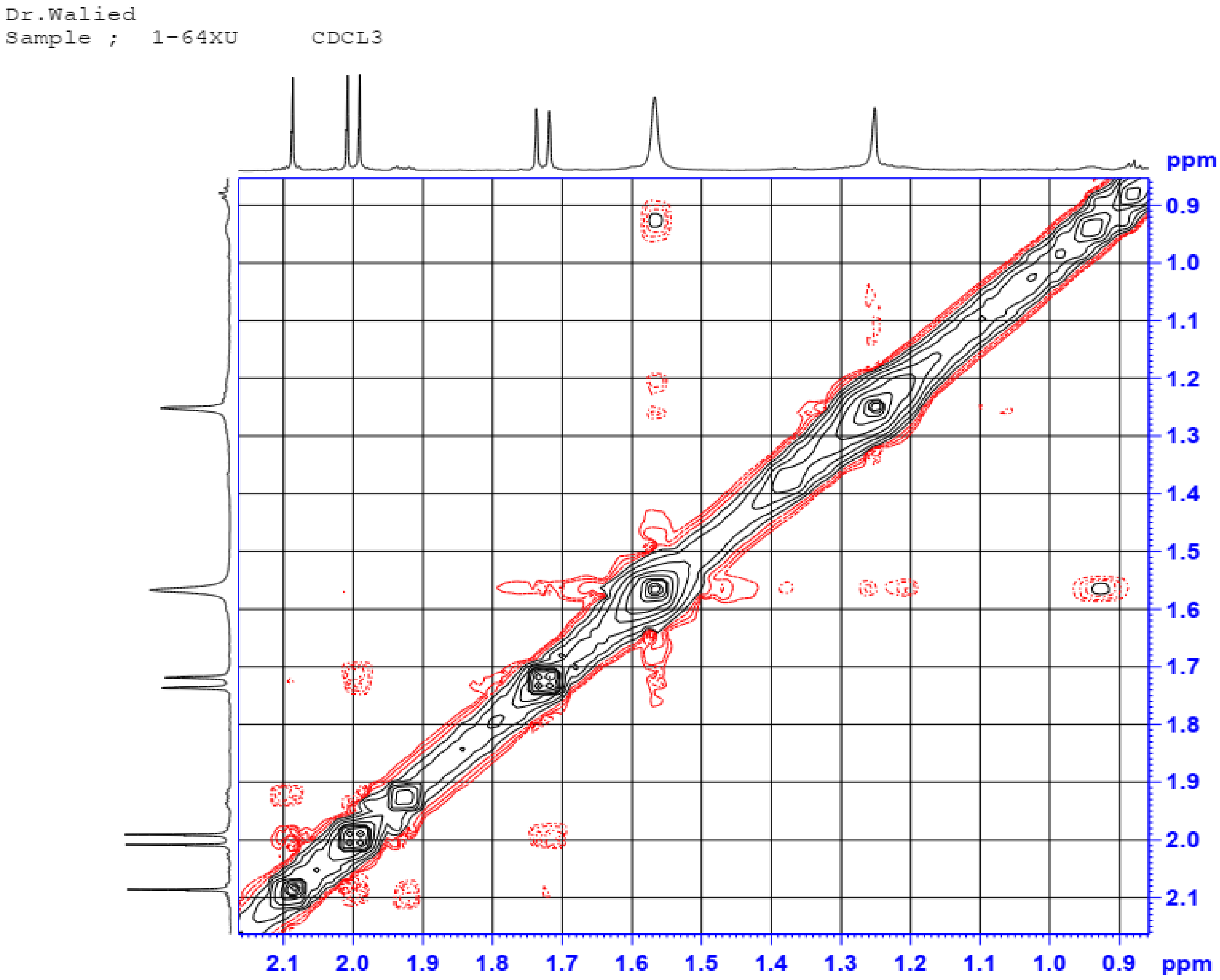


**Figure S2z.** $^{1}H$-$^{1}H$ NOESY NMR spectrum of compound **5**

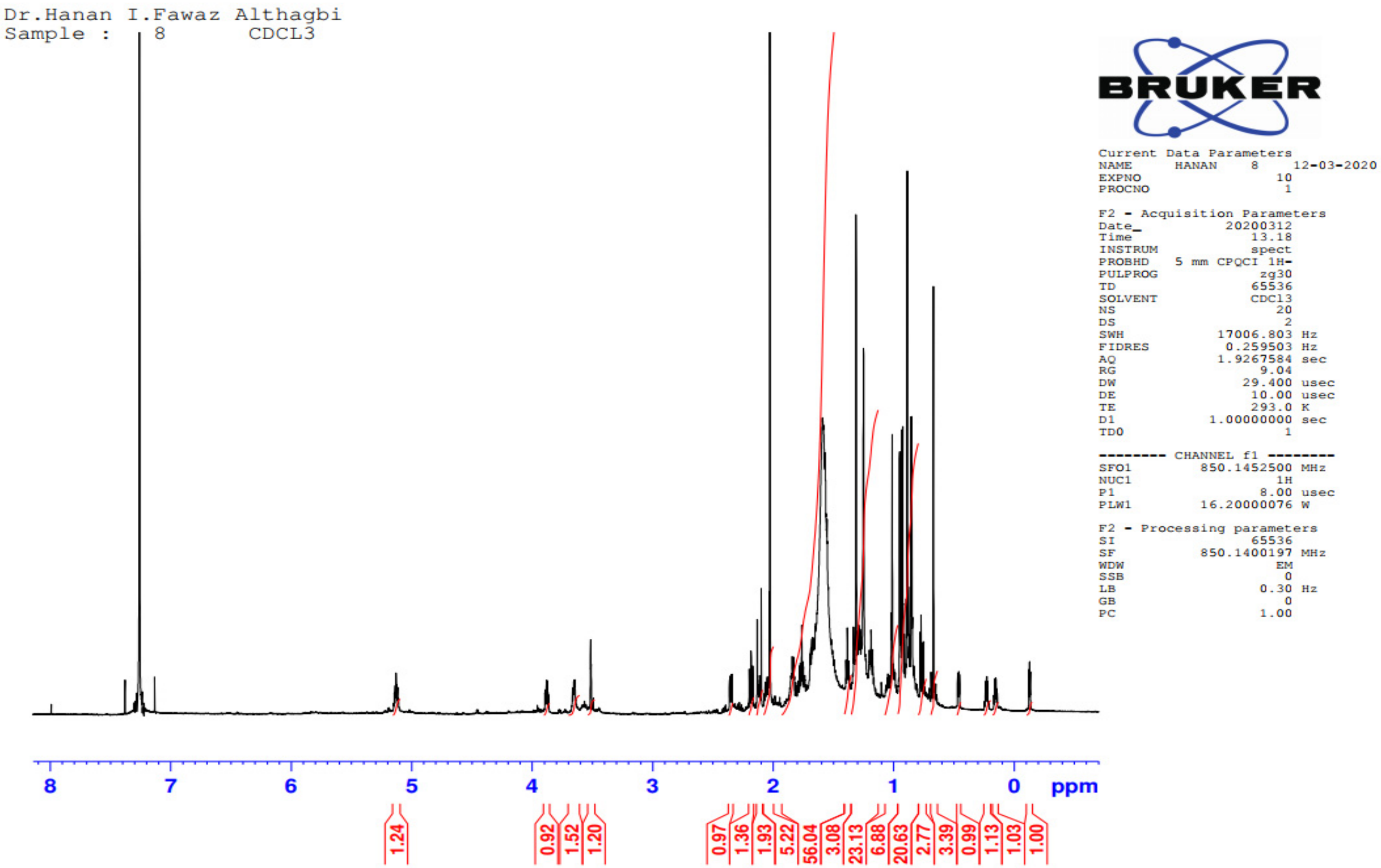


**Figure S3a.** $^{1}H$ NMR spectrum of compound **7**

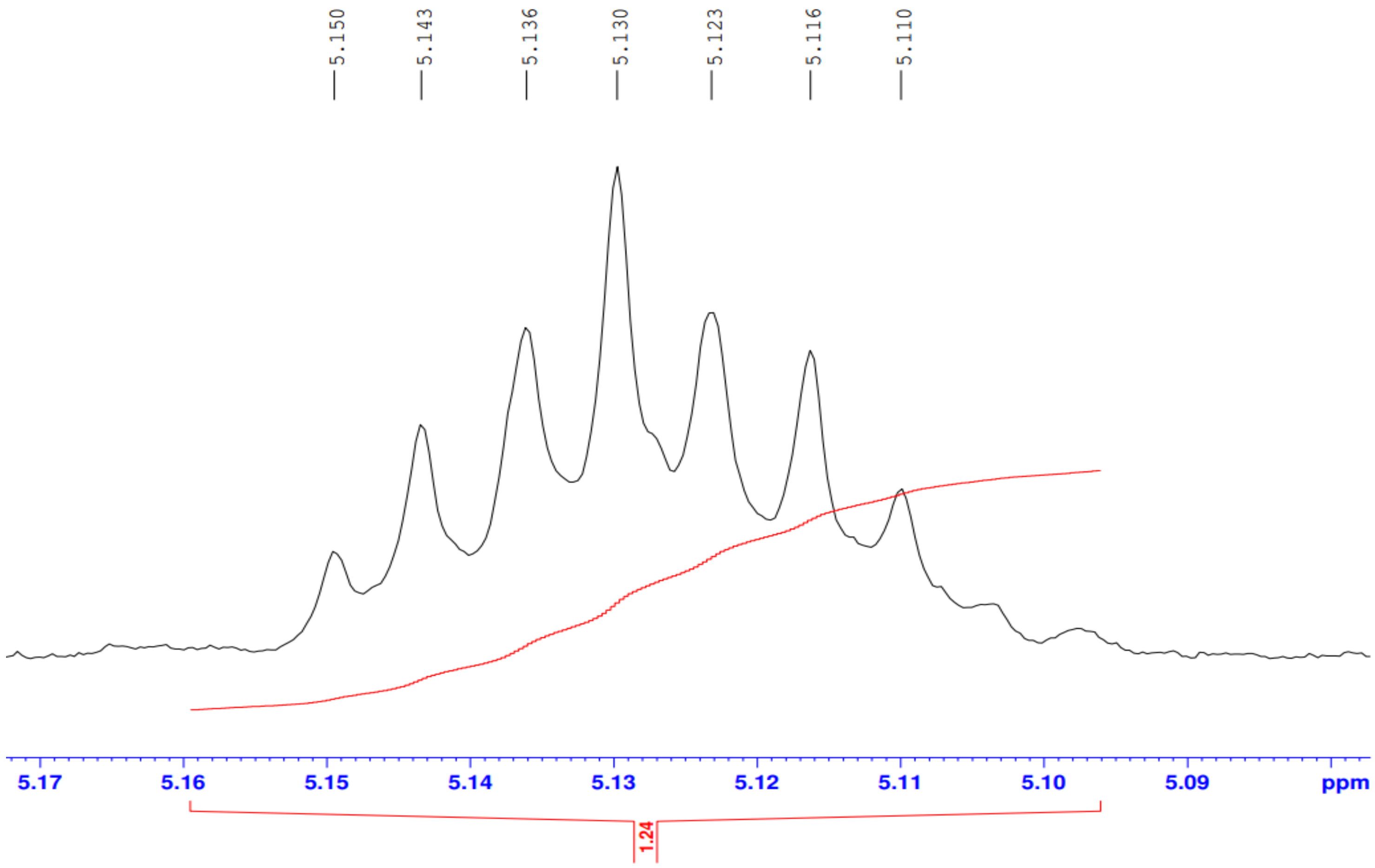


**Figure S3b.** $^{1}$H NMR spectrum of compound **7**

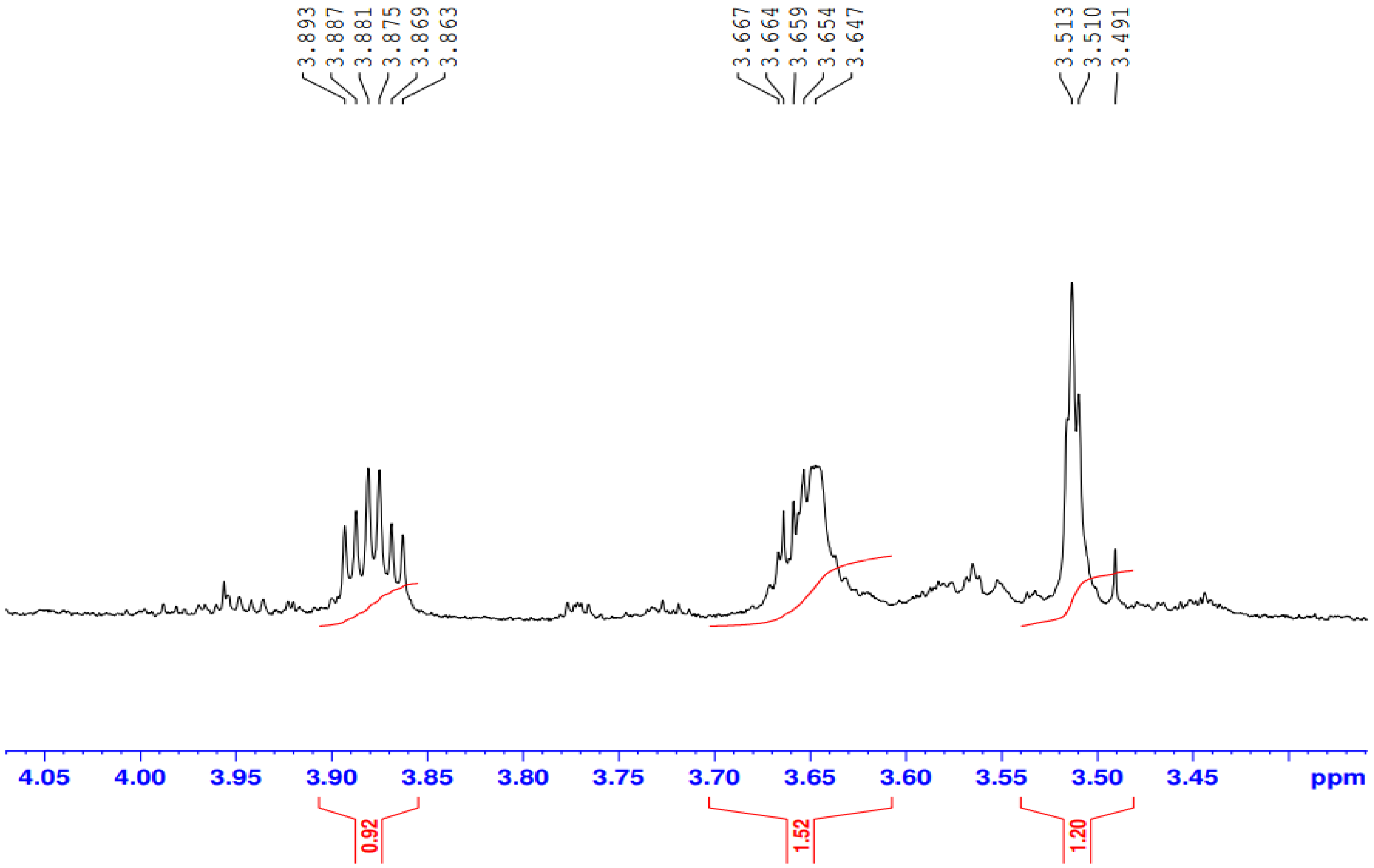


**Figure S3c.** $^{1}$H NMR spectrum of compound **7**

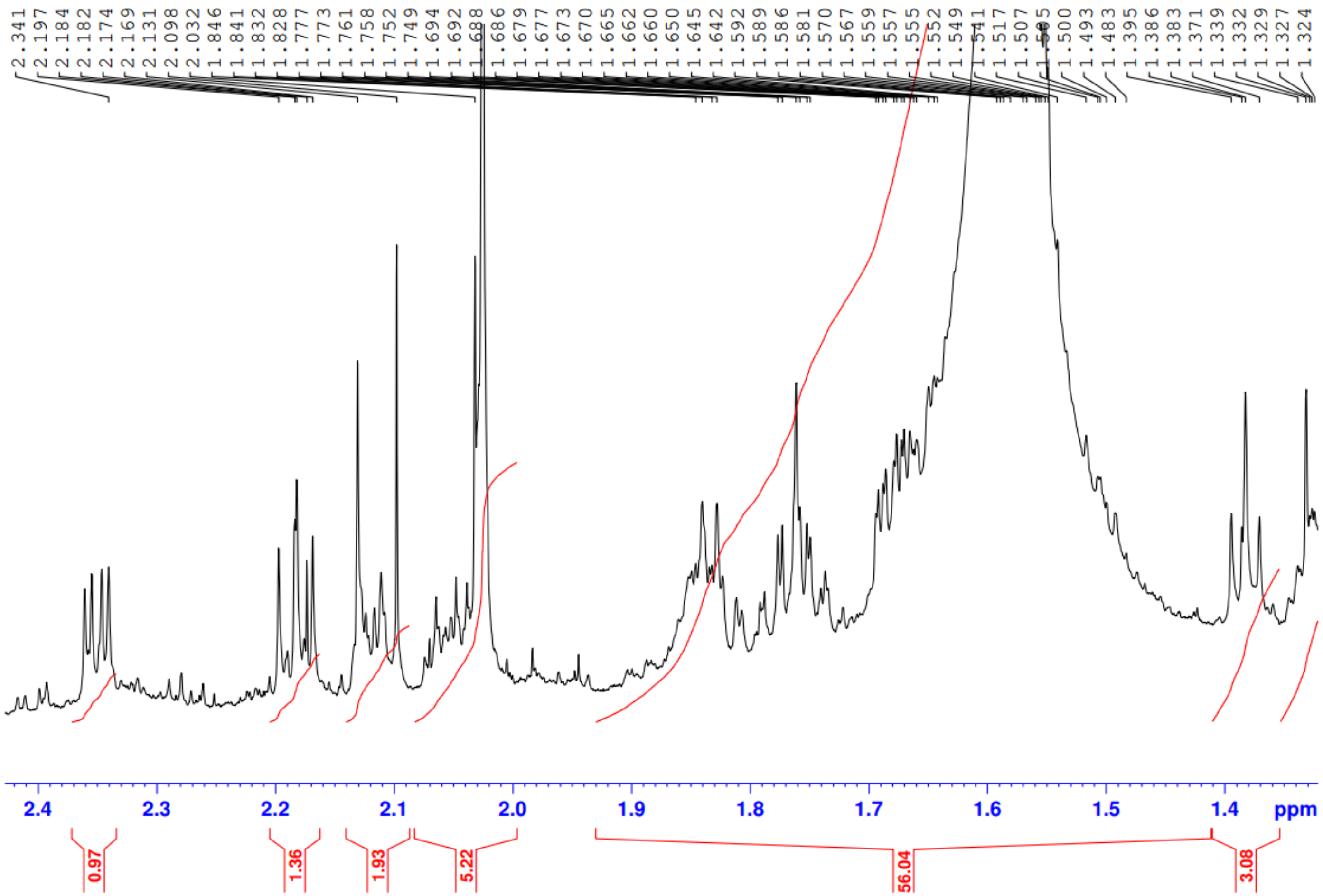

**Figure S3d.** $^{1}$H NMR spectrum of compound **7**

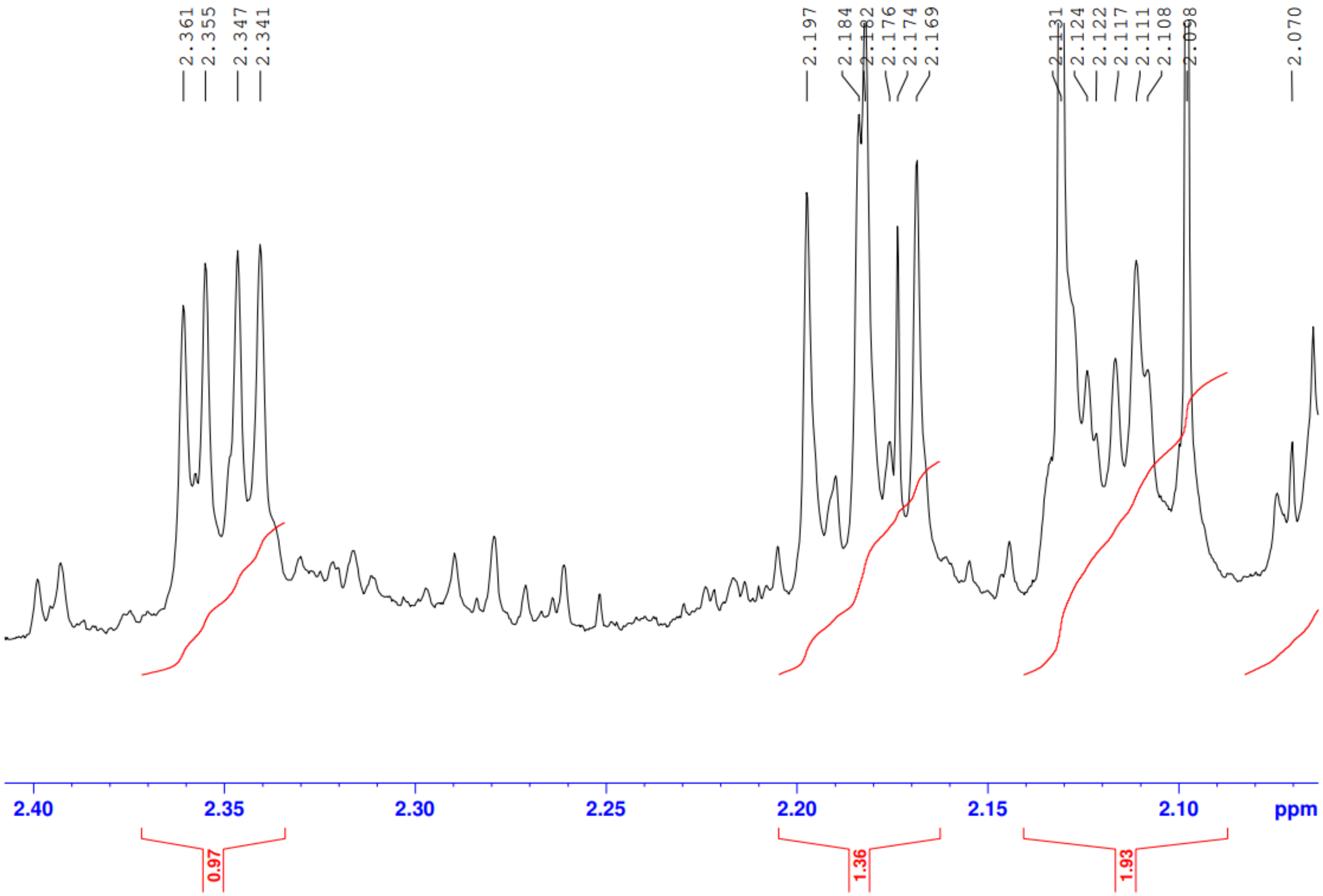


**Figure S3e.** $^{1}$H NMR spectrum of compound **7**

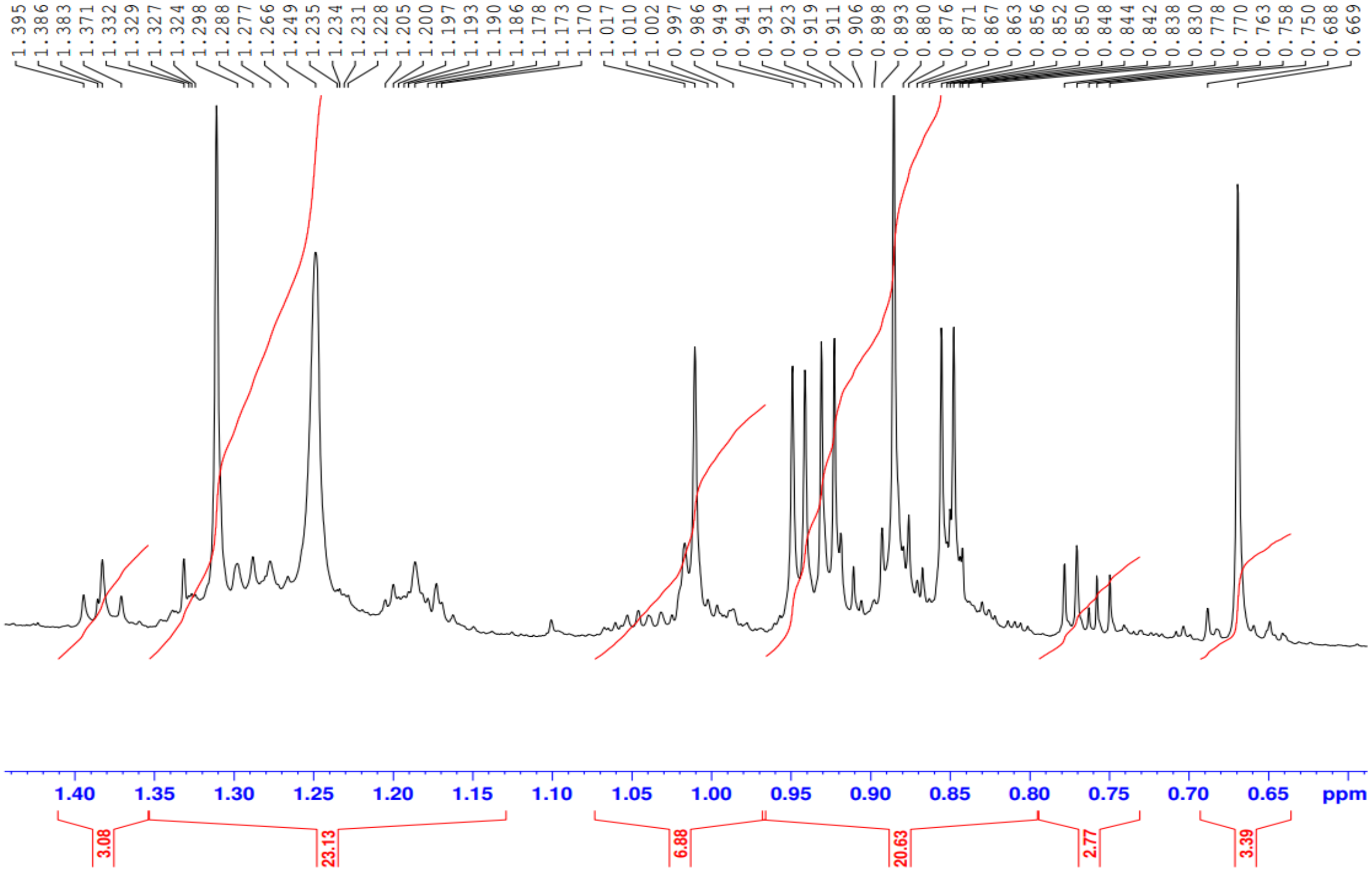

**Figure S3f.** $^1$H NMR spectrum of compound **7**

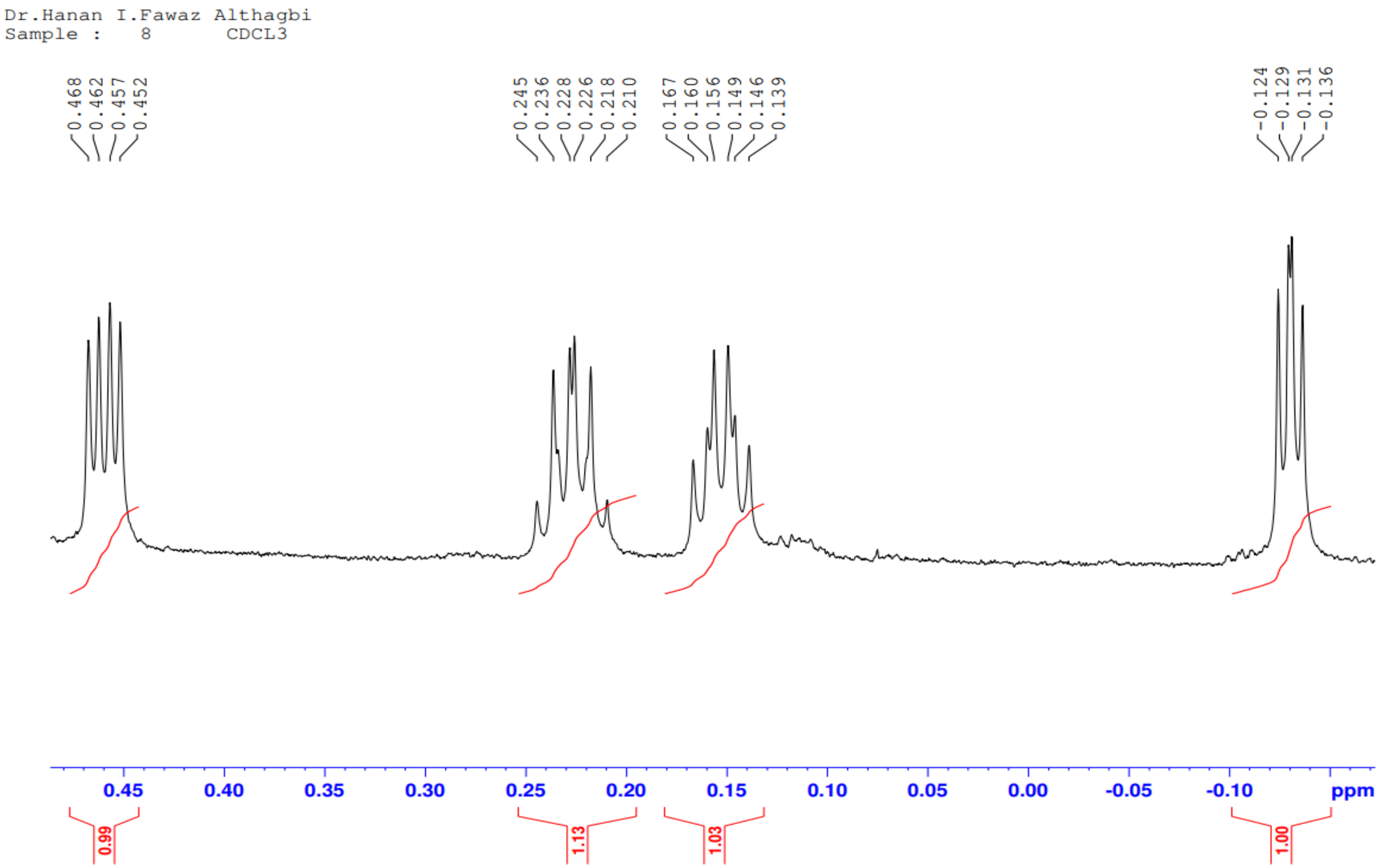


**Figure S3g.** $^{1}$H NMR spectrum of compound **7**

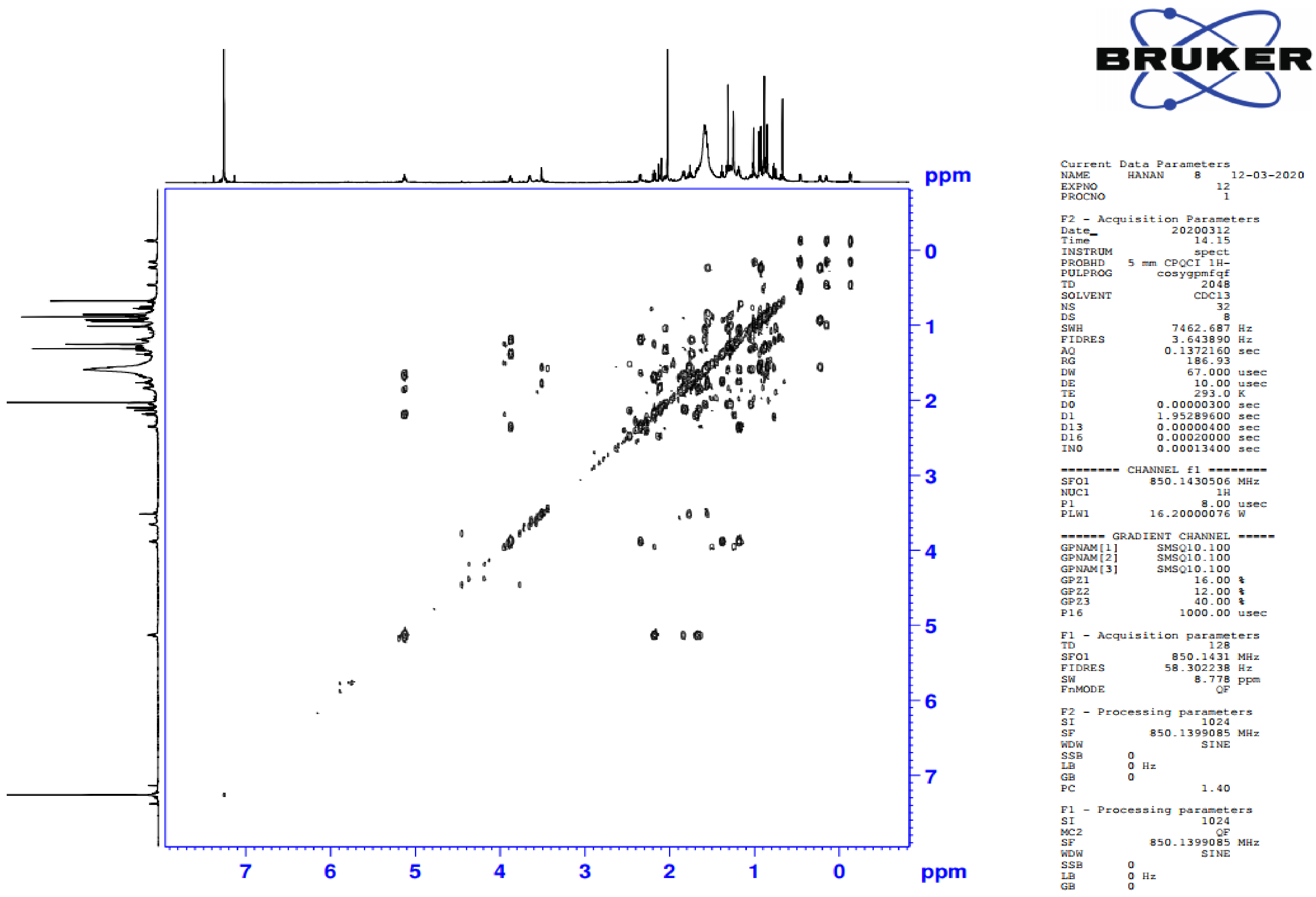


**Figure S3h.** $^{1}H$-$^{1}H$ COSY NMR spectrum of compound **7**

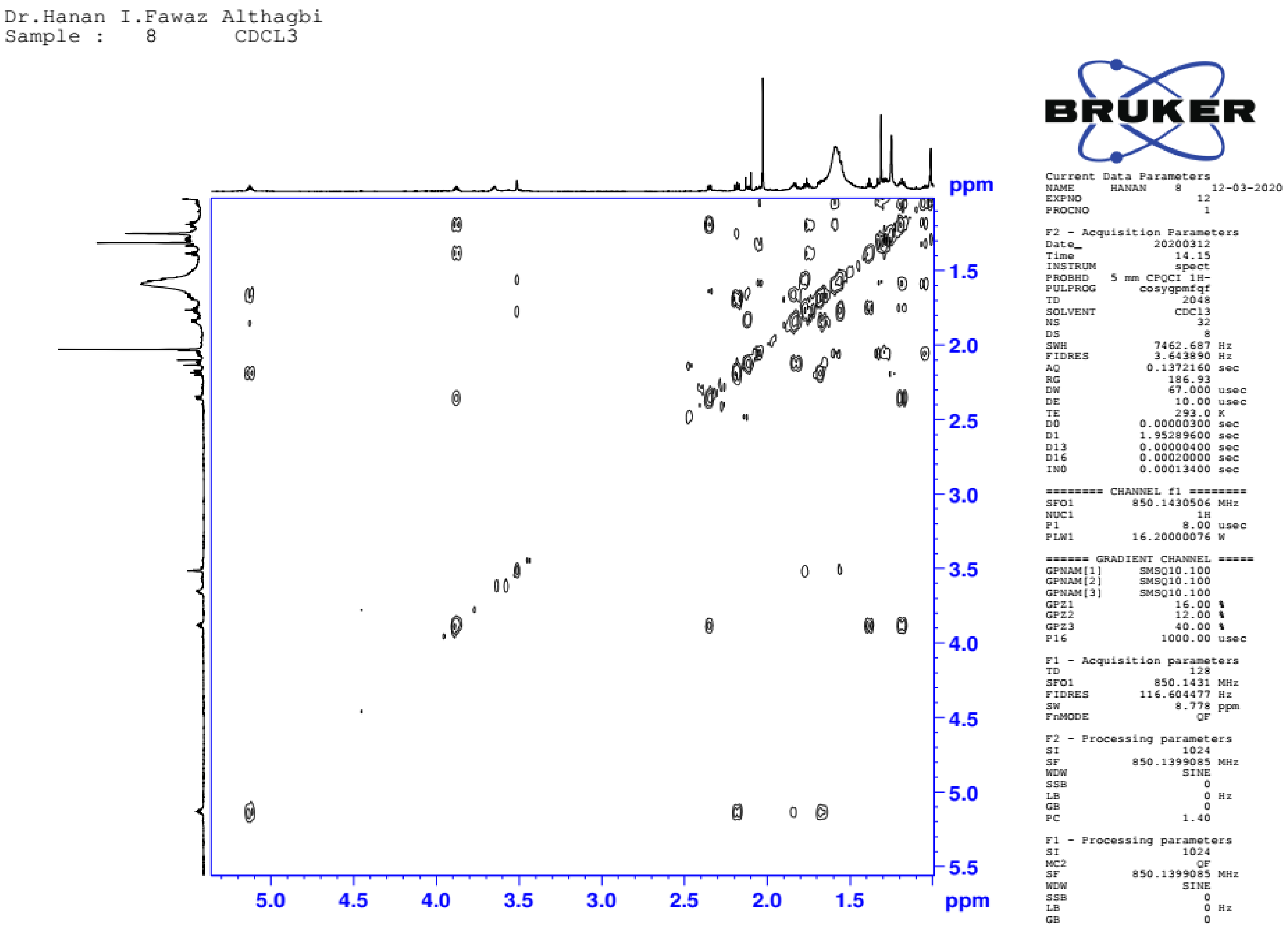


**Figure S3i.** $^{1}H$-$^{1}H$ COSY NMR spectrum of compound **7**

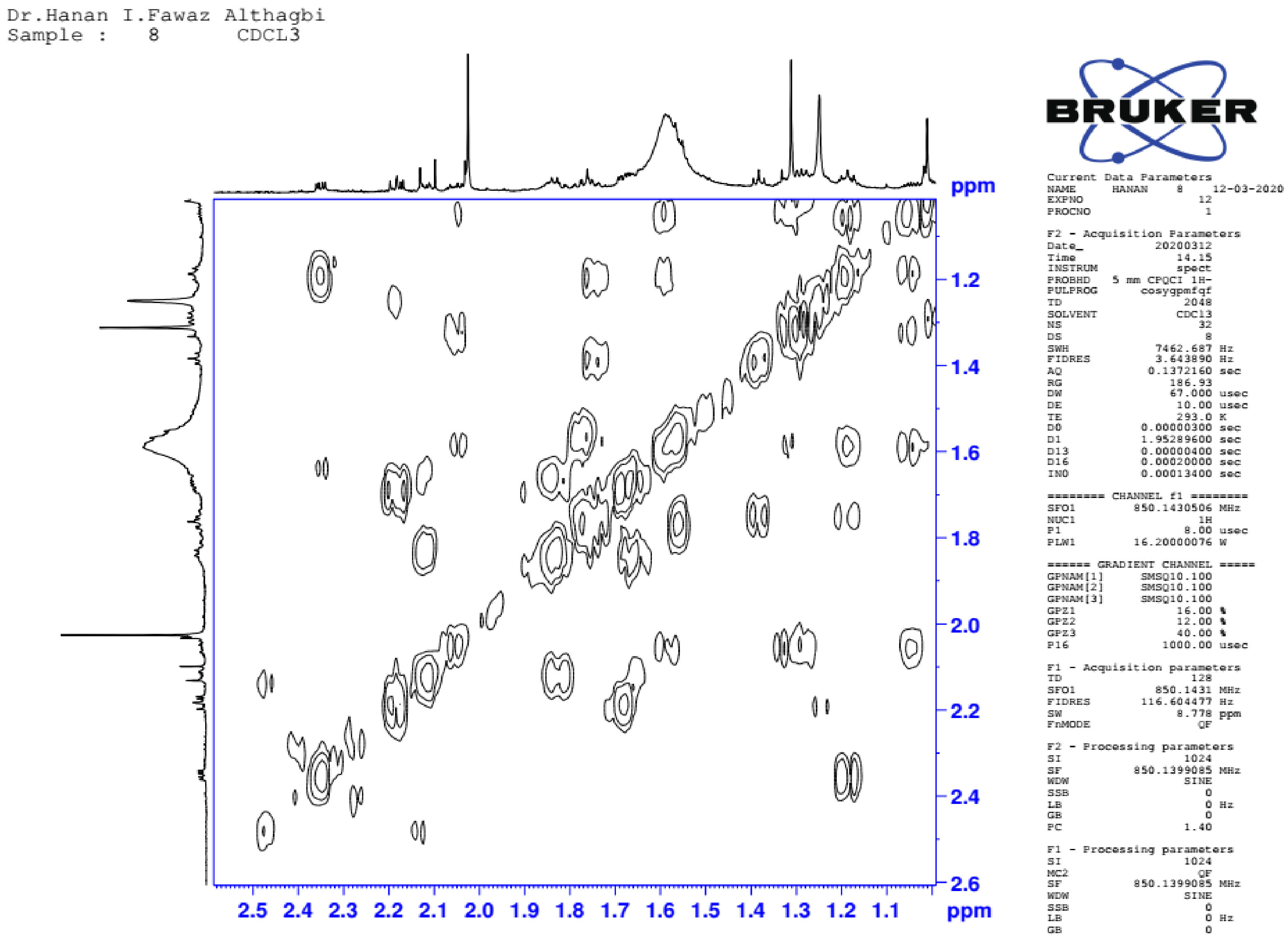


**Figure S3j.** $^{1}$H-$^{1}$H COSY NMR spectrum of compound **7**

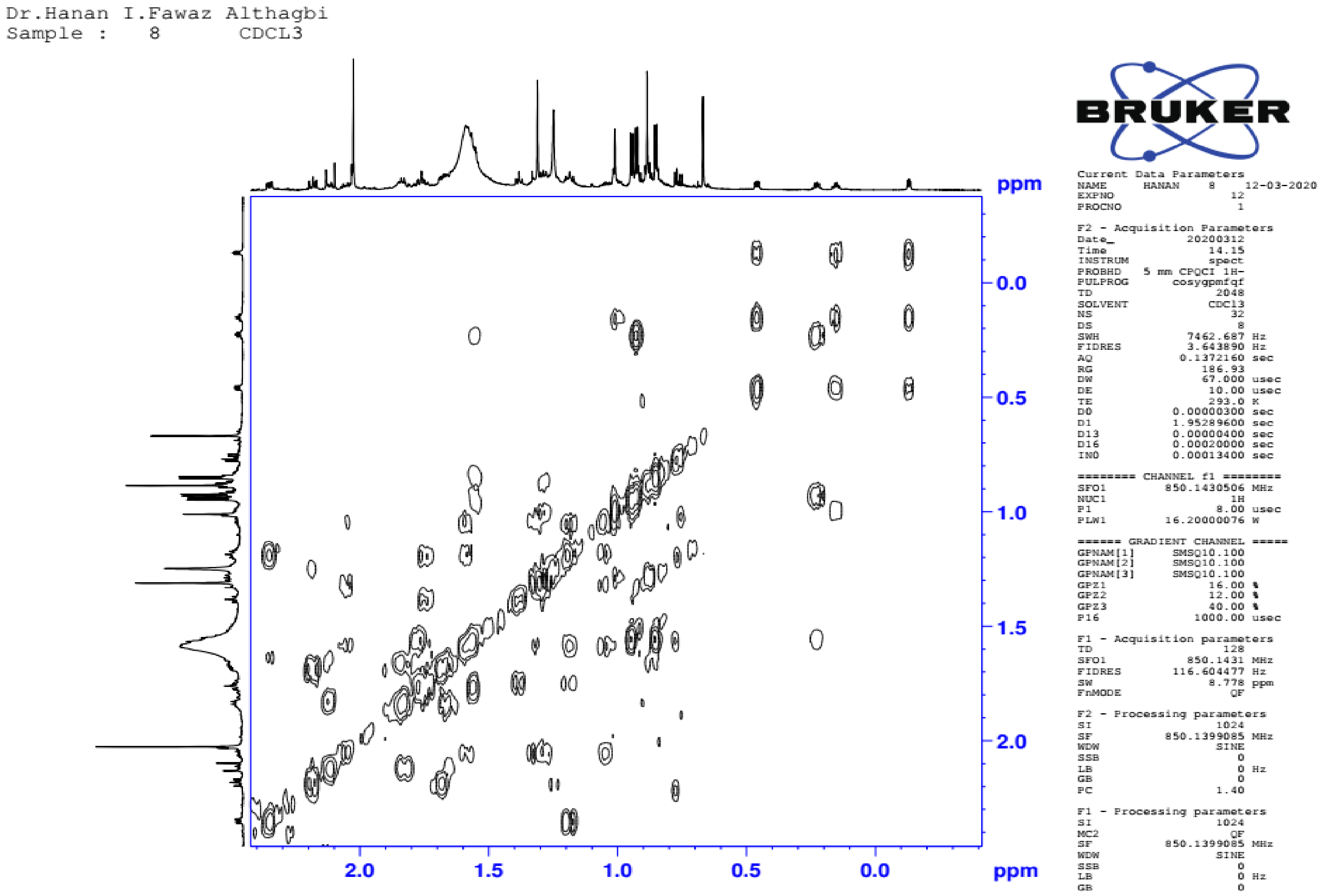


**Figure S3k.** $^{1}H$-$^{1}H$ COSY NMR spectrum of compound **7**)

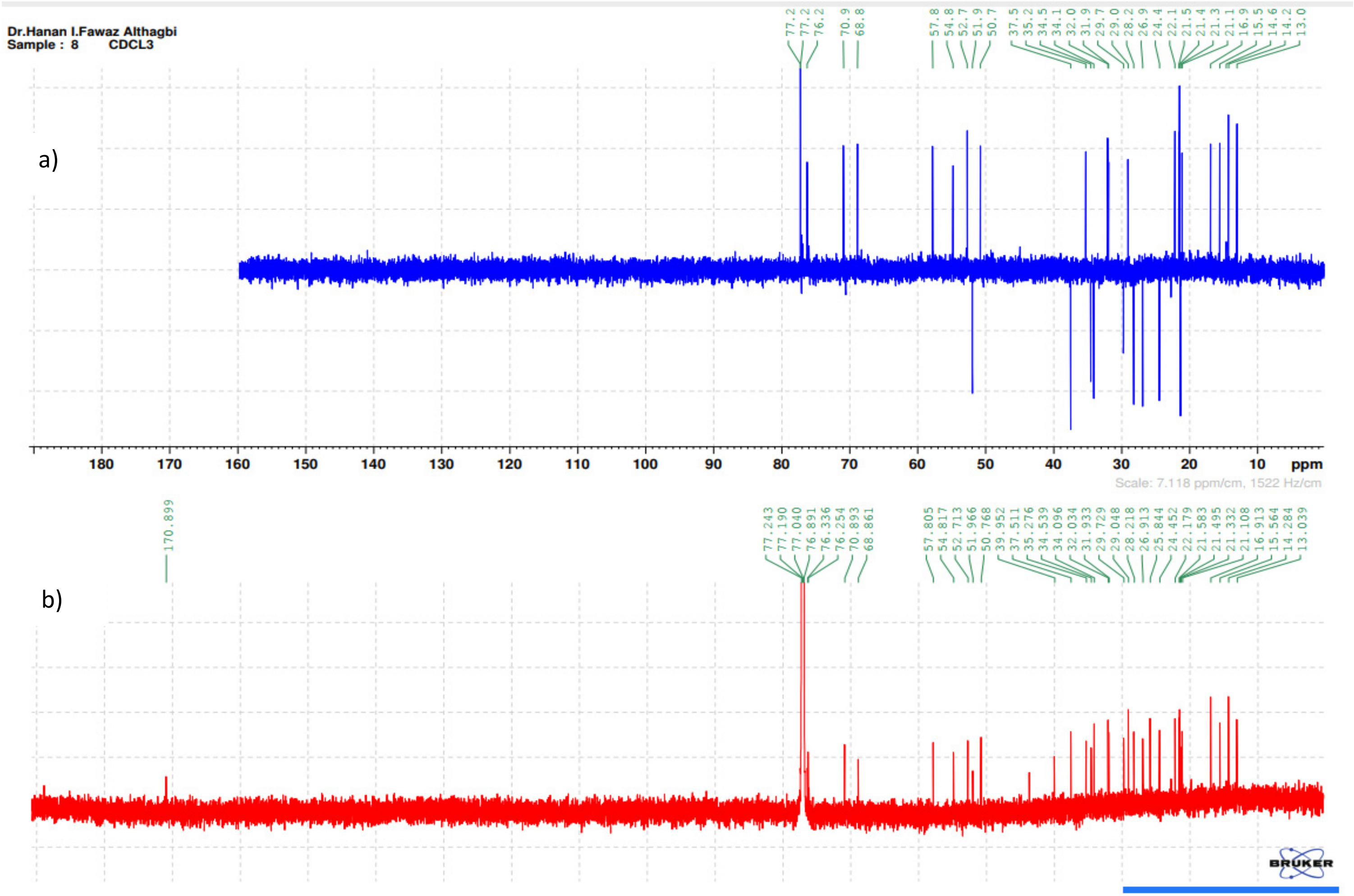


**Figure S3l.** a) $^{13}C$ DEPT-135 NMR and b) $^{13}C$ NMR spectra of compound **7**

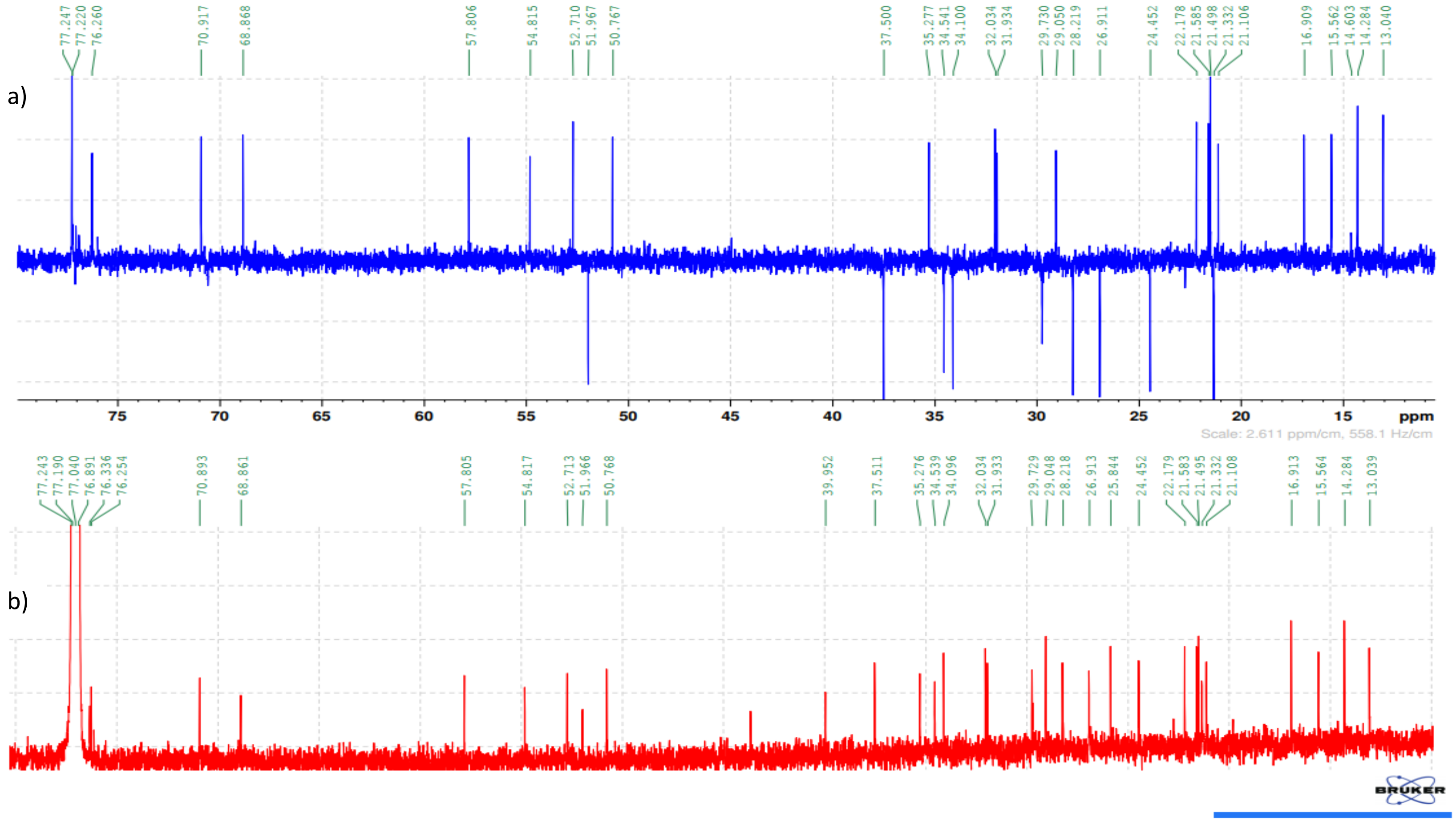


**Figure S3m.** a) $^{13}C$ DEPT-135 NMR and b) $^{13}C$ NMR spectra of compound **7**

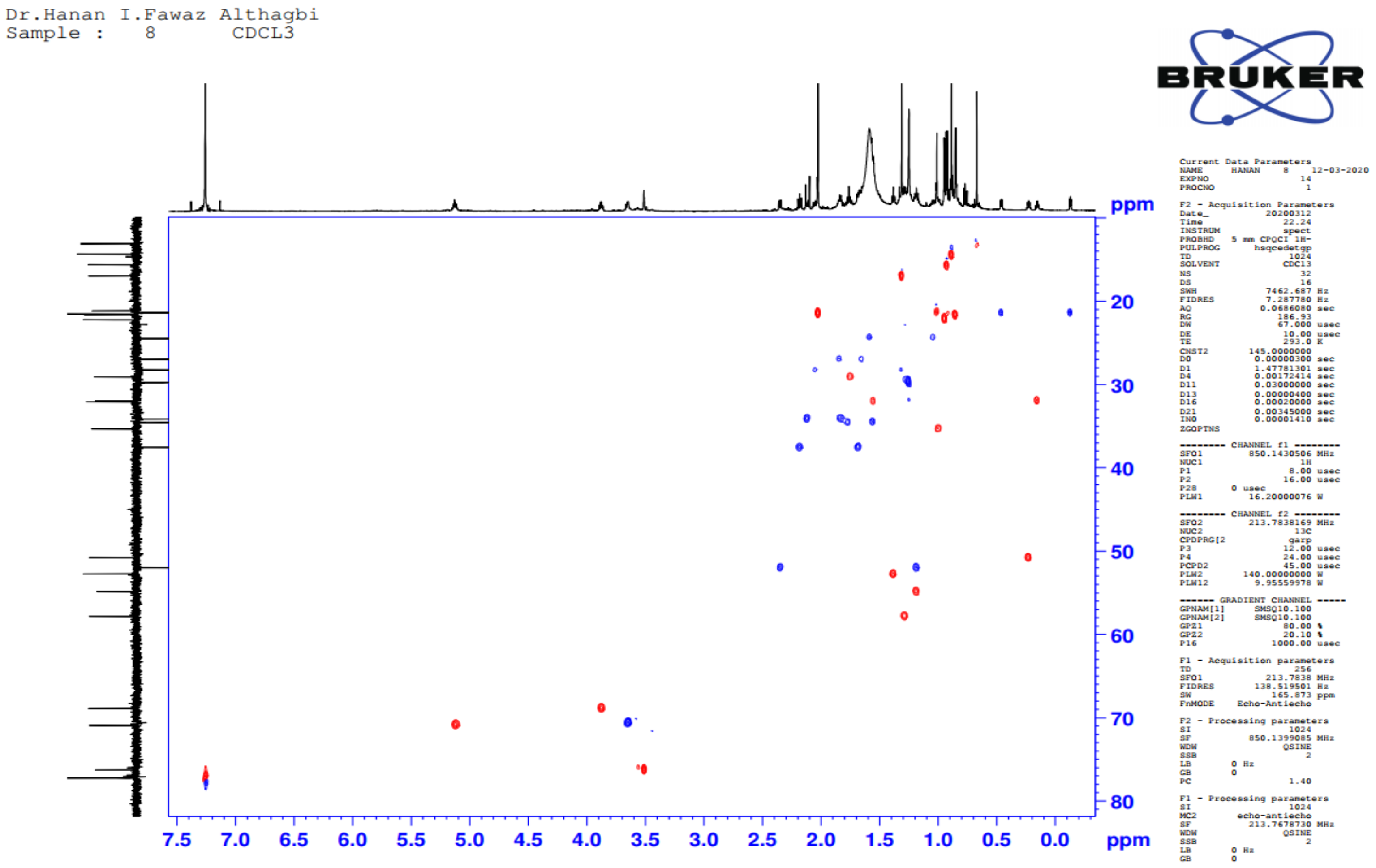


**Figure S3n.** $^{1}H$-$^{13}C$ HSQC NMR spectrum of compound **7**

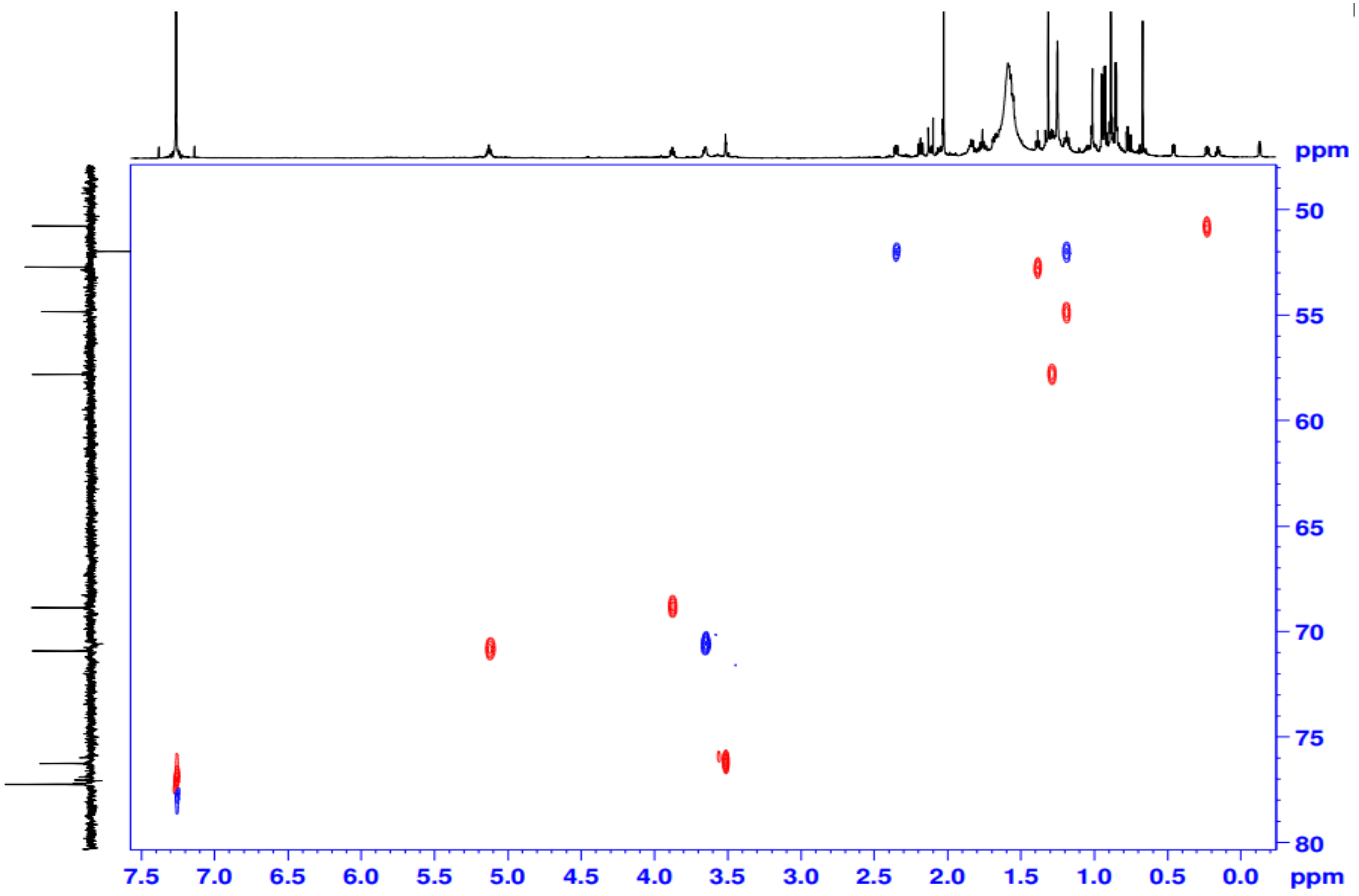


**Figure S3o.** $^{1}H$-$^{13}C$ HSQC NMR spectrum of compound **7**

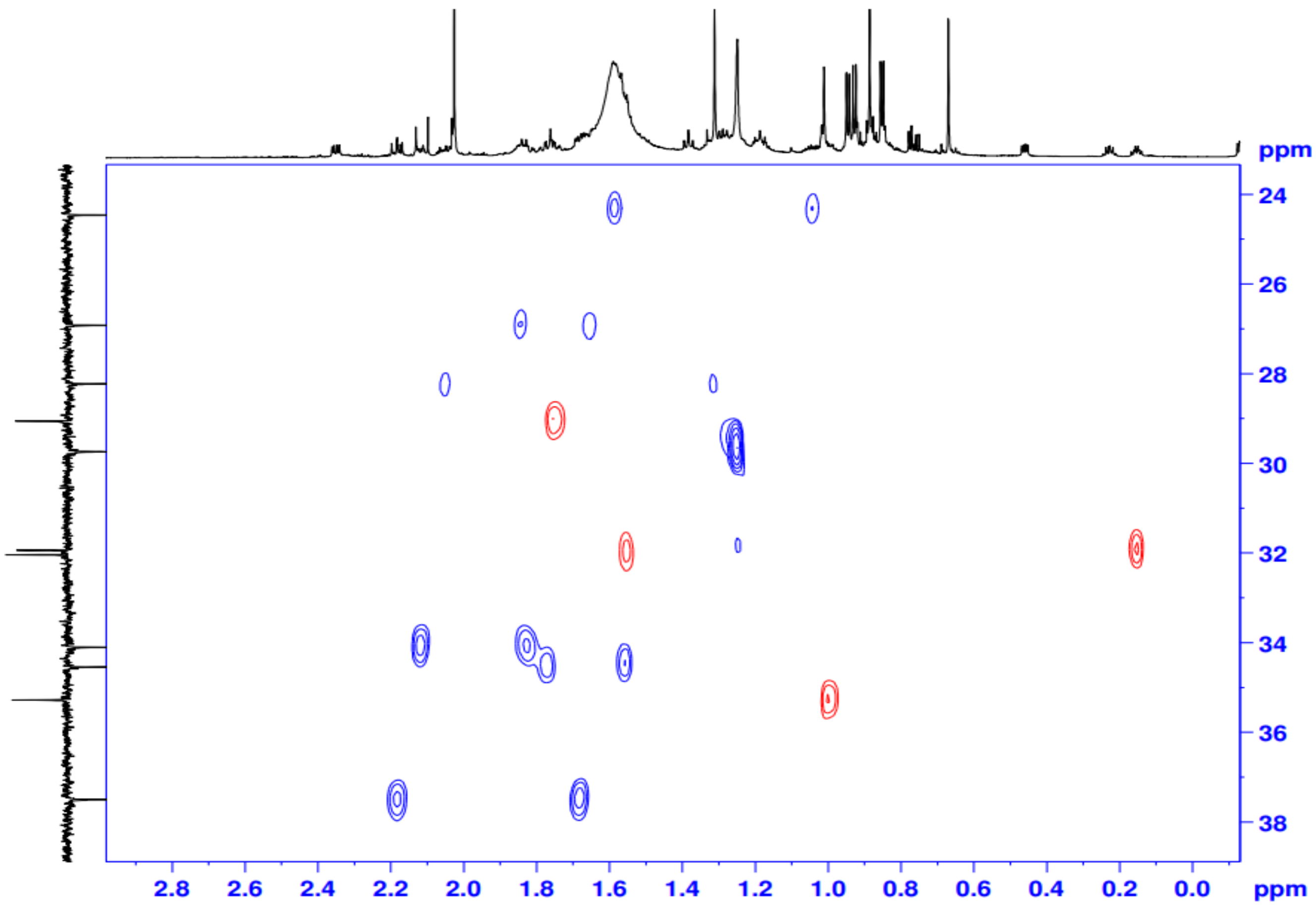


**Figure S3p.** $^{1}H$-$^{13}C$ HSQC NMR spectrum of compound **7**

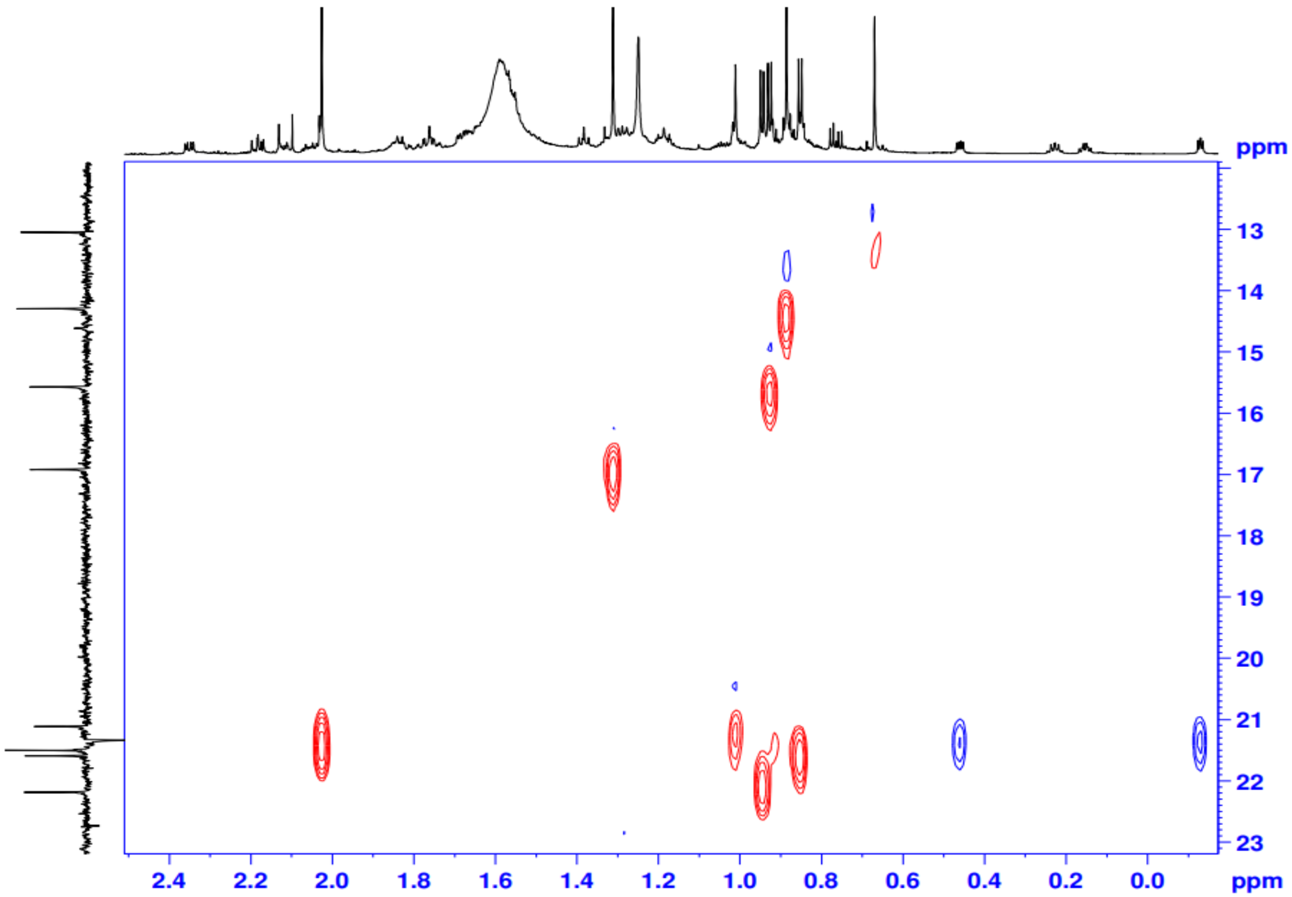


**Figure S3q.** $^{1}H$-$^{13}C$ HSQC NMR spectrum of compound **7**

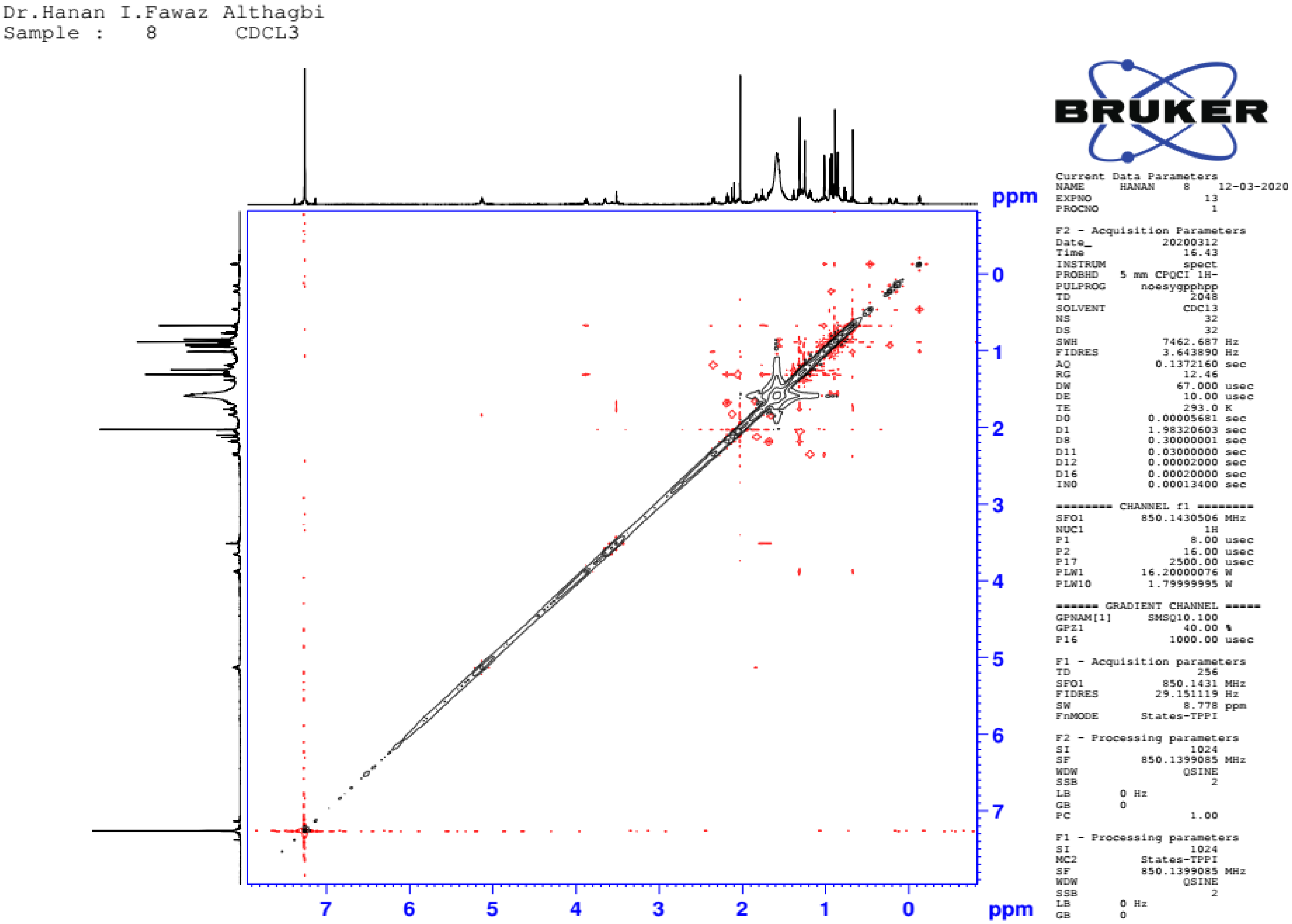


**Figure S3r.** $^{1}H$-$^{1}H$ NOESY NMR spectrum of compound **7**

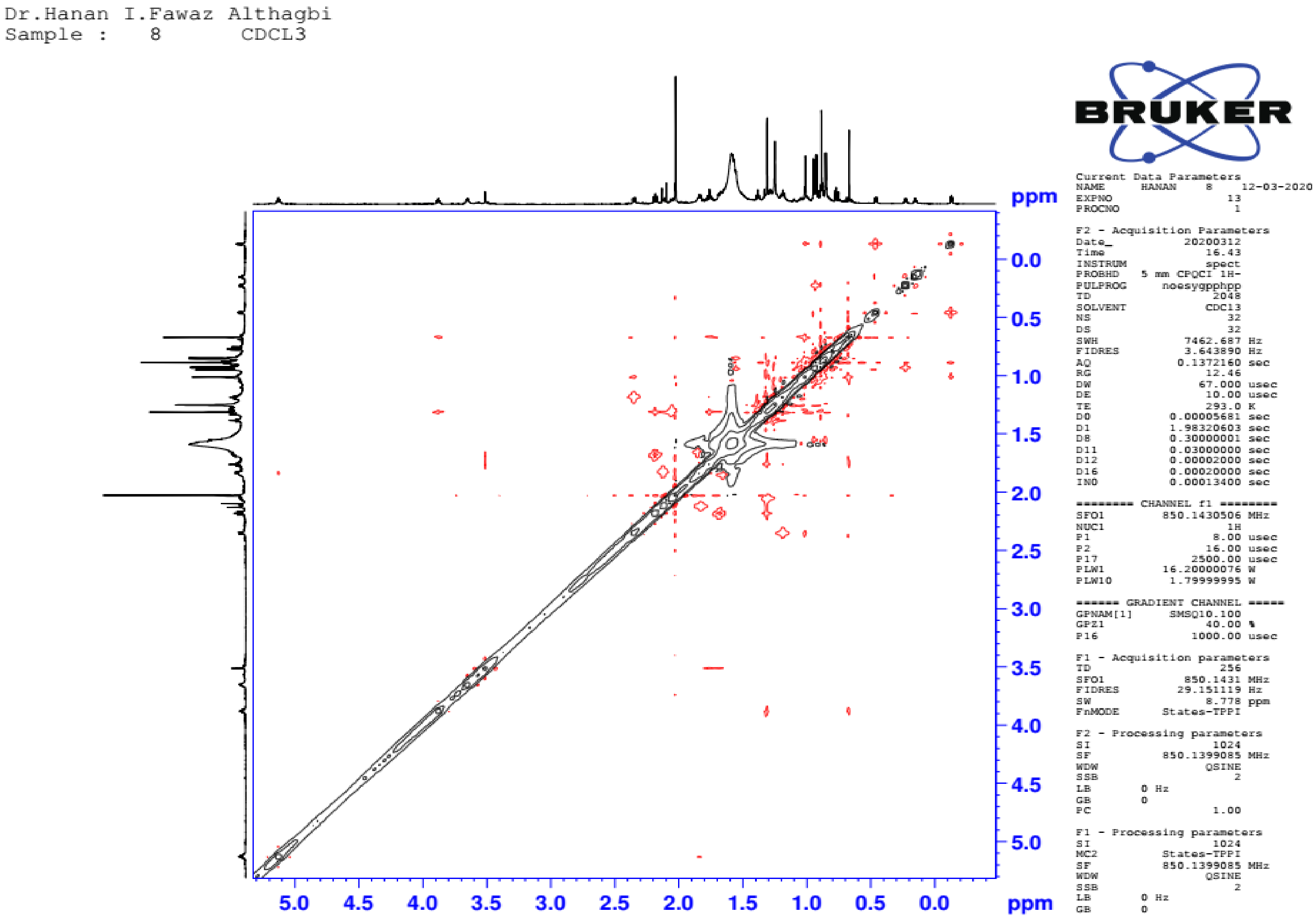


**Figure S3s.** $^{1}$H-$^{1}$H NOESY NMR spectrum of compound **7**

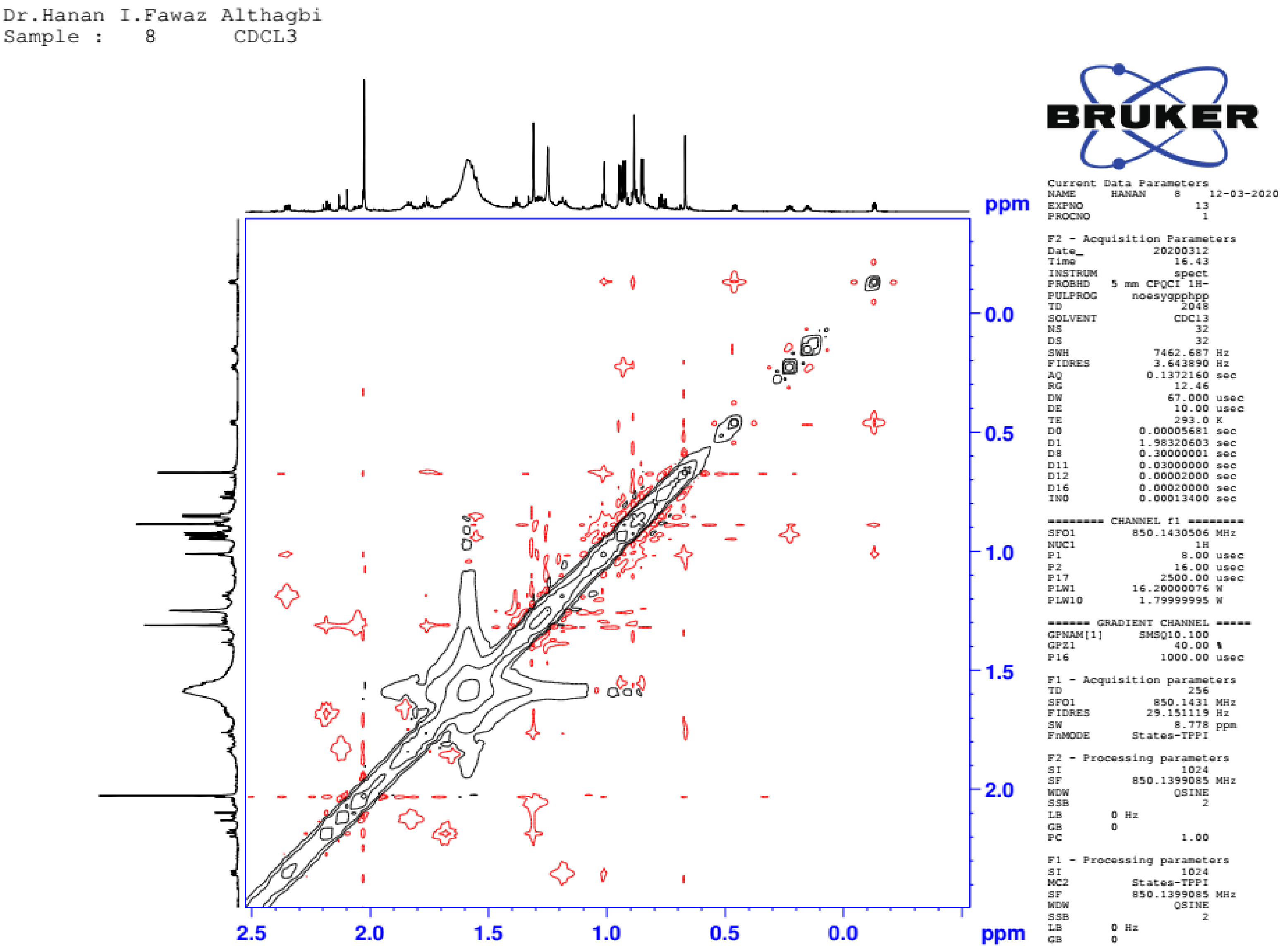


**Figure S3t.** $^{1}H$-$^{1}H$ NOESY NMR spectrum of compound **7**

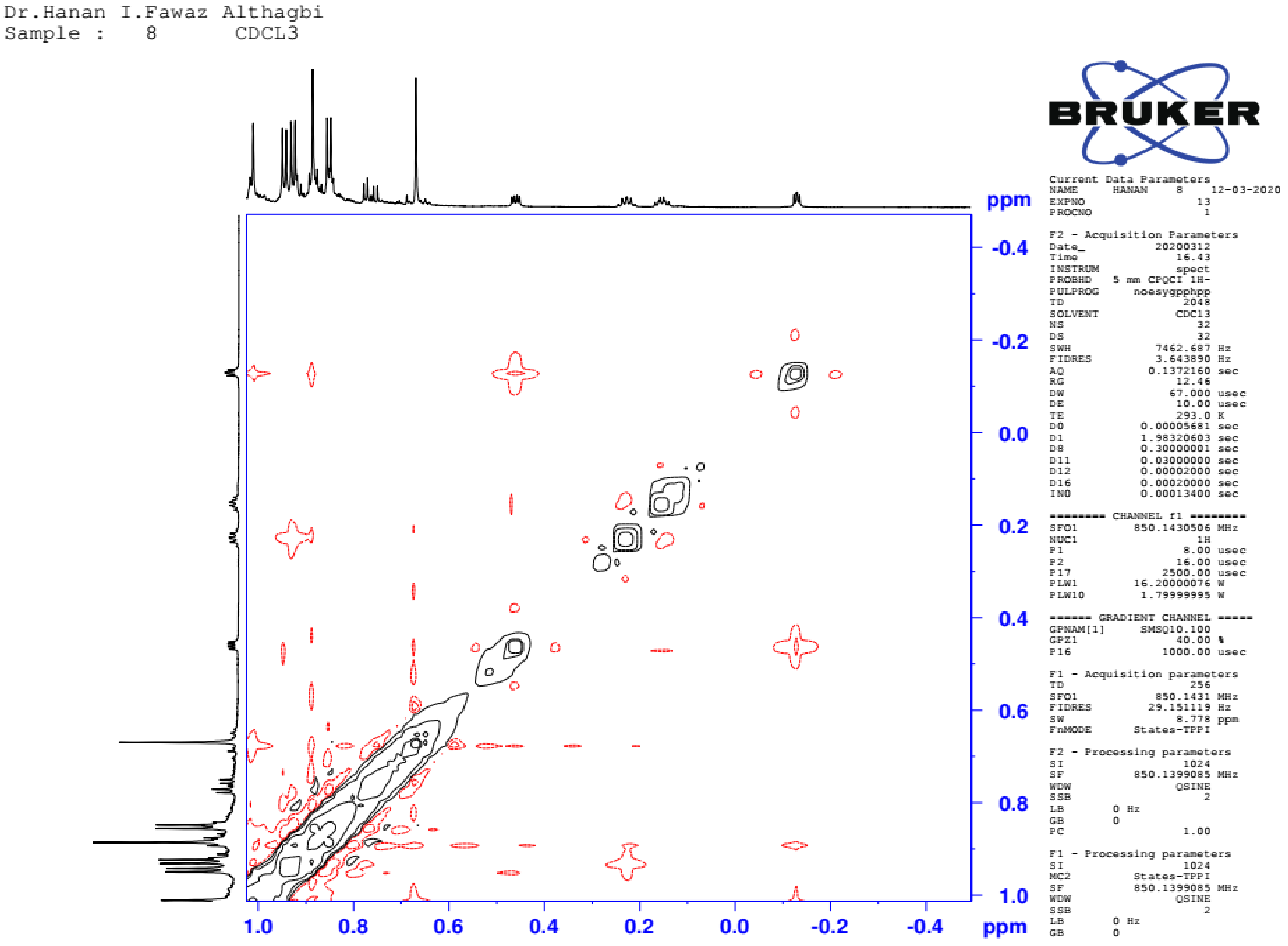


**Figure S3u.** $^1H$-$^1H$ NOESY NMR spectrum of compound **7**

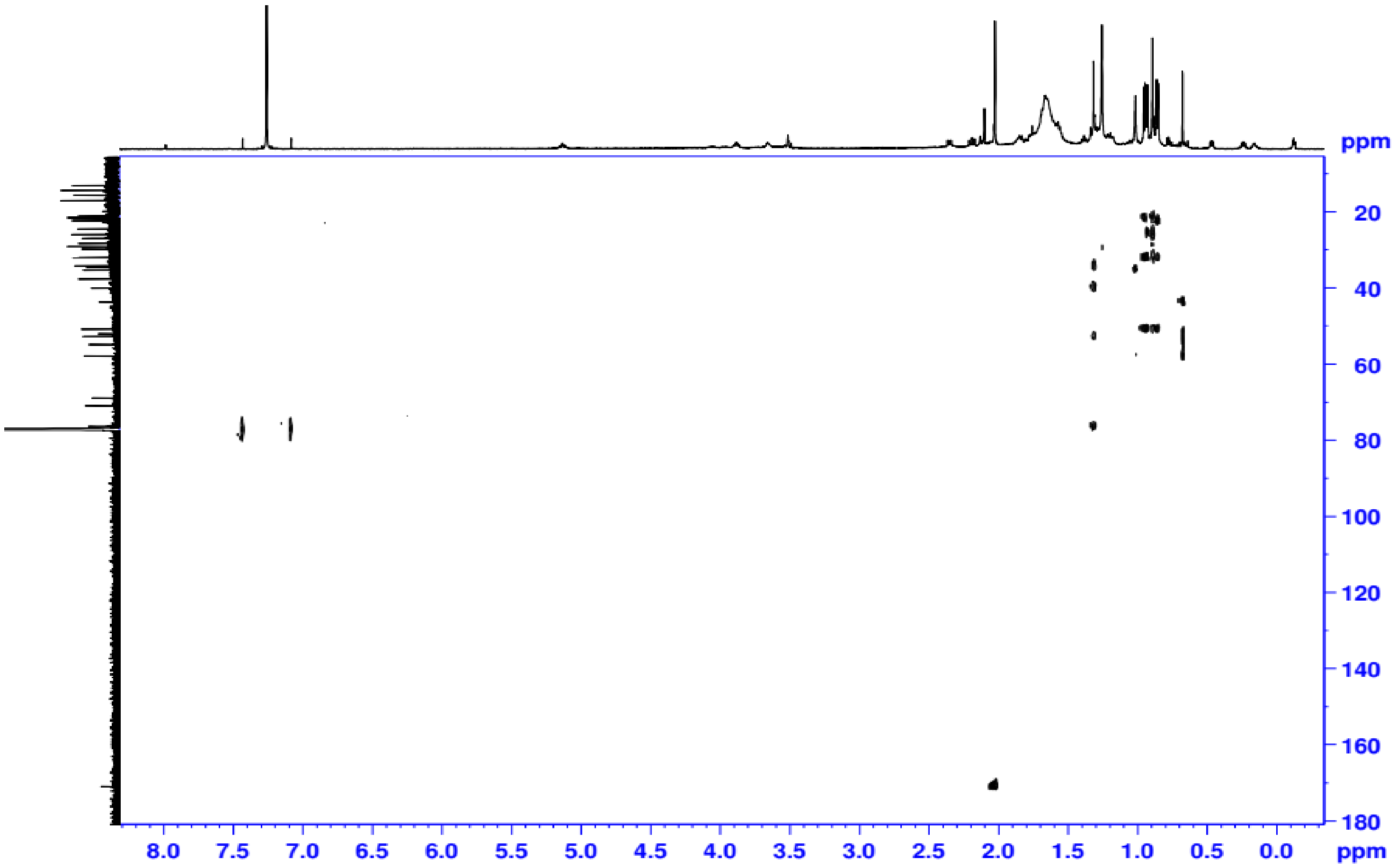


**Figure S3v.** $^{1}H$-$^{13}C$ HMBC NMR spectrum of compound **7**

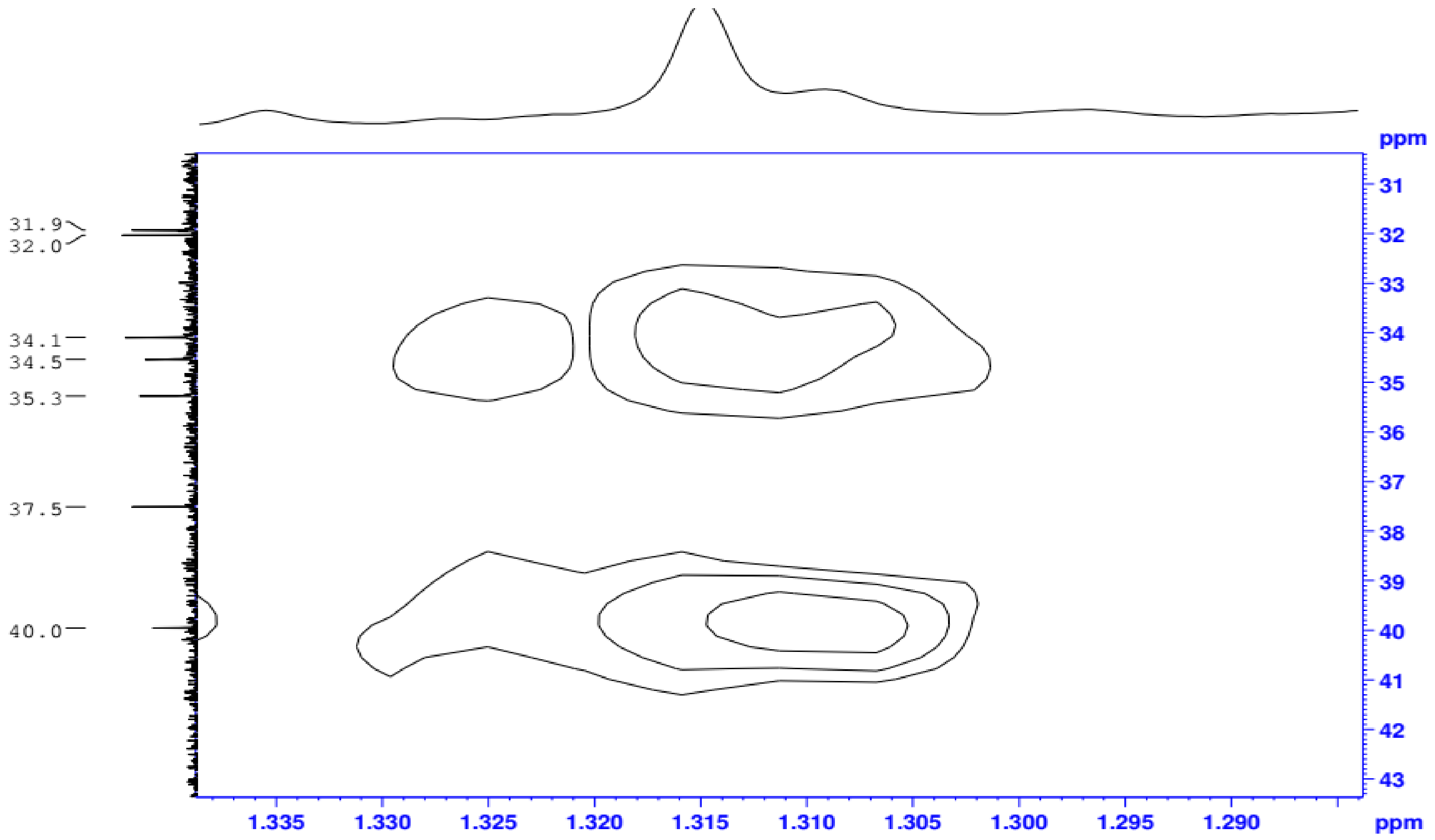


**Figure S3w.** $^{1}H$-$^{13}C$ HMBC NMR spectrum of compound **7**

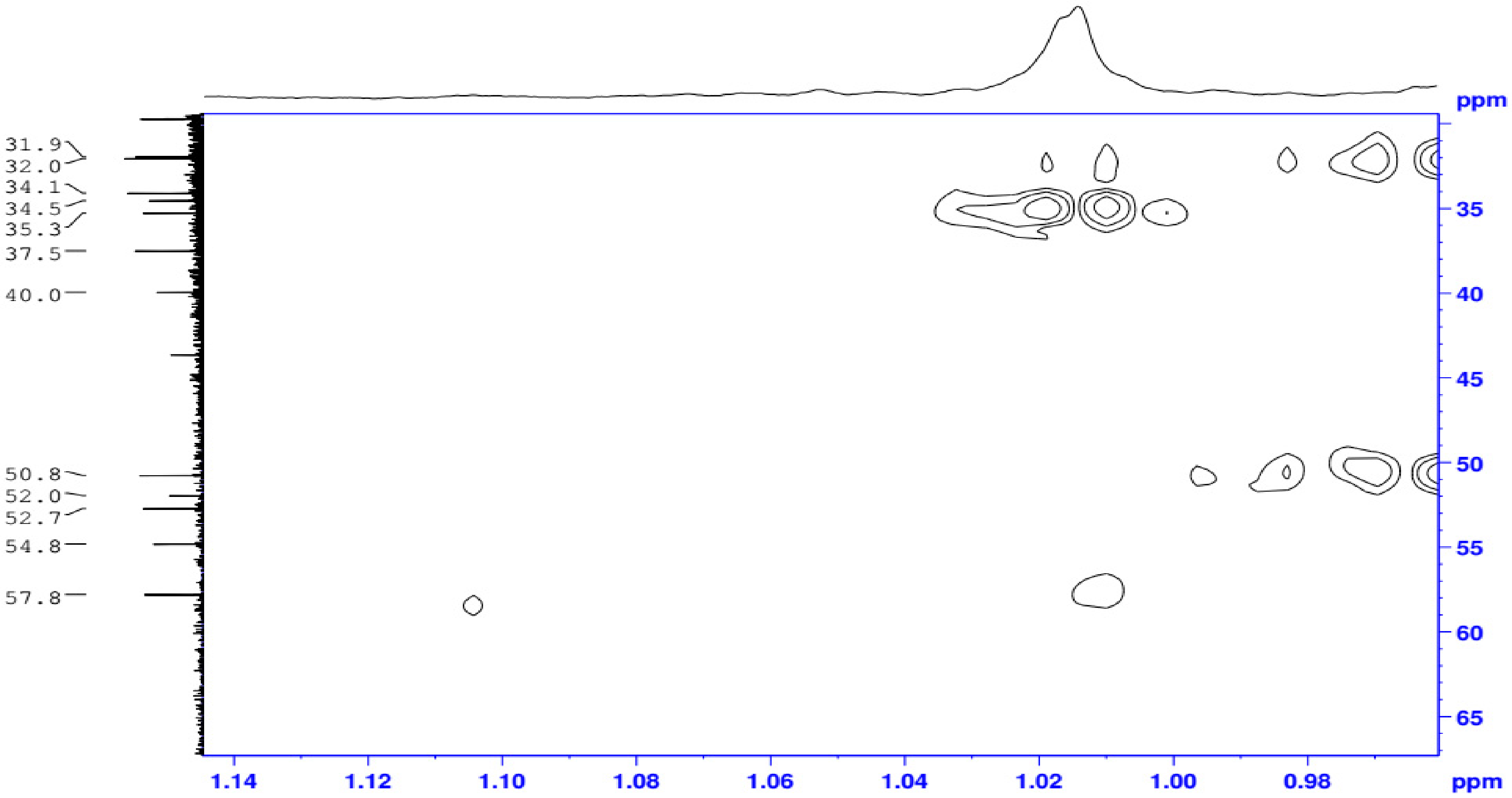


**Figure S3x.** $^{1}H$-$^{13}C$ HMBC NMR spectrum of compound **7**

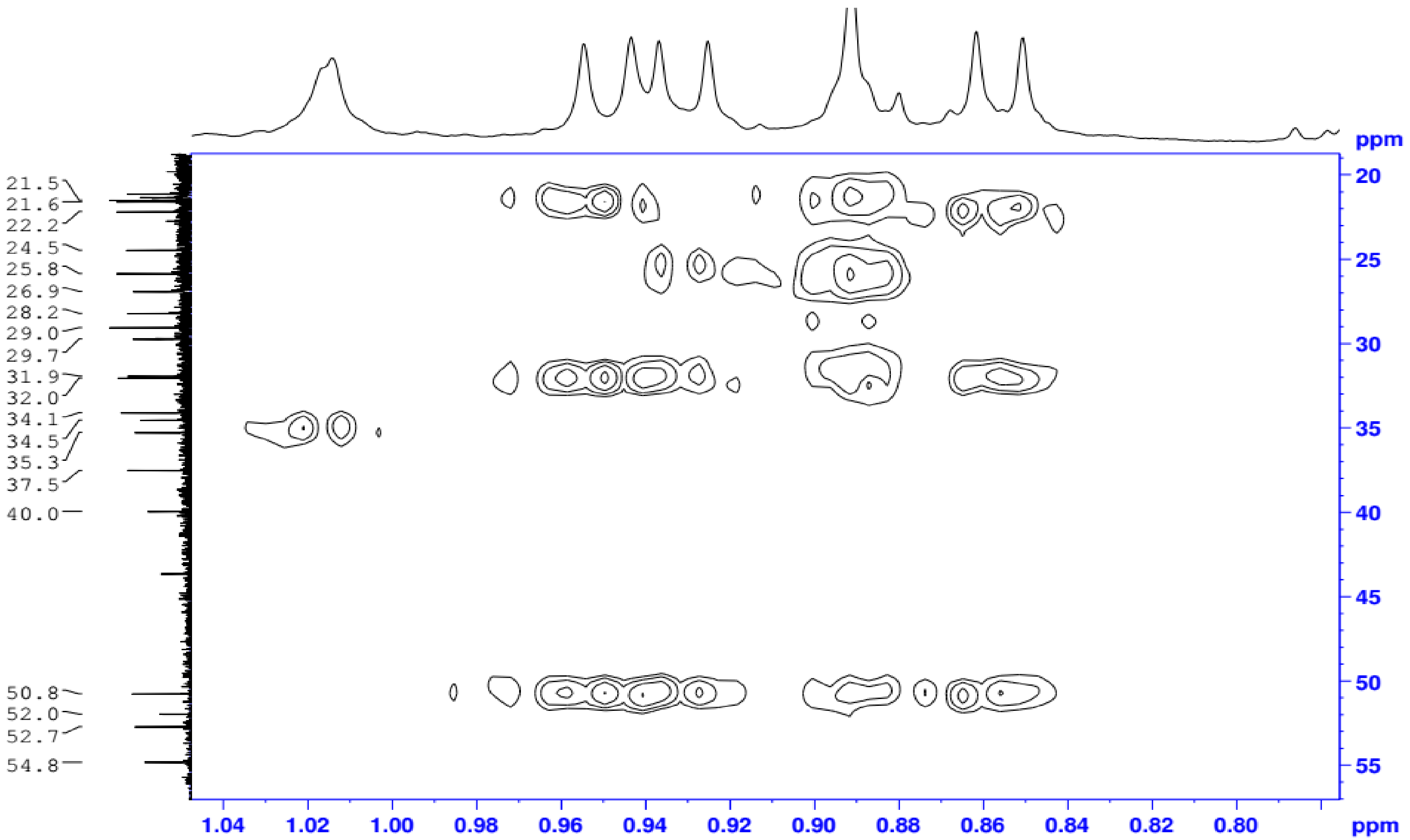


**Figure S3y.** $^{1}H$-$^{13}C$ HMBC NMR spectrum of compound **7**

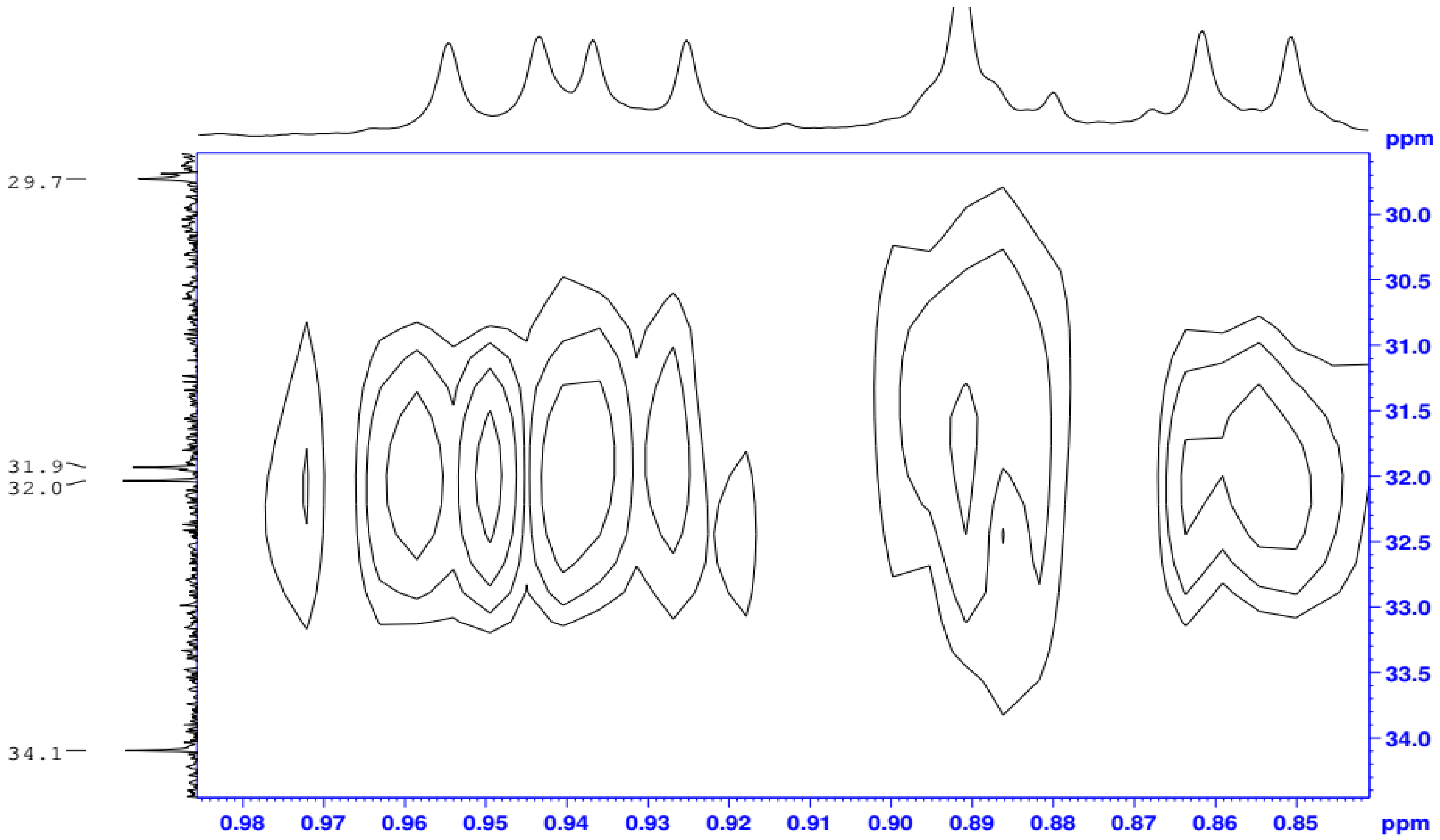


**Figure S3z.** $^{1}H$-$^{13}C$ HMBC NMR spectrum of compound **7**
